# Supplementary material for: Fast activity prediction of chemically modified siRNAs via structure-based energy calculations and inference-augmented tabular deep learning
Source: Mol Ther Nucleic Acids. 2026 Jul 21;37(3):103029. doi: 10.1016/j.omtn.2026.103029 (PMC13488036; doi:10.1016/j.omtn.2026.103029)
Supplement: Supplementary file 1 [file mmc1.pdf]

OMTN, Volume *37*

Supplemental information

Fast activity prediction of chemically modified siRNAs via structure-based energy calculations and inference-augmented tabular deep learning

Wenchong Tan, Yiheng Dong, Yuanfang Shi, Nanwen Chen, Shimin Ye, Heng Zhang, Ping Chen, Yinglin Zuo, and Hongli Du

Supplemental Results

To pinpoint the origin of the performance gains achieved via Inference Augmentation under leave-one-out cross-validation (LOOCV), we algebraically decomposed the R^2 metric. This analysis reveals that the substantial increase in R^2 stems primarily from improved prediction calibration, rather than solely from enhanced ranking correlation over strong baselines.

Using the standard coefficient of determination, $R^2 = 1 - \mathrm{SS}_{\mathrm{res}}/\mathrm{SS}_{\mathrm{tot}}$, let y and $\hat{y}$ denote the vectors of experimental and predicted values, respectively. The mean squared error (MSE) expands as Equation S1:

$$\mathrm{MSE} = \frac{1}{N}\sum_{i=1}^{N}(y_i - \hat{y}_i)^2 = \sigma_{\hat{y}}^2 + \sigma_y^2 - 2\mathrm{Cov}(y, \hat{y}) + (\bar{\hat{y}} - \bar{y})^2$$

Dividing by the variance of the experimental values σ_y^2, we obtain Equation S2:

$$\frac{\mathrm{MSE}}{\sigma_y^2} = \left(\frac{\sigma_{\hat{y}}}{\sigma_y}\right)^2 + 1 - 2\left(\frac{\mathrm{Cov}(y, \hat{y})}{\sigma_y \sigma_{\hat{y}}}\right)\left(\frac{\sigma_{\hat{y}}}{\sigma_y}\right) + \left(\frac{\bar{\hat{y}} - \bar{y}}{\sigma_y}\right)^2$$

We define three interpretable components: (1) the Pearson correlation coefficient $r = \mathrm{Cov}(y, \hat{y})/(\sigma_y \sigma_{\hat{y}})$; (2) the variance matching factor (ratio of standard deviations) $k = \sigma_{\hat{y}}/\sigma_y$; and (3) the normalized mean bias $b = (\bar{\hat{y}} - \bar{y})/\sigma_y$. Substituting these into $R^2 = 1 - \mathrm{MSE}/\sigma_y^2$ yields Equation S3:

$$R^2 = 1 - (k^2 + 1 - 2rk + b^2) = 2rk - k^2 - b^2$$

While numerically identical to the standard definition, this formulation isolates the contributions of linear correlation (r), variance matching (k), and mean bias (b).[1,2] It clarifies why R^2 can improve far more than Pearson r when augmentation primarily corrects calibration. Specifically, Figure S15A details these components for TabPFN without augmentation ($R^2 \approx 0.09$, $r \approx 0.35$), PLS without augmentation ($R^2 \approx 0.40$, $r \approx 0.64$), and TabPFN with augmentation ($R^2 \approx 0.54$, $r \approx 0.74$). Without augmentation, TabPFN severely underestimates variance ($k \ll 1$), which penalizes its R^2 despite moderate correlation. Supplemental Figures S15D and S15F visually confirm that augmentation expands the dynamic range of predictions along the identity line, rather than artificially inflating correlation.

Relative to the PLS baseline (Figure S15E), the augmentation-enabled TabPFN (Figure S15F) improves by $\Delta R^2 \approx +0.14$ and $\Delta r \approx +0.10$. These incremental improvements are consistent with typical gains observed in other domains when measured on correlation or rank scales (e.g., Spearman ρ improving from 0.60 to 0.67, Figure S15C).

Importantly, these gains are not a byproduct of overfitting. Our LOOCV strategy strictly excludes all replicates of the held-out siRNA from the training set. Furthermore, Figure S15B demonstrates heterogeneous, per-siRNA error reductions—a pattern inconsistent with uniform overfitting. Instead, improvements are concentrated in high-leverage siRNAs where independent energy minimization replicates offer complementary structural features. Thus, in $n \ll p$ regimes, inference augmentation effectively leverages physically meaningful stochasticity to improve both calibration and rank accuracy.

To verify that this performance is robust to random initialization, we conducted two sets of independent control experiments (three runs each). The first set randomized the Rosetta energy minimization seeds while fixing the model training seed, whereas the second set fixed the Rosetta seed and randomized the training seeds. As Supplemental Figures S16A-F and S17A-F illustrate, the performance boost is highly stable across both scenarios. On average, the augmented TabPFN model significantly outperformed the baseline under randomized Rosetta seeds (paired t-test: $p = 0.0167$ for R^2, $p = 0.0201$ for Pearson r, and $p = 0.0418$ for Spearman ρ)and under randomized training seeds ($p = 0.0018$ for R^2, $p = 0.0009$ for Pearson r, and $p = 0.0028$ for Spearman ρ).

Finally, we systematically evaluated how the number of independent energy minimization replicates ($n\_repeats$) impacts Inference Augmentation. As shown in Figure S18, scaling $n\_repeats$ reveals a clear saturation point in performance. Under LOOCV, increasing $n\_repeats$ from 1 to 3 dramatically raises R^2 from 0.0902 to 0.5385, Pearson r from 0.3540 to 0.7365, and Spearman ρ from 0.3401 to 0.6678. The external test set follows a similar trend, with Pearson r jumping from 0.7262 to 0.8536. Beyond $n\_repeats = 3$, performance plateaus with only minor fluctuations (e.g., at $n\_repeats = 9$, external test set Pearson $r = 0.8679$). Consequently, $n\_repeats = 3$ offers an optimal balance between computational efficiency and predictive accuracy.

Supplemental Figures

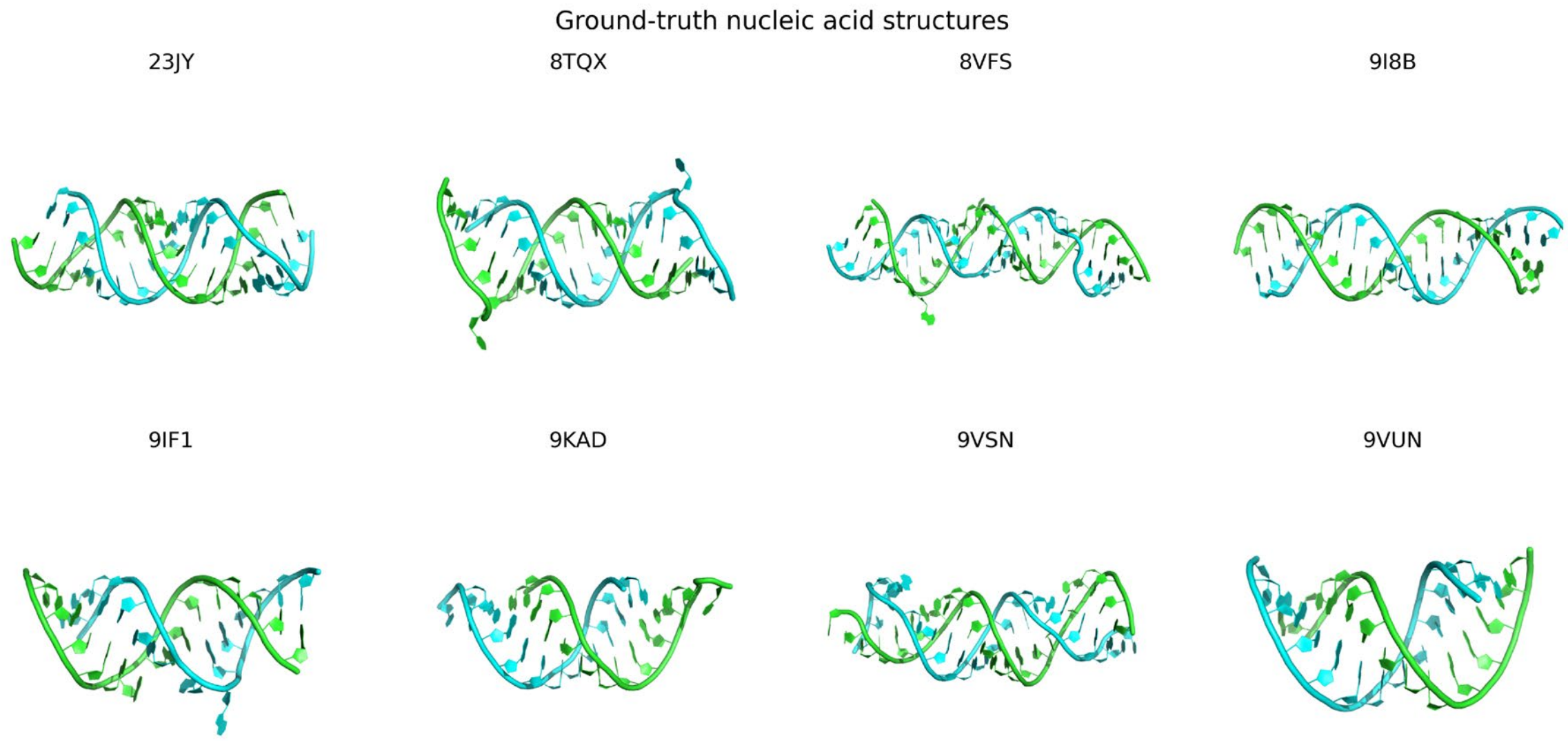

Figure S1. Visualization of structures in the double-stranded nucleic acids benchmark, based on PDB structures released after January 1, 2024.

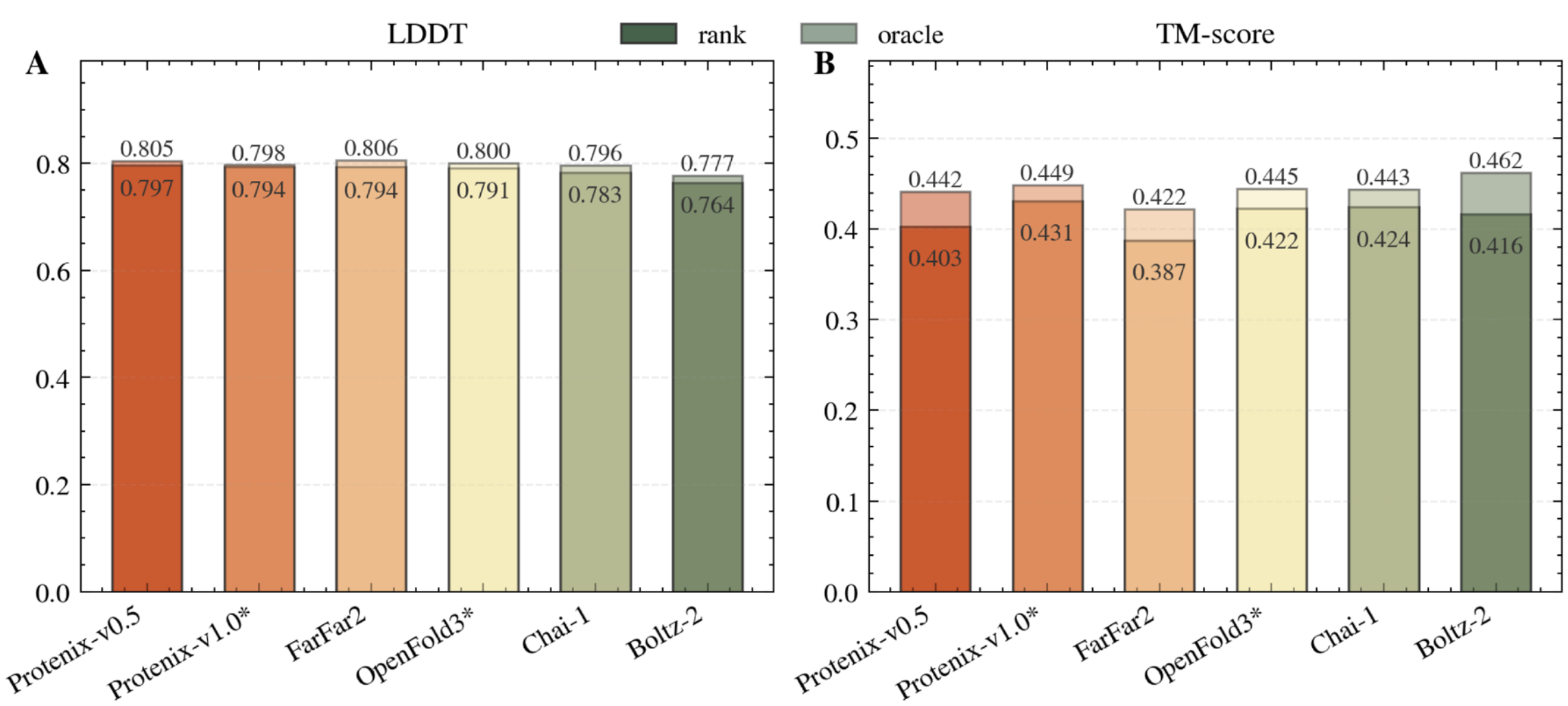

Figure S2. Performance of structure prediction on double-stranded nucleic acids. Bar plots show the average (A) LDDT and (B) TM-score of the predictions, calculated as FoldBench.[3] For each structure, 5 samples were generated; metrics are reported based on the top-ranked sample by the default ranking metric ('rank') and the best possible sample ('oracle'). Asterisks (*) denote the latest released methods included as a complement during the review process.

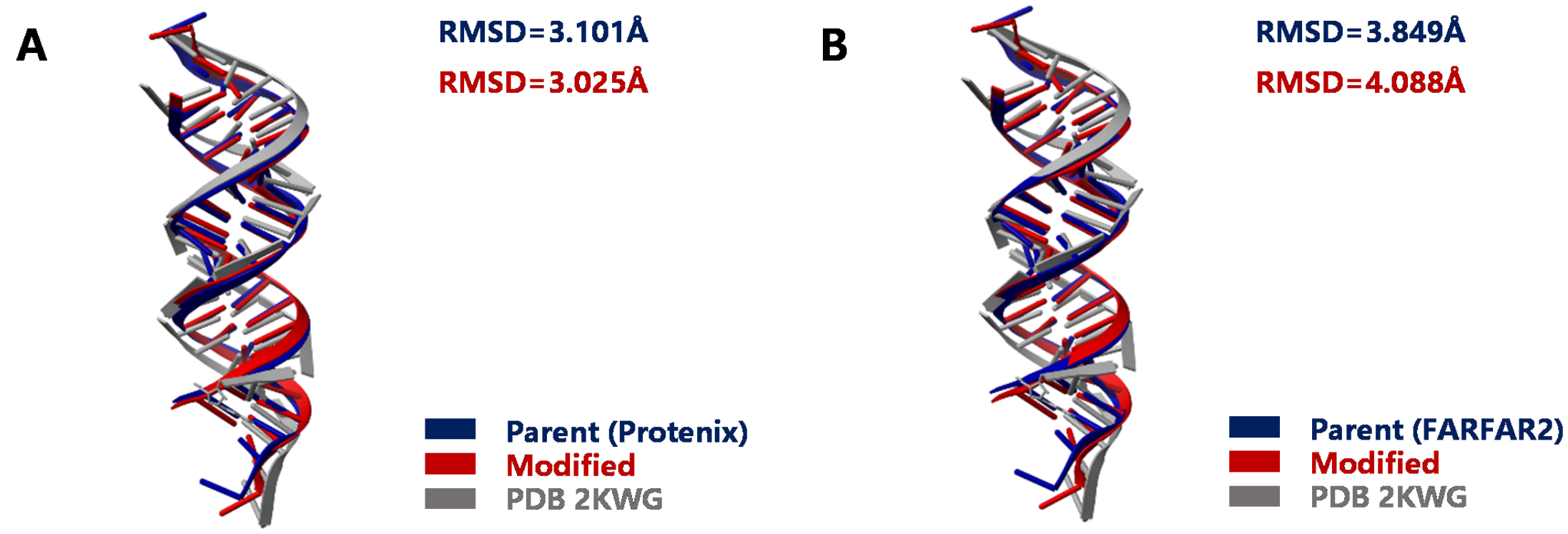

Figure S3. Comparison of the two-step modified siRNA structure prediction pipeline using Protenix versus FARFAR2 as the initial structure prediction tool, evaluated on PDB entry 2KWG (a fully chemically modified siRNA duplex). *(A) Ribbon superimposition of the PDB 2KWG reference structure (grey), the unmodified siRNA predicted by Protenix (blue), and the Rosetta-refined modified siRNA (red). Backbone RMSDs relative to 2KWG are 3.101 Å and 3.025 Å, respectively. (B) As in (A), but using FARFAR2 for initial structure prediction (blue), with backbone RMSDs of 3.849 Å and 4.088 Å, respectively. Lower RMSD values indicate better agreement with the experimental reference structure.*

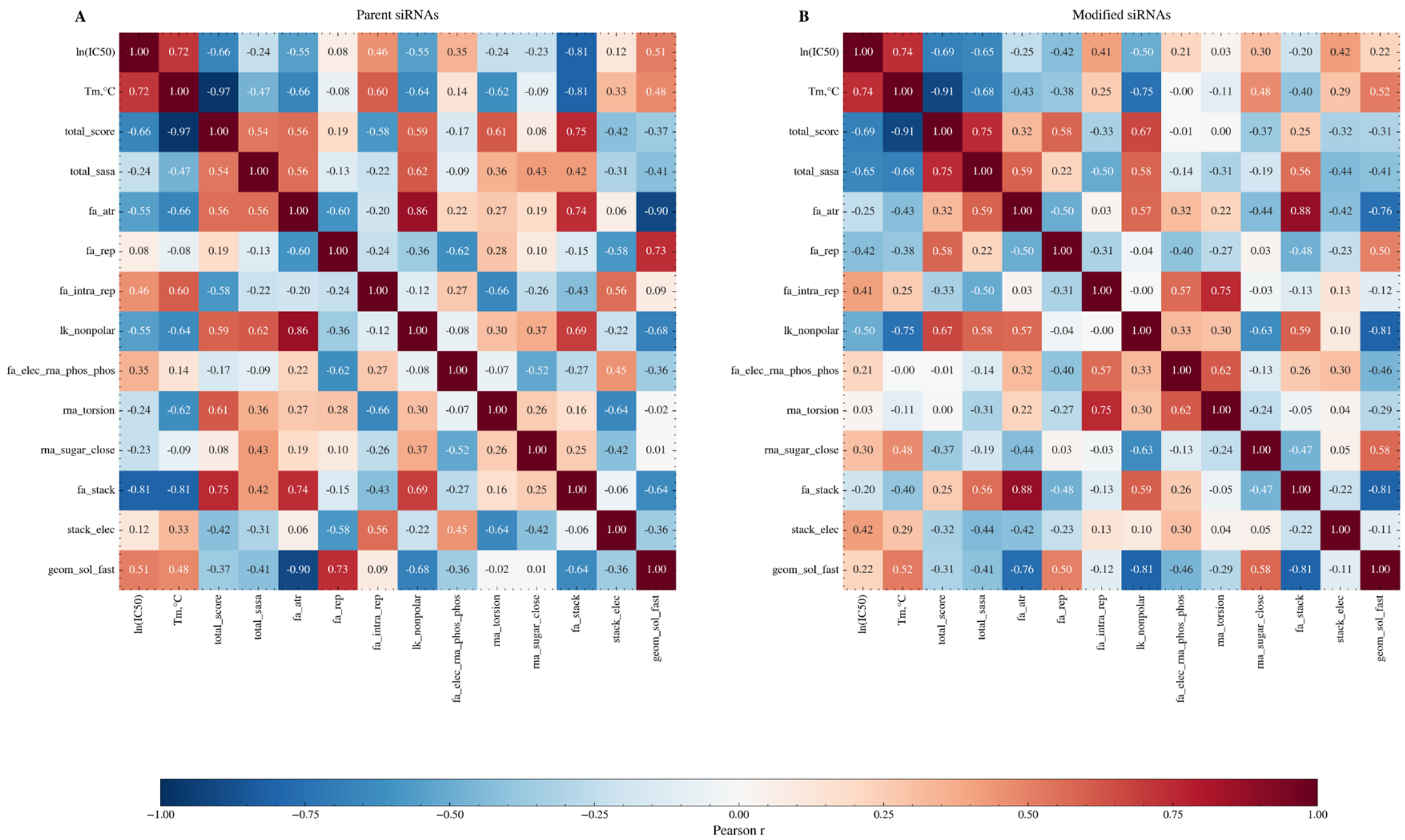

Figure S4. Pearson correlation heatmaps among Rosetta energy terms, $log_e(IC_{50})$, and melting temperature (T_m). *Colour intensity reflects the magnitude of r; red and blue denote positive and negative correlations, respectively. (A) Correlation matrix for parent (unmodified) siRNAs, highlighting the strong negative correlation between total energy score and both T_m and $log_e(IC_{50})$. (B) Correlation matrix for chemically modified siRNAs, showing analogous relationships between total energy score, T_m, and $log_e(IC_{50})$.*

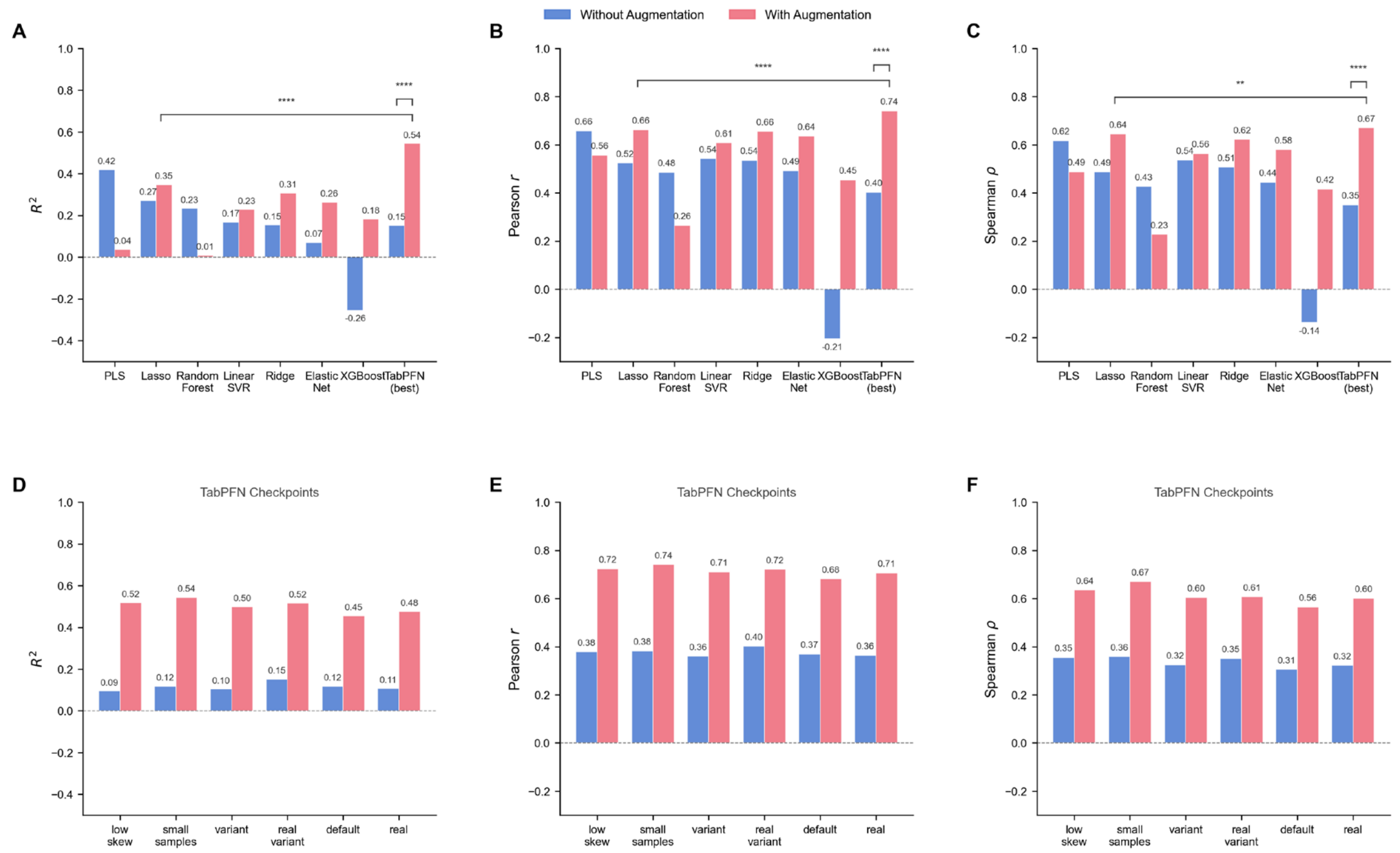

Figure S5. Leave-one-out cross-validation performance on the multi-replicate test set, in which the three energy-calculation replicates of each held-out siRNA are averaged into a single test sample, placing greater emphasis on prediction robustness. *(A–C) Bar charts showing* R^2*, Pearson* r*, and Spearman* ρ*, respectively, for all evaluated models without Inference Augmentation (blue) and with Inference Augmentation (red). "TabPFN (best)" refers to the highest-performing checkpoint. (D–F) Bar charts showing* R^2*, Pearson* r*, and Spearman* ρ*, respectively, for individual TabPFN checkpoints without augmentation (blue) and with Inference Augmentation (red). Significance was assessed by Steiger's Z-test: '****' indicates* $p < 0.001$*; '***'* $p < 0.005$*; '**'* $p < 0.01$*; '*'* $p < 0.05$*.*

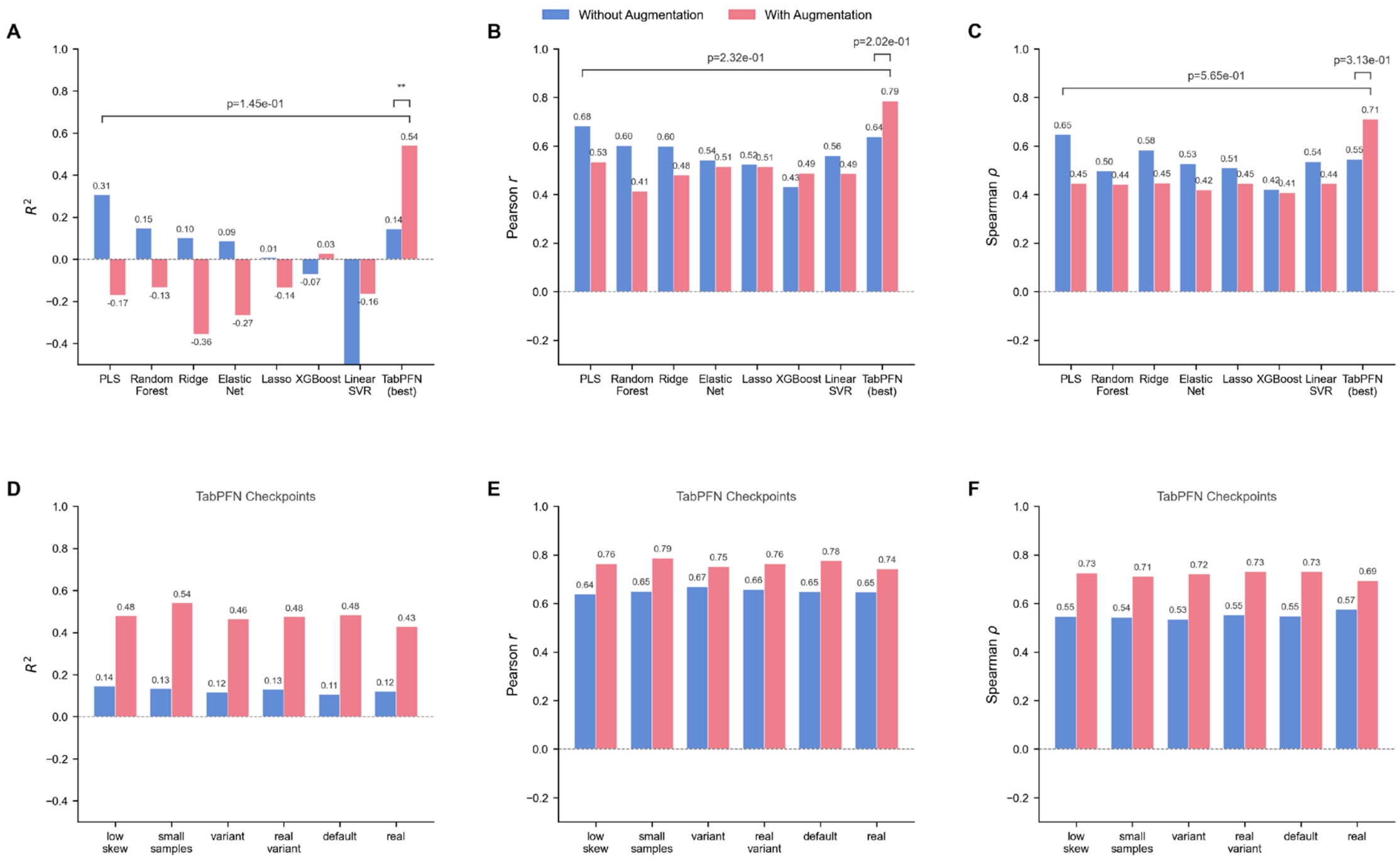

Figure S6. Five-fold cross-validation performance on the multi-replicate test set, in which multiple energy-calculation replicates of the same siRNA may appear within the test set, placing greater demands on model robustness. *(A–C) Bar charts showing R^2, Pearson r, and Spearman ρ, respectively, for all evaluated models without Inference Augmentation (blue) and with Inference Augmentation (red). "TabPFN (best)" refers to the highest-performing checkpoint. (D–F) Bar charts showing R^2, Pearson r, and Spearman ρ, respectively, for individual TabPFN checkpoints without augmentation (blue) and with Inference Augmentation (red). Significance was assessed by paired t-test: '****' indicates $p < 0.001$; '***' $p < 0.005$; '**' $p < 0.01$; '*' $p < 0.05$.*

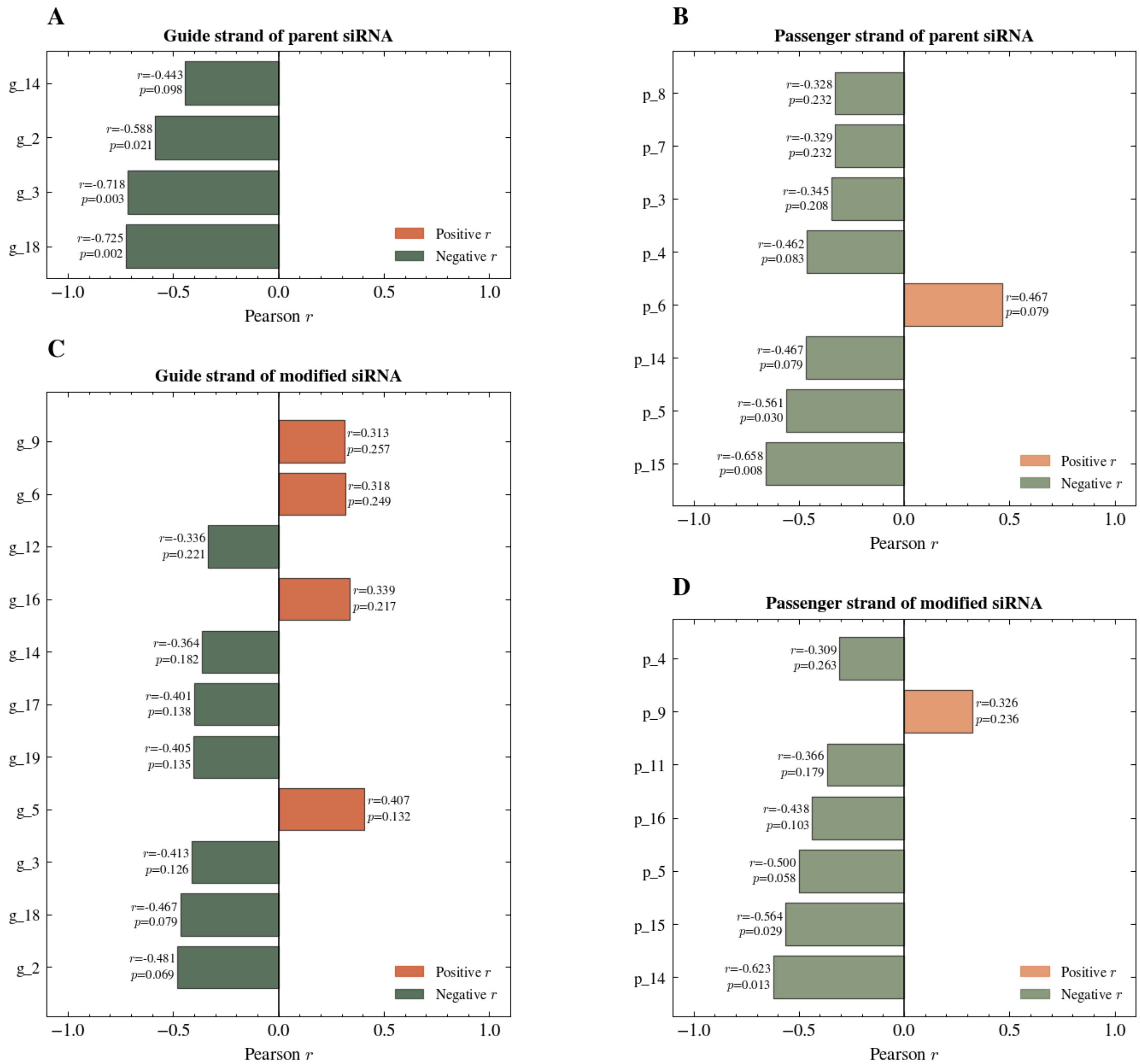

Figure S7. Position-specific Pearson correlation between the per-residue Rosetta energy (ΔE) and log_{10}(IC$_{50}$) for parent (unmodified) and chemically modified siRNAs, shown separately for the guide strand and the passenger strand.

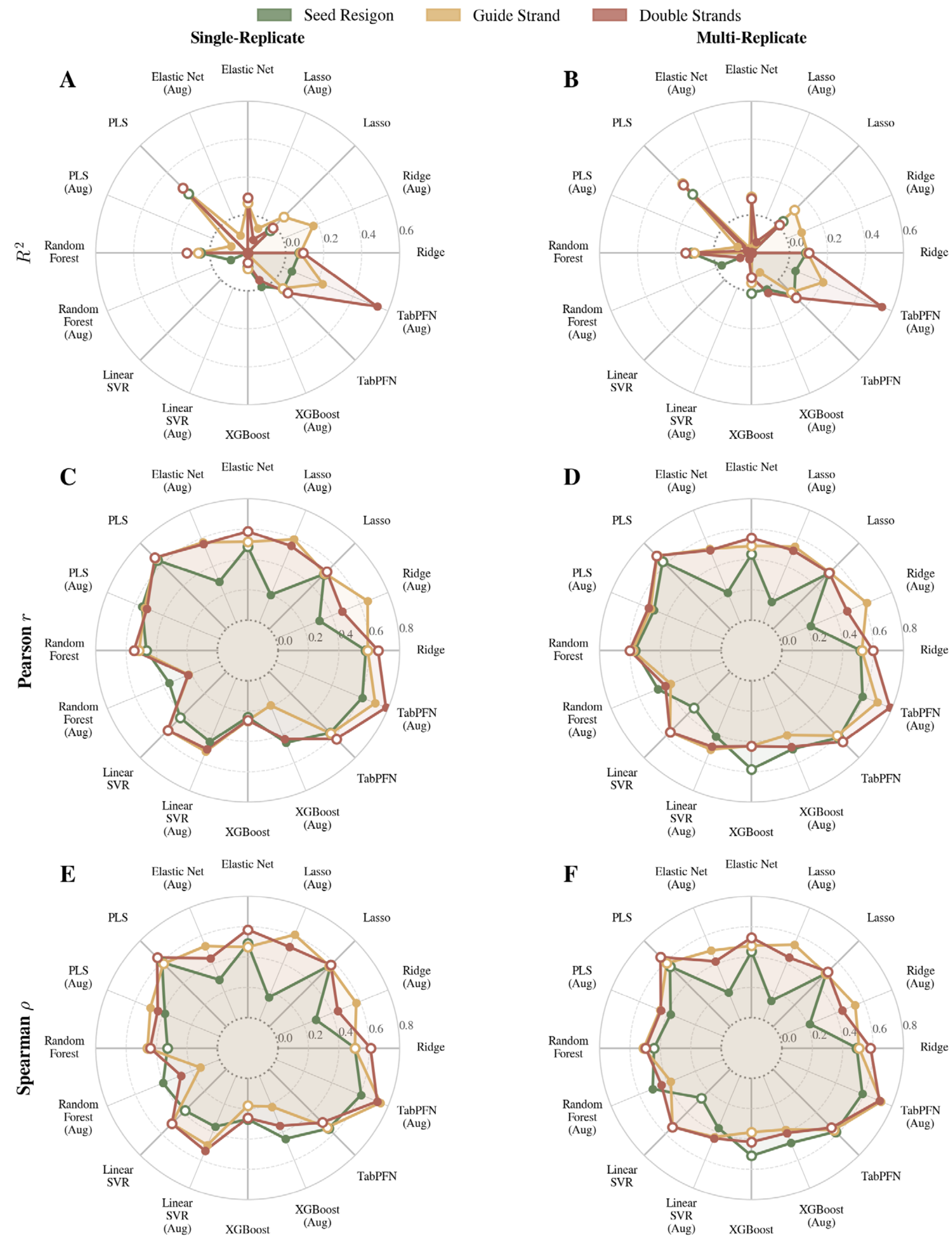

Figure S8. Radar chart comparison of predictive performance for different positional energy feature subsets in stratified 5-fold cross-validation, complementing the leave-one-out ablation analysis (Figure 6). *The evaluation metrics are R^2 (A, B), Pearson r (C, D), and Spearman ρ (E, F). Panels (A, C, E) show performance on the single-replicate test set, while panels (B, D, F) display performance on the multi-replicate test set. Three feature configurations are compared: full dual-strand features (guide- and passenger-strand features; red), guide-strand-only features (yellow), and seed-region-only features (green). Each axis corresponds to an evaluated machine learning or tabular deep learning model; a larger enclosed area indicates superior generalization across models. Solid markers represent models with Inference Augmentation; hollow markers represent models without augmentation.*

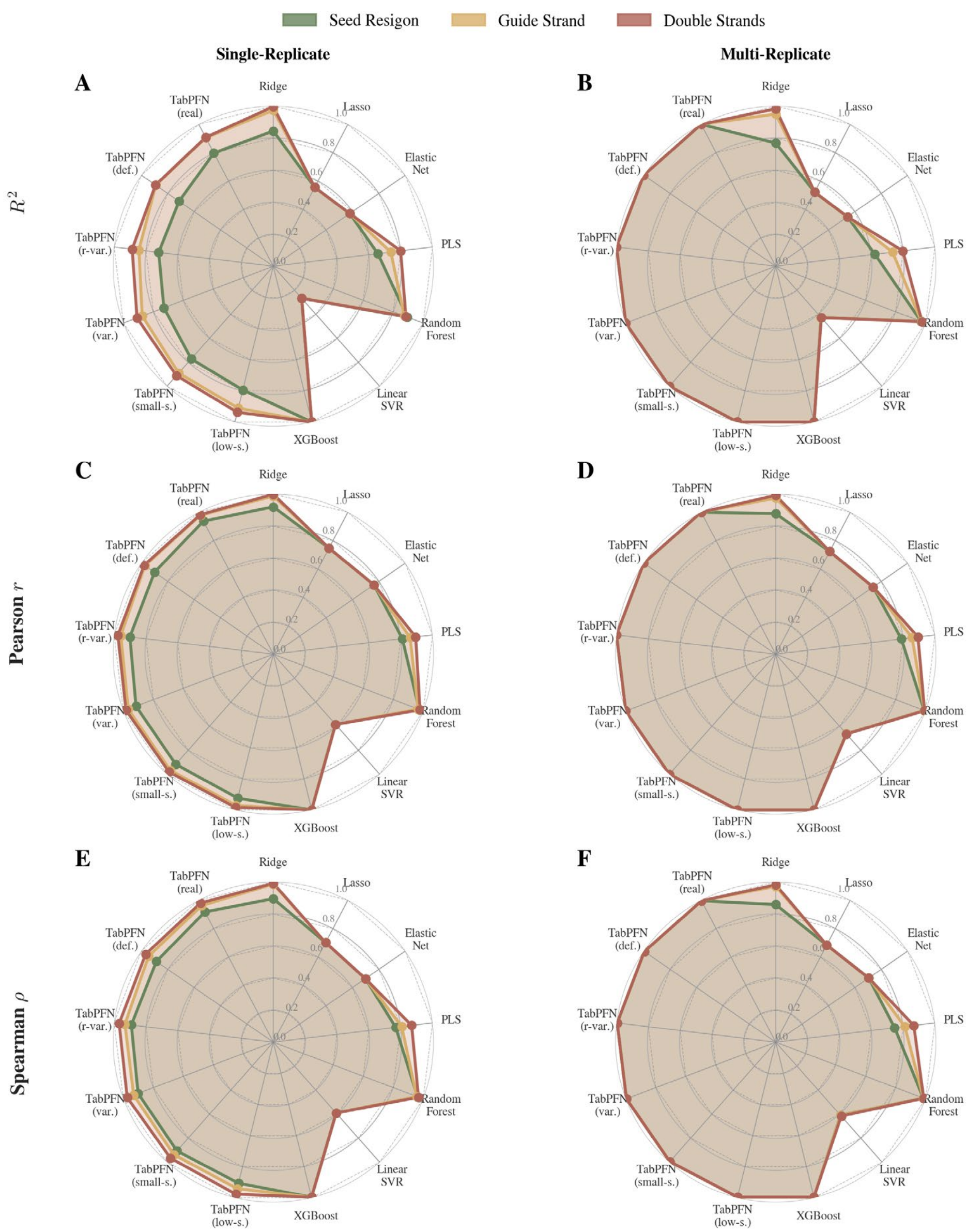

Figure S9. Radar chart comparison of predictive performance for different positional energy feature subsets under full-dataset fitting (training and evaluation on the complete dataset without a held-out test set). *The evaluation metrics are R^2 (A, B), Pearson r (C, D), and Spearman ρ (E, F). Panels (A, C, E) show performance on the single-replicate dataset, while panels (B, D, F) display performance on the multi-replicate dataset. Three feature configurations are compared: full dual-strand features (guide- and passenger-strand features; red), guide-strand-only features (yellow), and seed-region-only features (green). Each axis corresponds to an evaluated machine learning or tabular deep learning model; a larger enclosed area indicates superior predictive performance. Solid markers represent models trained with Inference Augmentation, whereas hollow markers designate models evaluated without Inference Augmentation.*

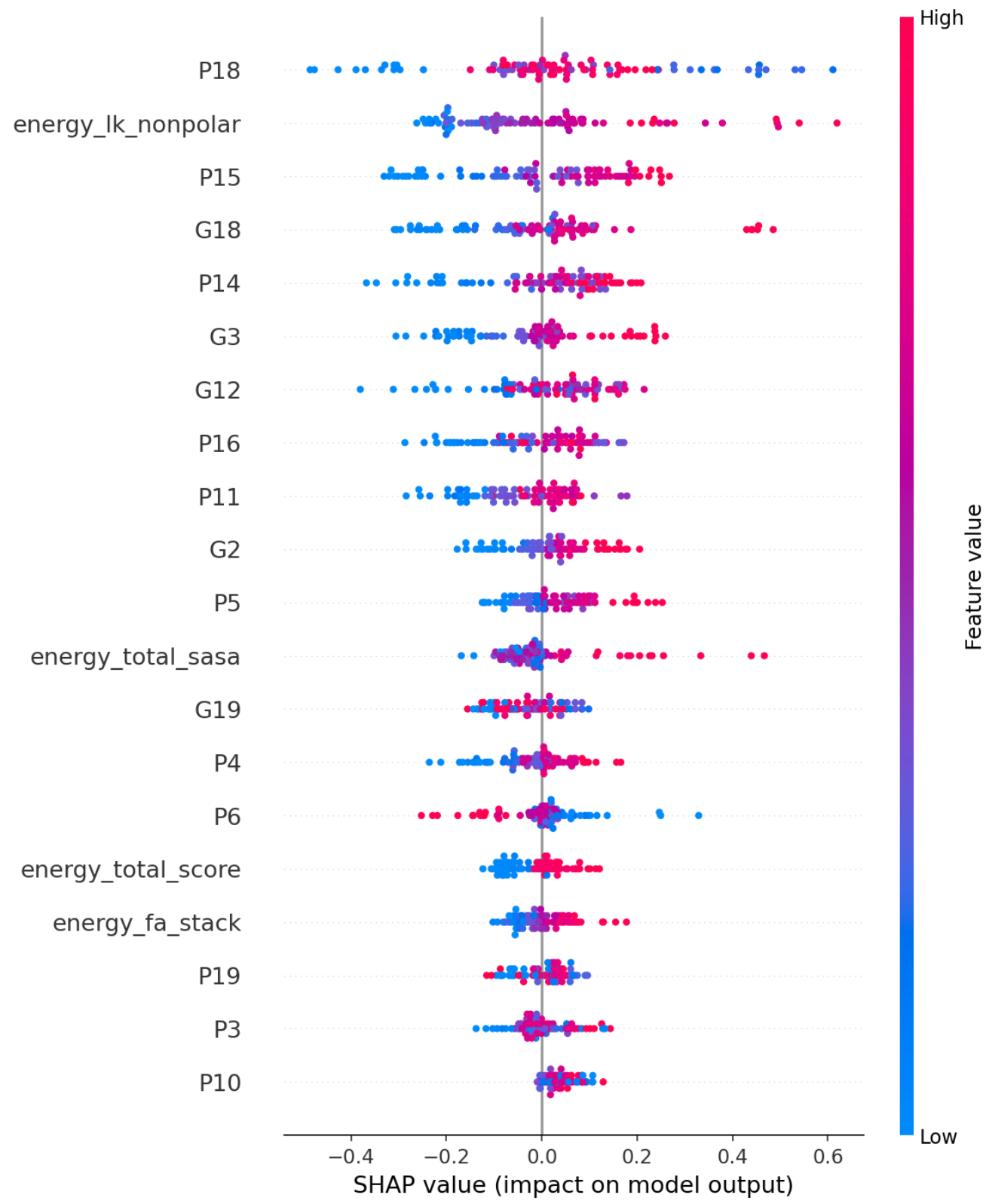

Figure S10. SHAP beeswarm summary plot showing the contribution of the top 20 Rosetta energy features to the Inference-Augmented TabPFN model's predictions of $-log_{10}(\mathrm{IC}_{50})$. *Features are ranked by mean absolute SHAP value (top to bottom); each point represents a single sample, coloured by relative feature value (red: high, blue: low).*

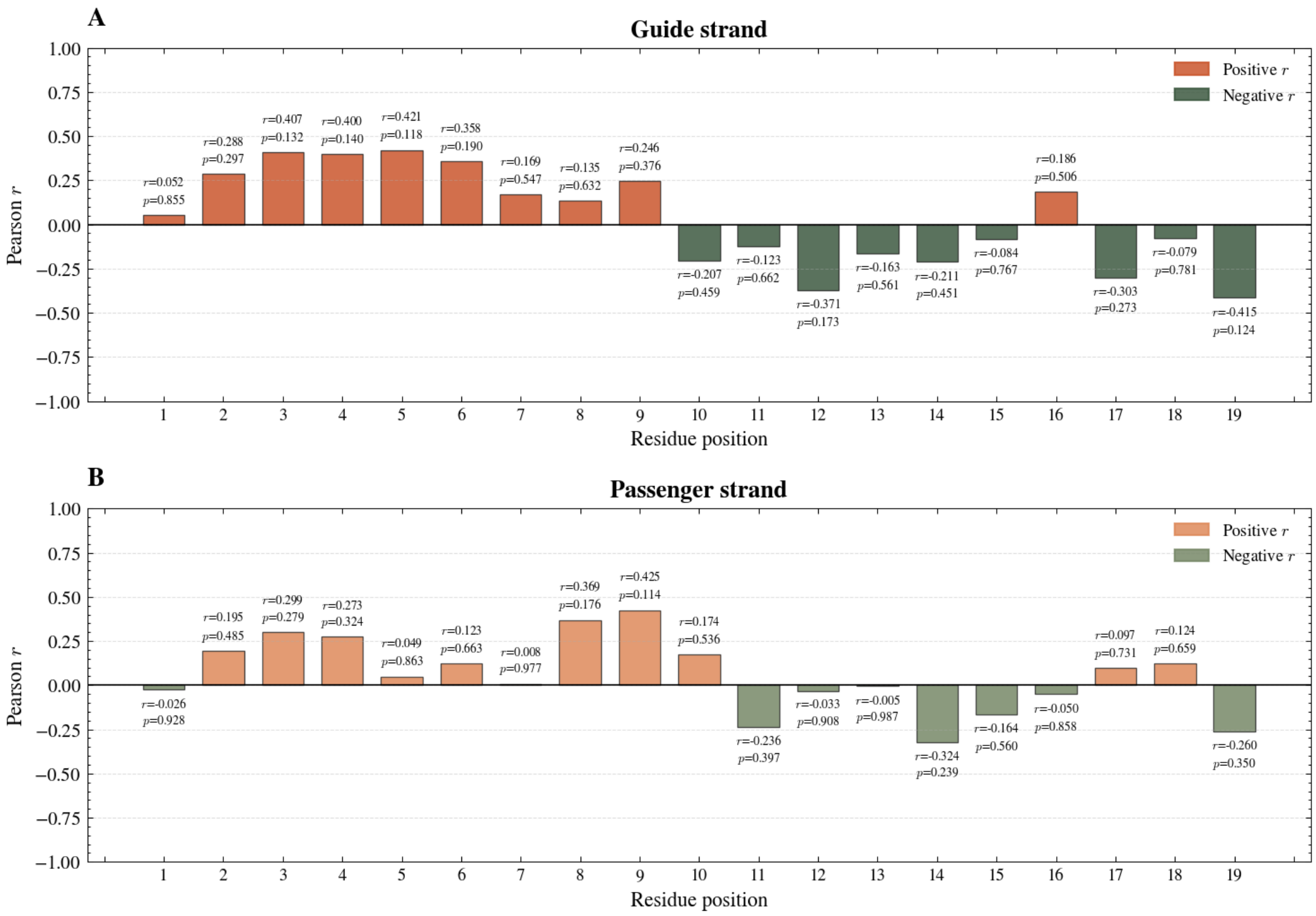

Figure S11. Position-specific Pearson correlation between the modification-induced Rosetta energy change ($\Delta\Delta E = \Delta E_{mod} - \Delta E_{unmod}$) and log_{10}(IC$_{50}$) across all nucleotide positions, shown separately for parent and modified siRNAs and for both the guide strand and the passenger strand.

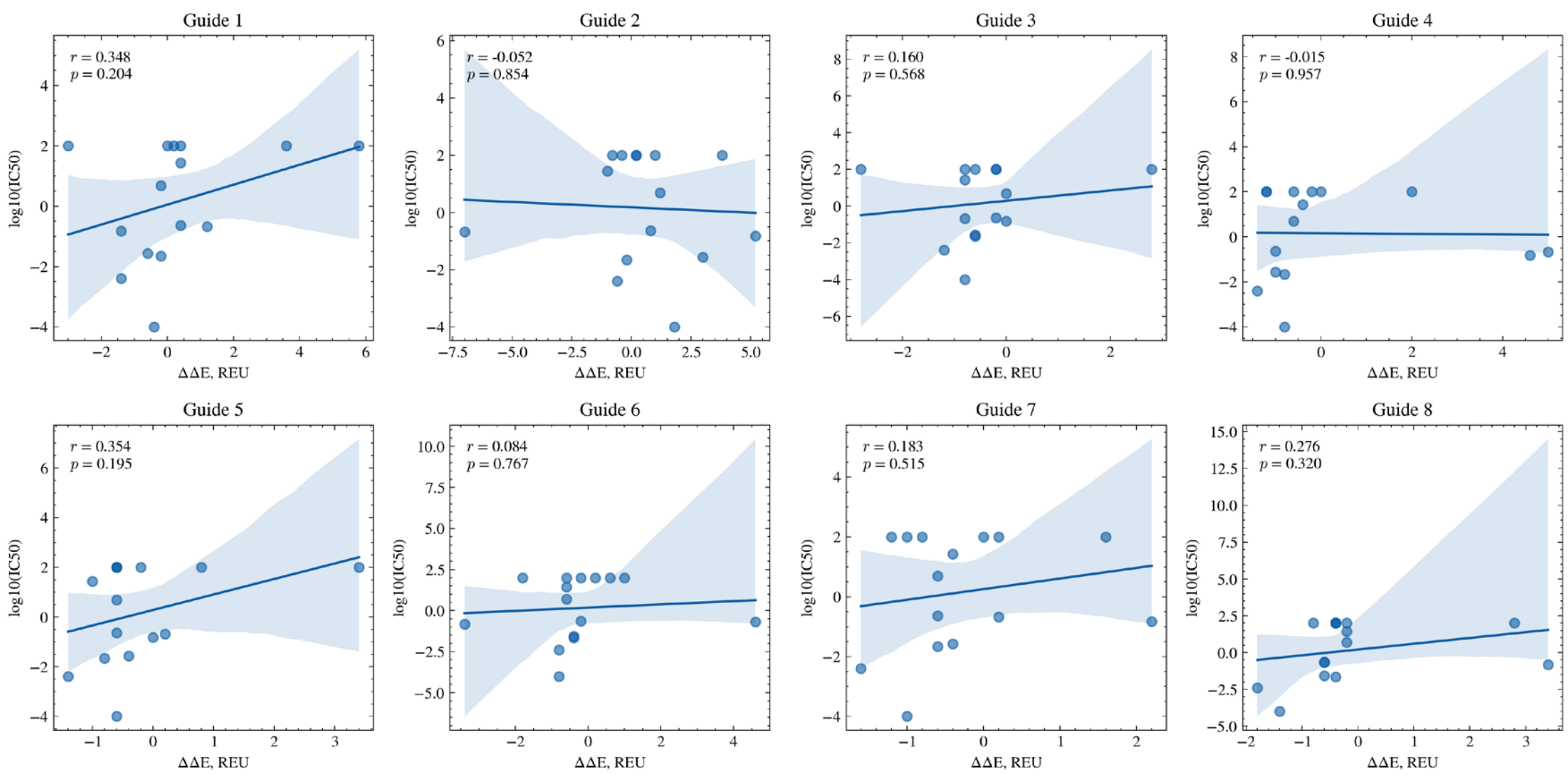

Figure S12 Pearson correlation between molecular dynamics (MD)-derived modification-induced energy changes ($\Delta\Delta E_{MD}$) and $log_{10}(\text{IC}_{50})$ at seed-region positions (g2–g8 of the guide strand), reproduced from Kliuchnikov et al.[4]

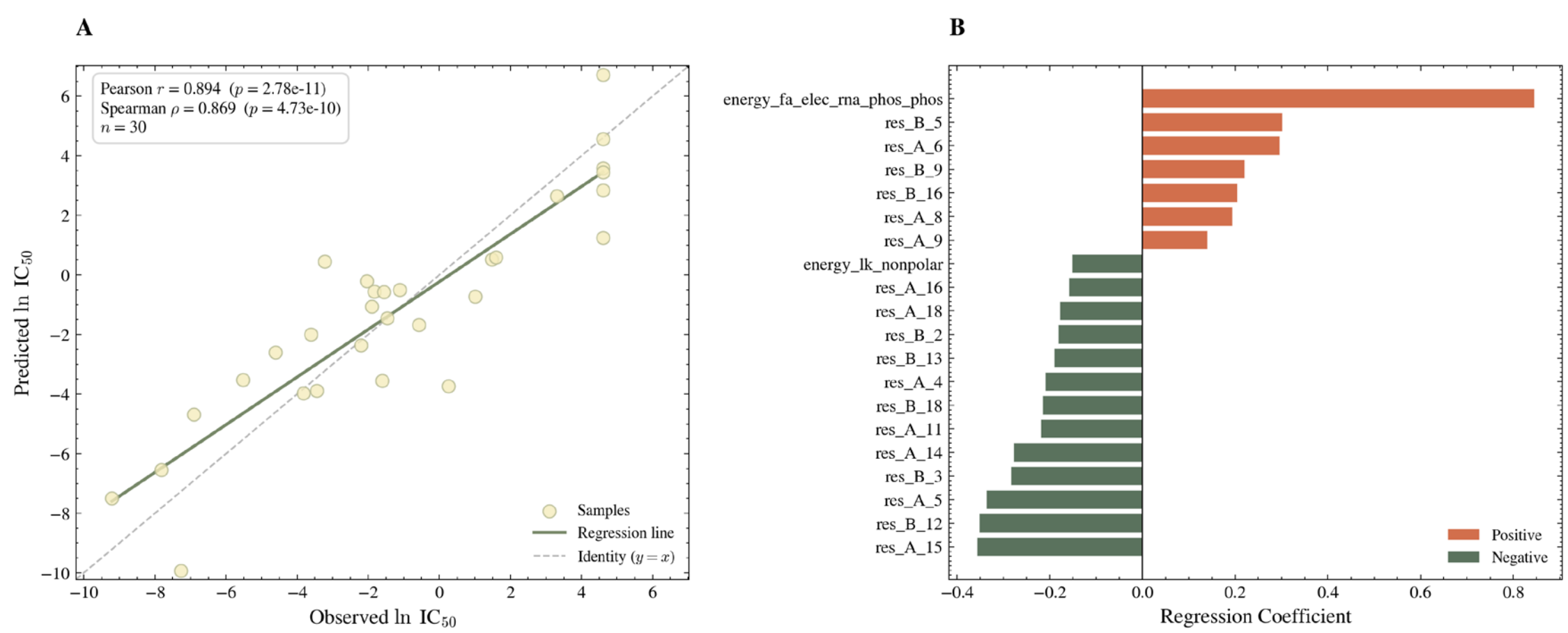

Figure S13. Performance and variable importance of the PLS model trained on Rosetta energy features using the full dataset (no held-out test set). (A) Scatter plot of predicted versus experimental $log_e(\text{IC}_{50})$, with Pearson $r = 0.894$ ($p = 2.8 \times 10^{-11}$) and Spearman ρ indicated; the dashed line represents perfect agreement ($y = x$). This surpasses the MD-derived PLS baseline ($r = 0.87$) from Kliuchnikov et al.[4] (B) Variable Importance in Projection (VIP) scores for the 20 most influential features of the PLS model, indicating the dominant contribution of whole-duplex energy terms and selected per-residue positions.

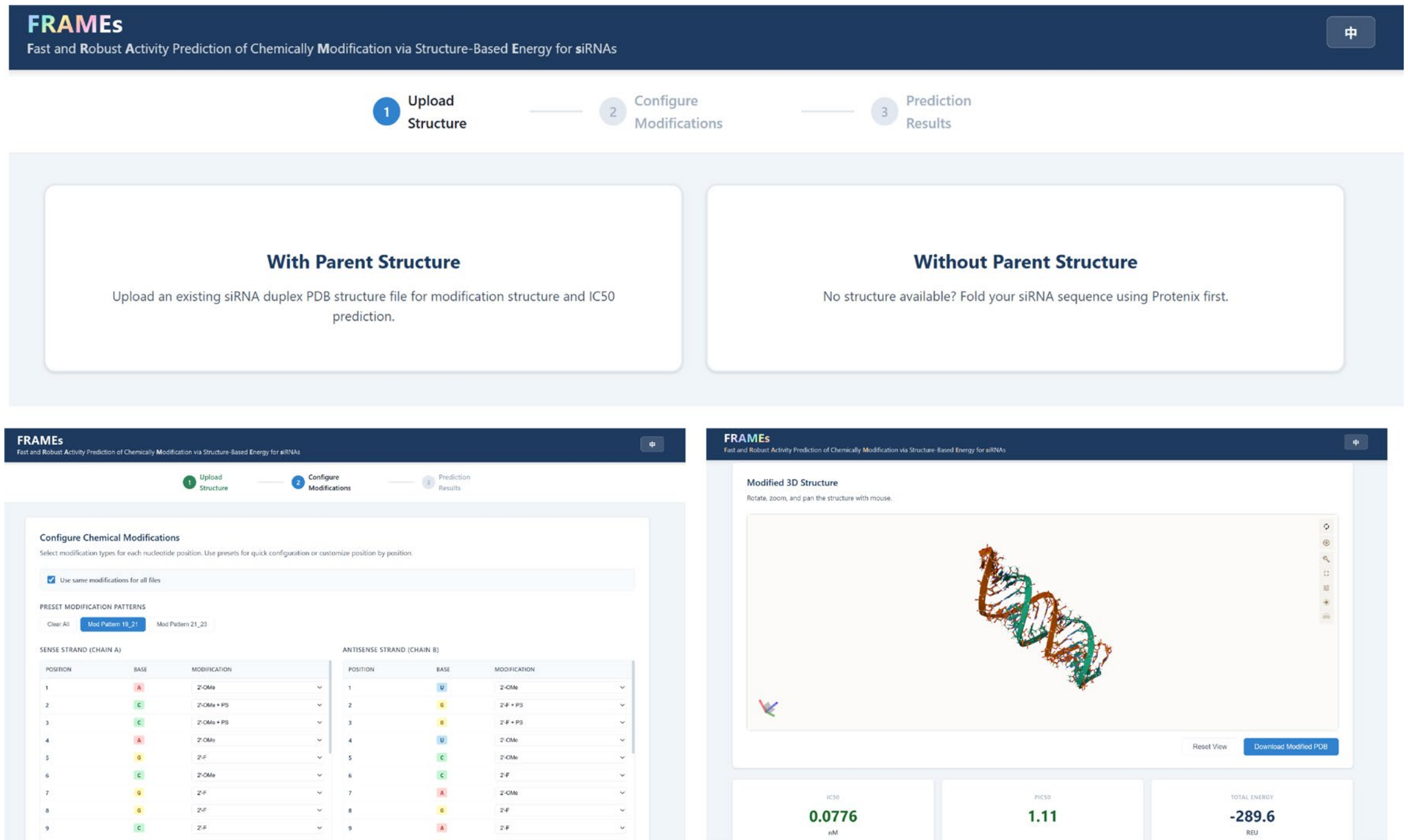

Figure S14. Screenshot of the FRAMEs web server. *The input panel (upper) accepts a PDB file of the unmodified siRNA structure and a strand-specific modification pattern (lower left). Upon submission, the results panel (lower right) displays the predicted pIC_{50}, the corresponding IC_{50} estimate, and the Rosetta total energy of the refined duplex.*

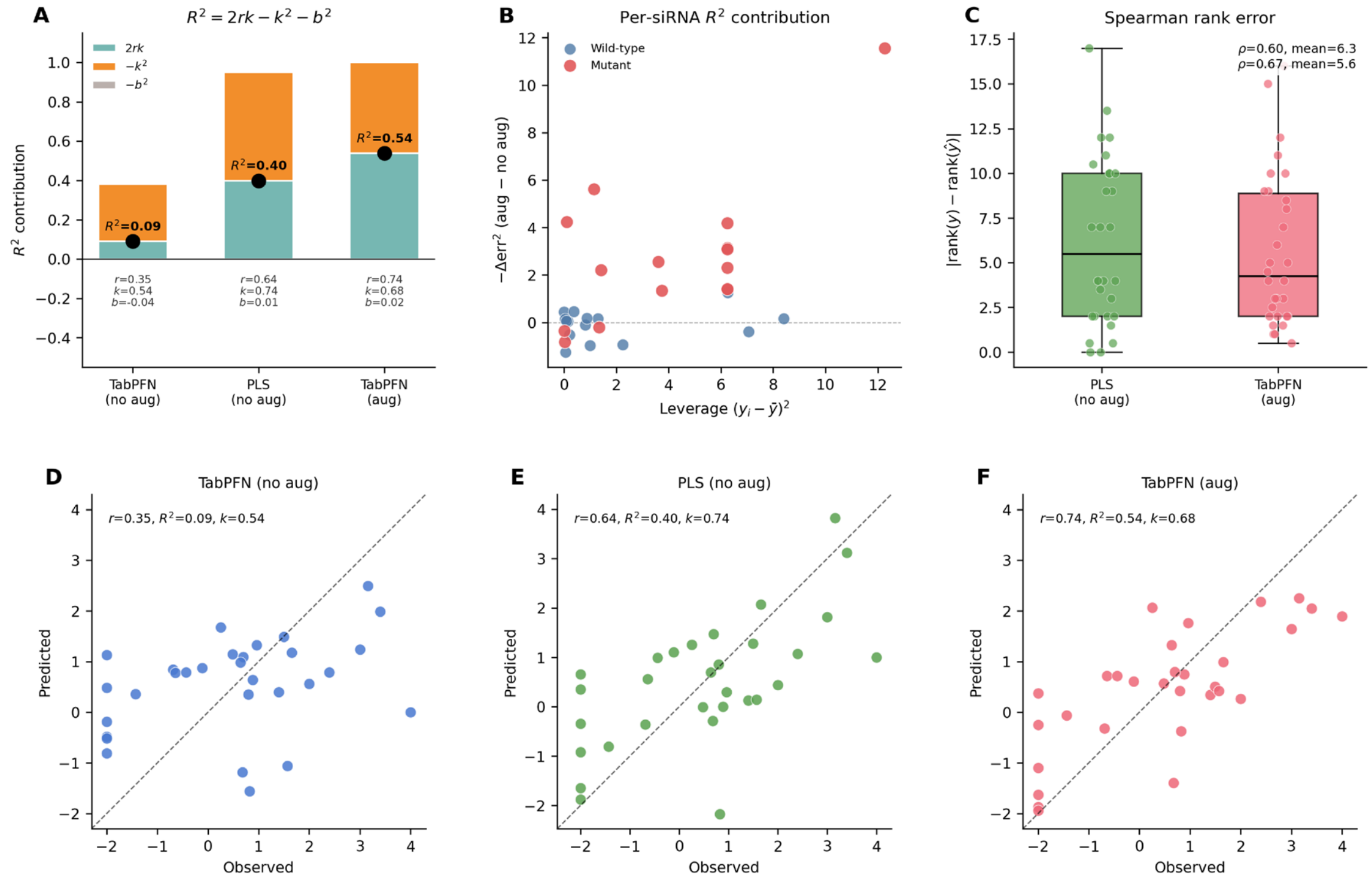

Figure S15. Attribution analysis of inference augmentation performance under leave-one-out cross-validation. (A) Decomposition of the coefficient of determination. (B) Per-siRNA contribution to R^2 improvement. (C) Spearman rank error distribution. (D) Predicted vs. observed (TabPFN, no augmentation). (D) Predicted vs. observed (PLS, no augmentation). (D) Predicted vs. observed (TabPFN, with augmentation).

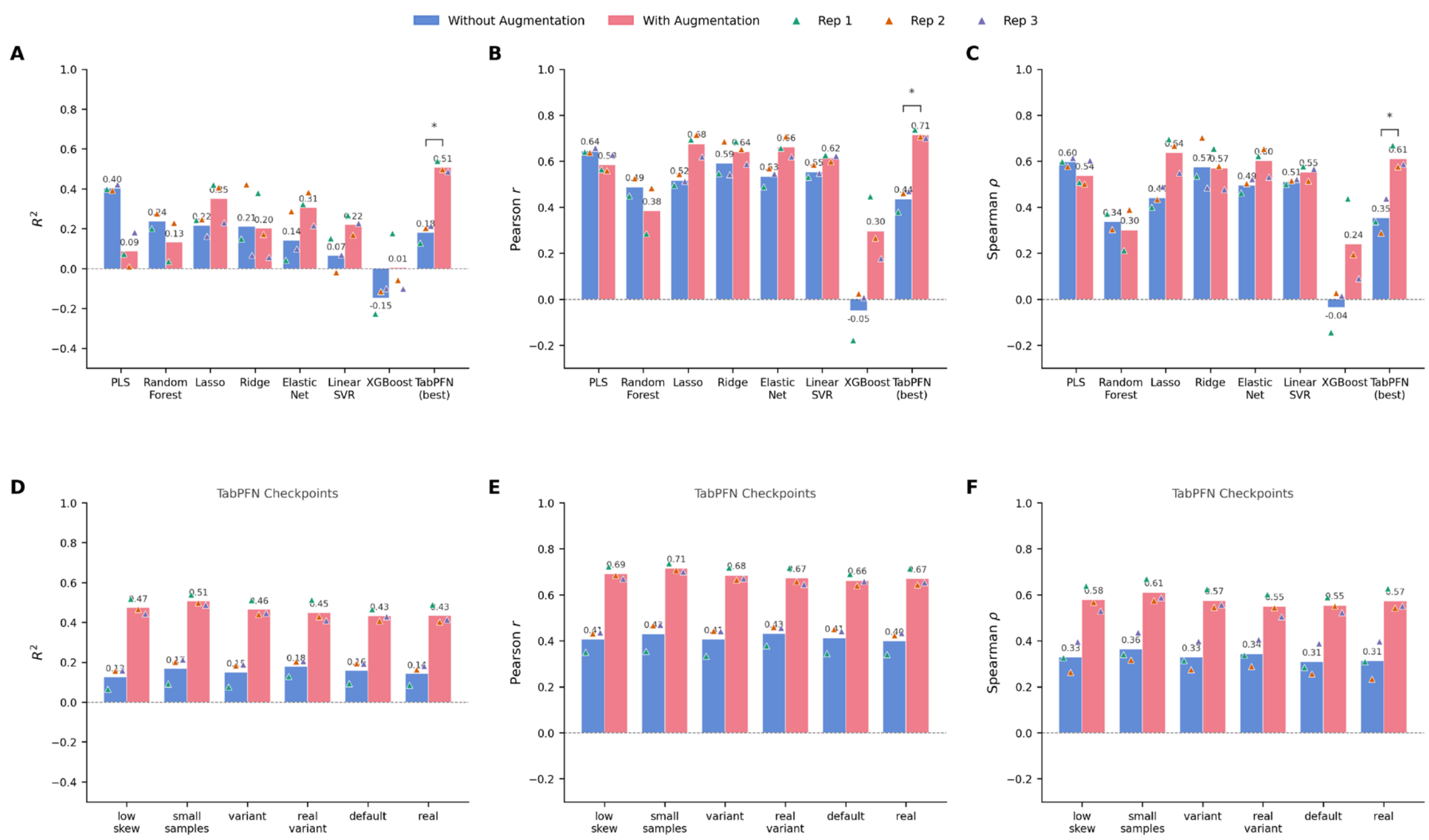

Figure S16. Leave-one-out cross-validation performance across three independent experimental repeats with randomized Rosetta energy minimization seeds. *(A–C) Bar charts showing* R^2*, Pearson* r*, and Spearman* ρ*, respectively, for all evaluated models without Inference Augmentation (blue) and with Inference Augmentation (red). "TabPFN (best)" refers to the highest-performing checkpoint. (D–F) Bar charts showing* R^2*, Pearson* r*, and Spearman* ρ*, respectively, for individual TabPFN checkpoints without augmentation (blue) and with Inference Augmentation (red). Significance was assessed by paired two-sided* t*-test: '*'* $p < 0.05$.

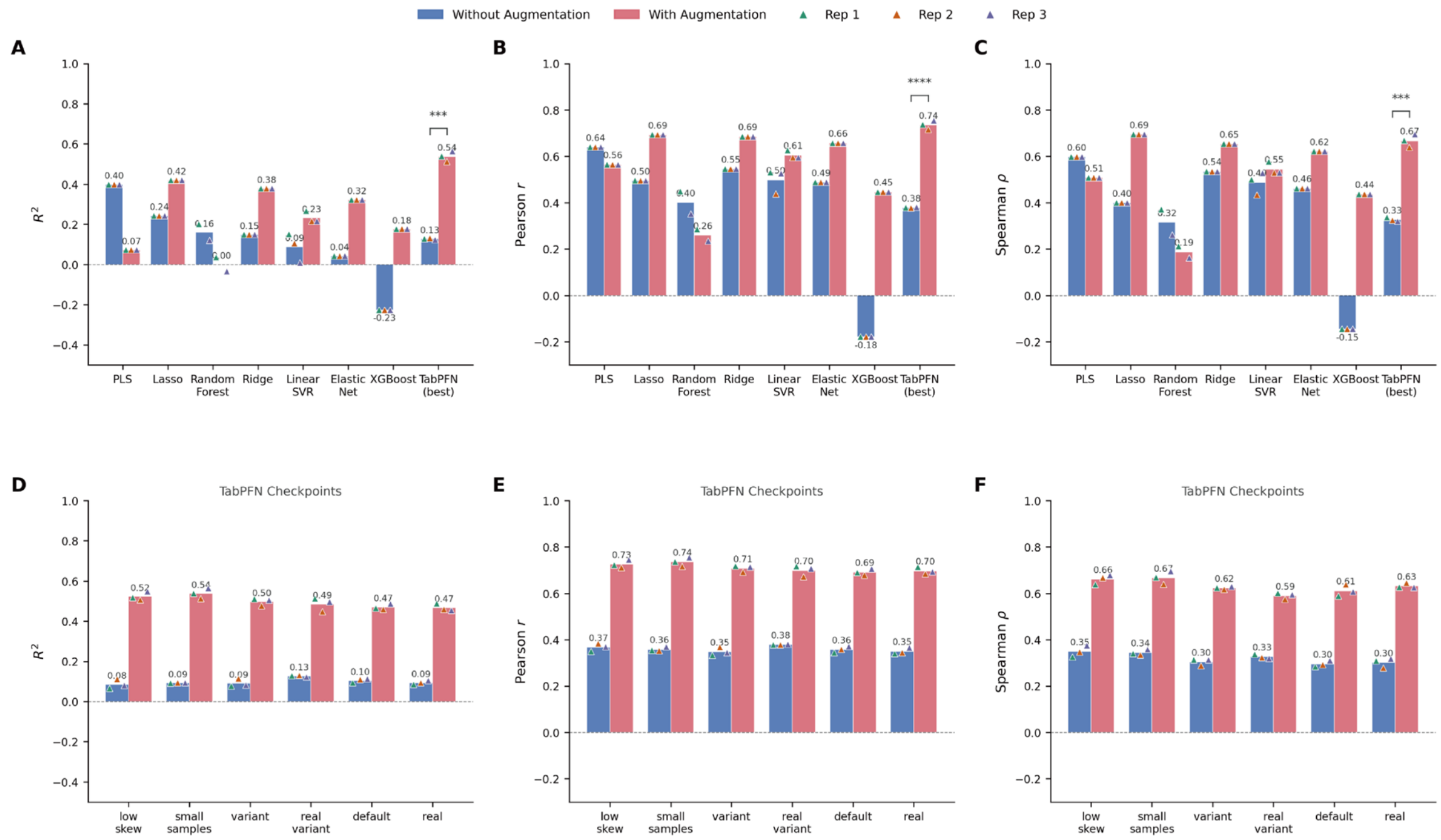

Figure S17. Leave-one-out cross-validation performance across three independent experimental repeats with randomized model training seeds. *(A–C) Bar charts showing R^2, Pearson r, and Spearman ρ, respectively, for all evaluated models without Inference Augmentation (blue) and with Inference Augmentation (red). "TabPFN (best)" refers to the highest-performing checkpoint. (D–F) Bar charts showing R^2, Pearson r, and Spearman ρ, respectively, for individual TabPFN checkpoints without augmentation (blue) and with Inference Augmentation (red). Significance was assessed by paired two-sided t-test: '****' indicates $p < 0.001$; '***' $p < 0.005$; '**' $p < 0.01$; '*' $p < 0.05$.*

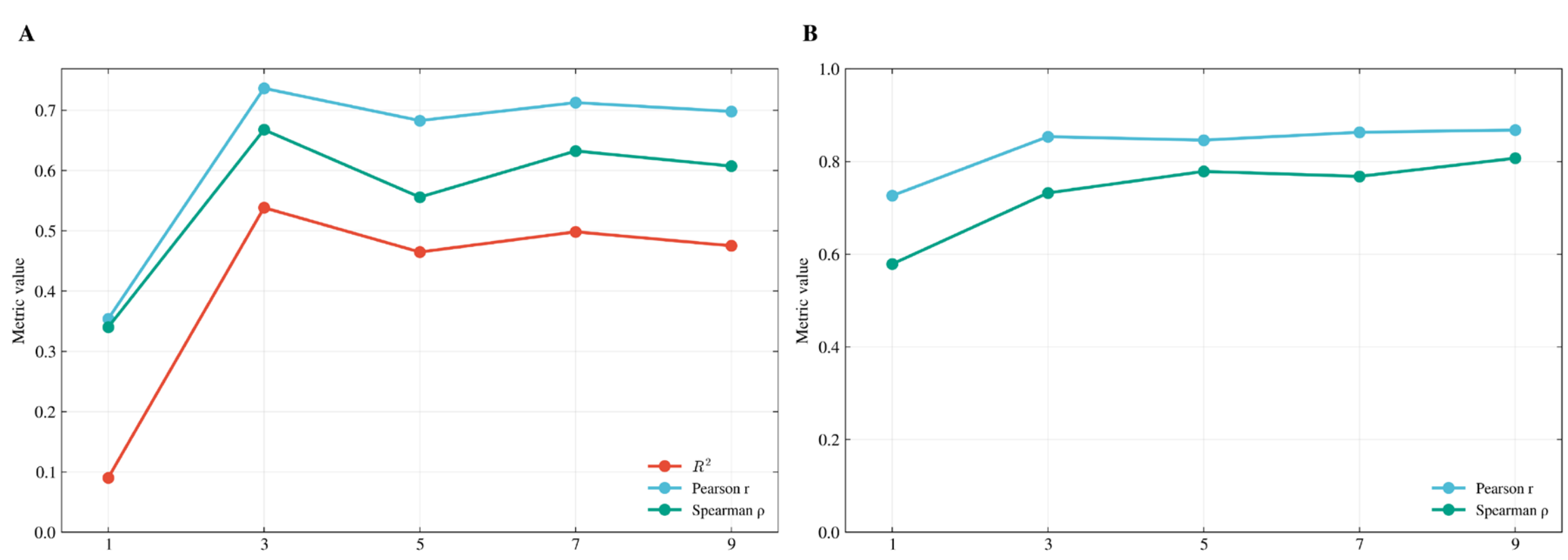

Figure S18. Effect of training repeat augmentation on siRNA activity prediction performance. *(A) Leave-one-out cross-validation performance. (B) External test set performance.*

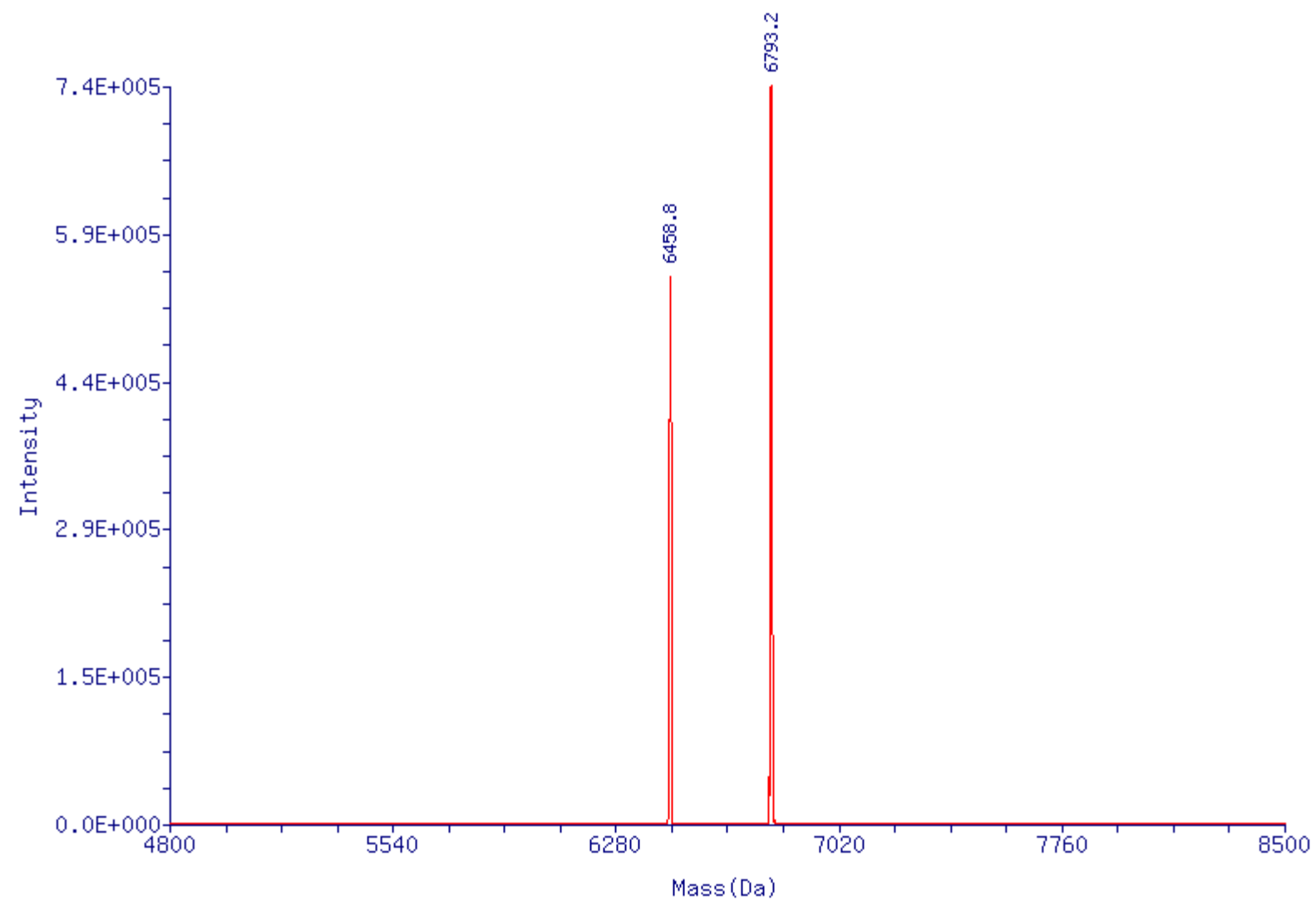

Figure S19. Mass spectrum of CDC20-1.

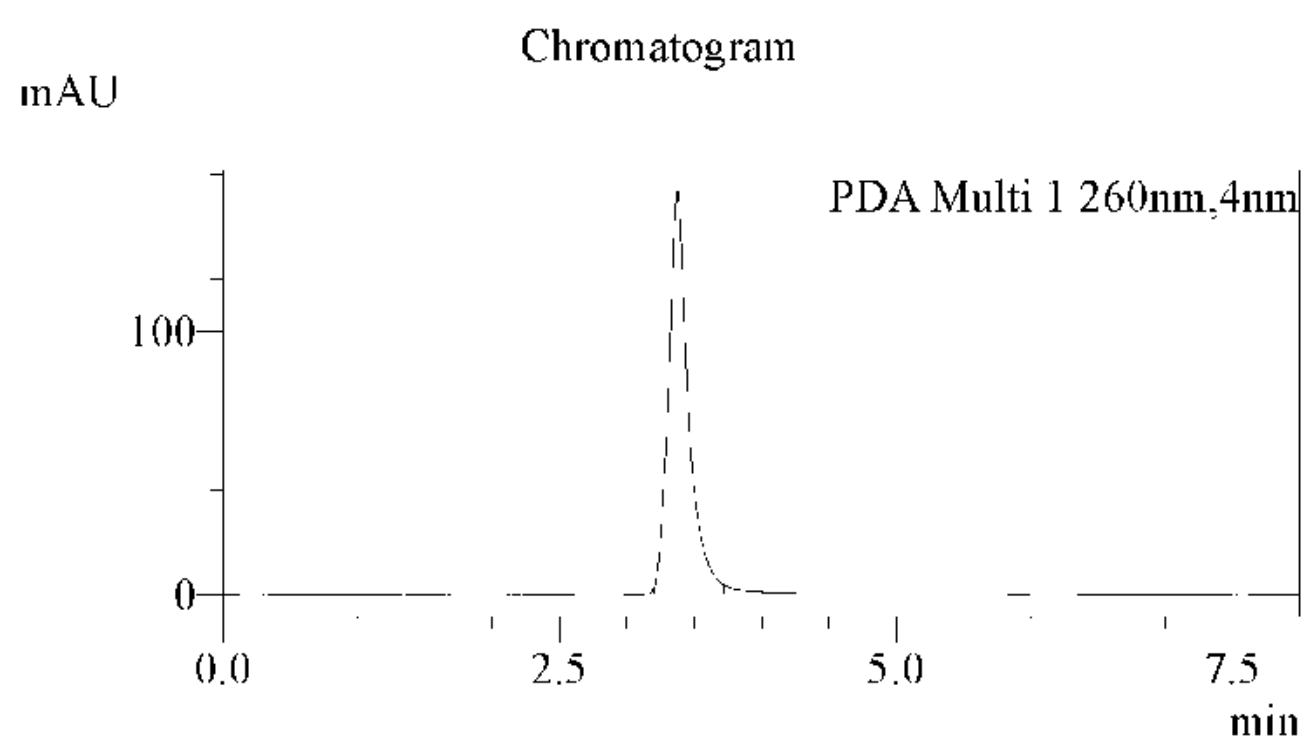

Peak Table

PDA Ch1 260nm

Peak#	Ret. Time	Area	Height	Area%
1	3.195	620	934	0.039
2	3.376	1563496	152864	98.209
3	3.739	27889	2970	1.752
Total		1592005	156768	100.000

Figure S20. HPLC chromatogram of CDC20-1.

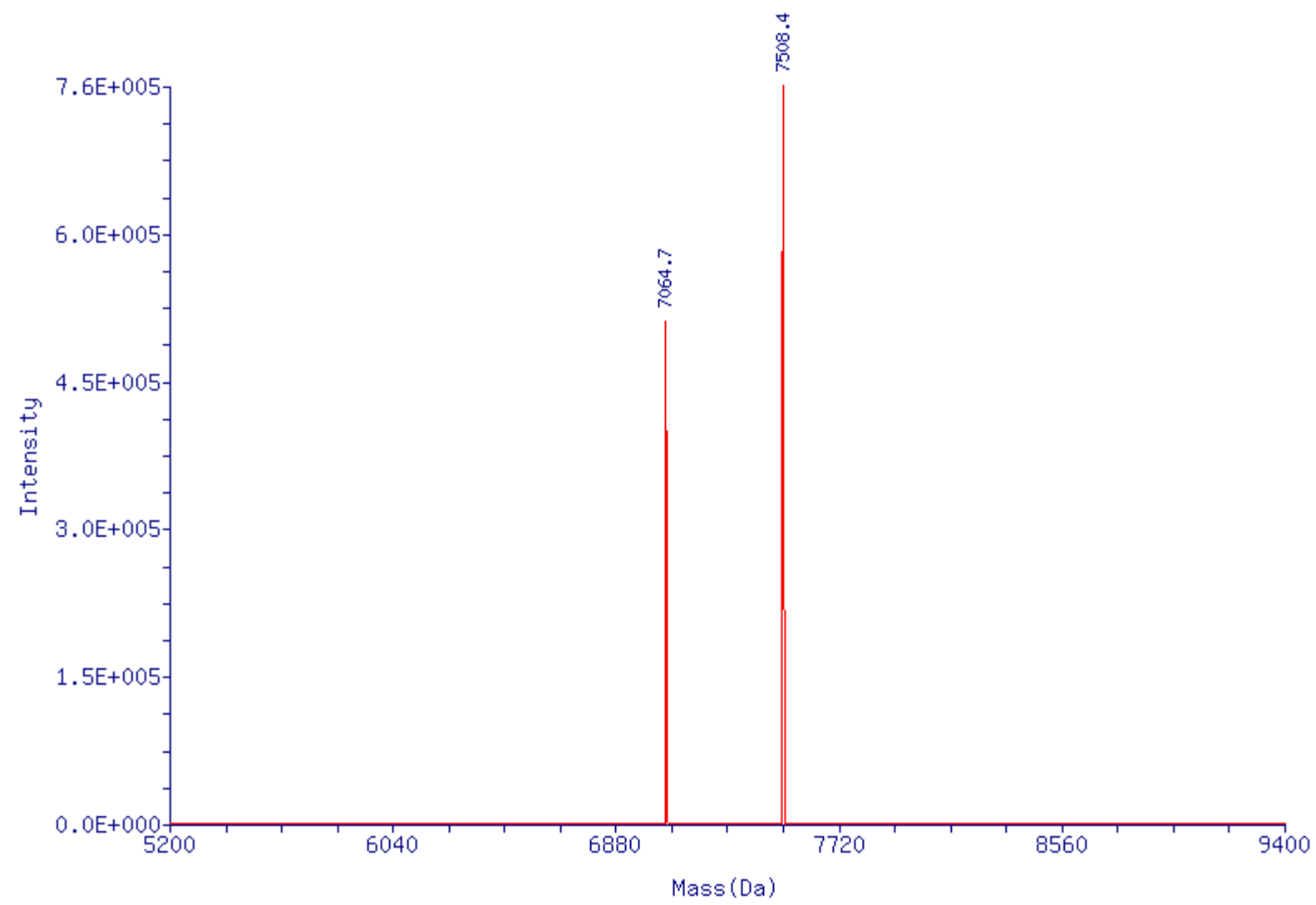

Figure S21. Mass spectrum of CDC20-2.

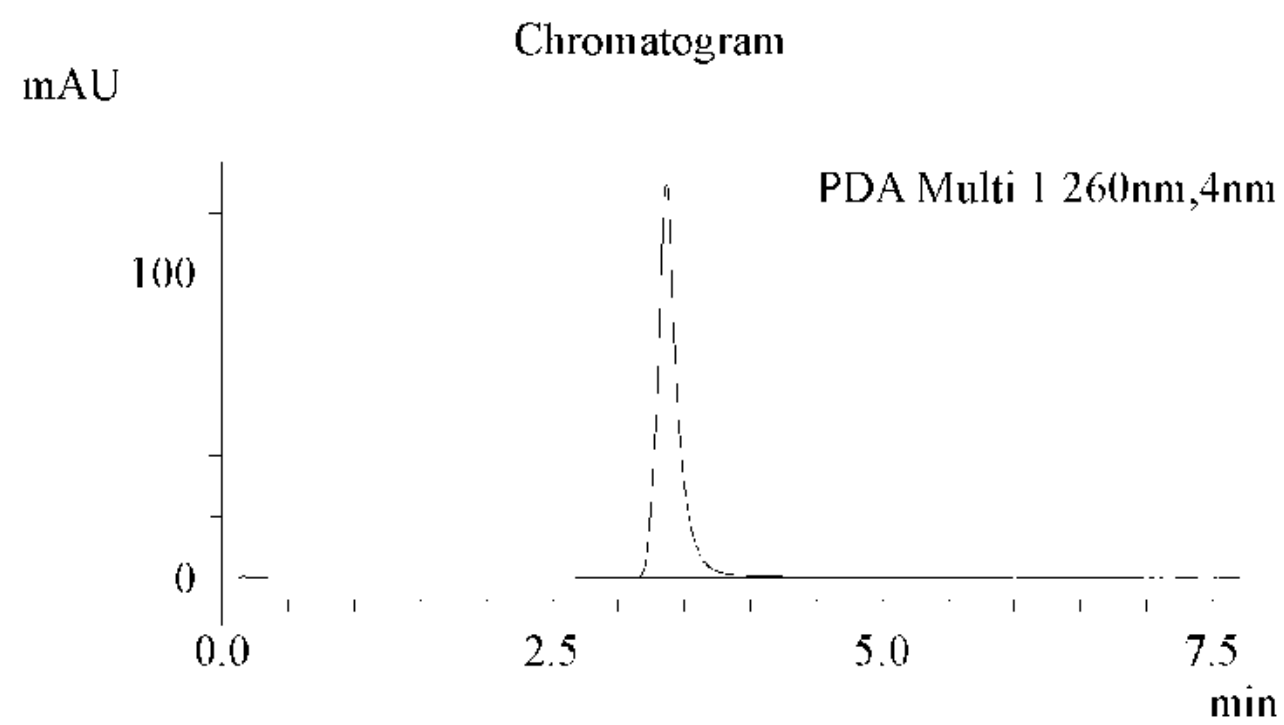

Peak Table

PDA Ch1 260nm

Peak#	Ret. Time	Area	Height	Area%
1	3.205	7189	3430	0.529
2	3.365	1320944	129435	97.116
3	3.696	32033	3338	2.355
Total		1360166	136204	100.000

Figure S22. HPLC chromatogram of CDC20-2.

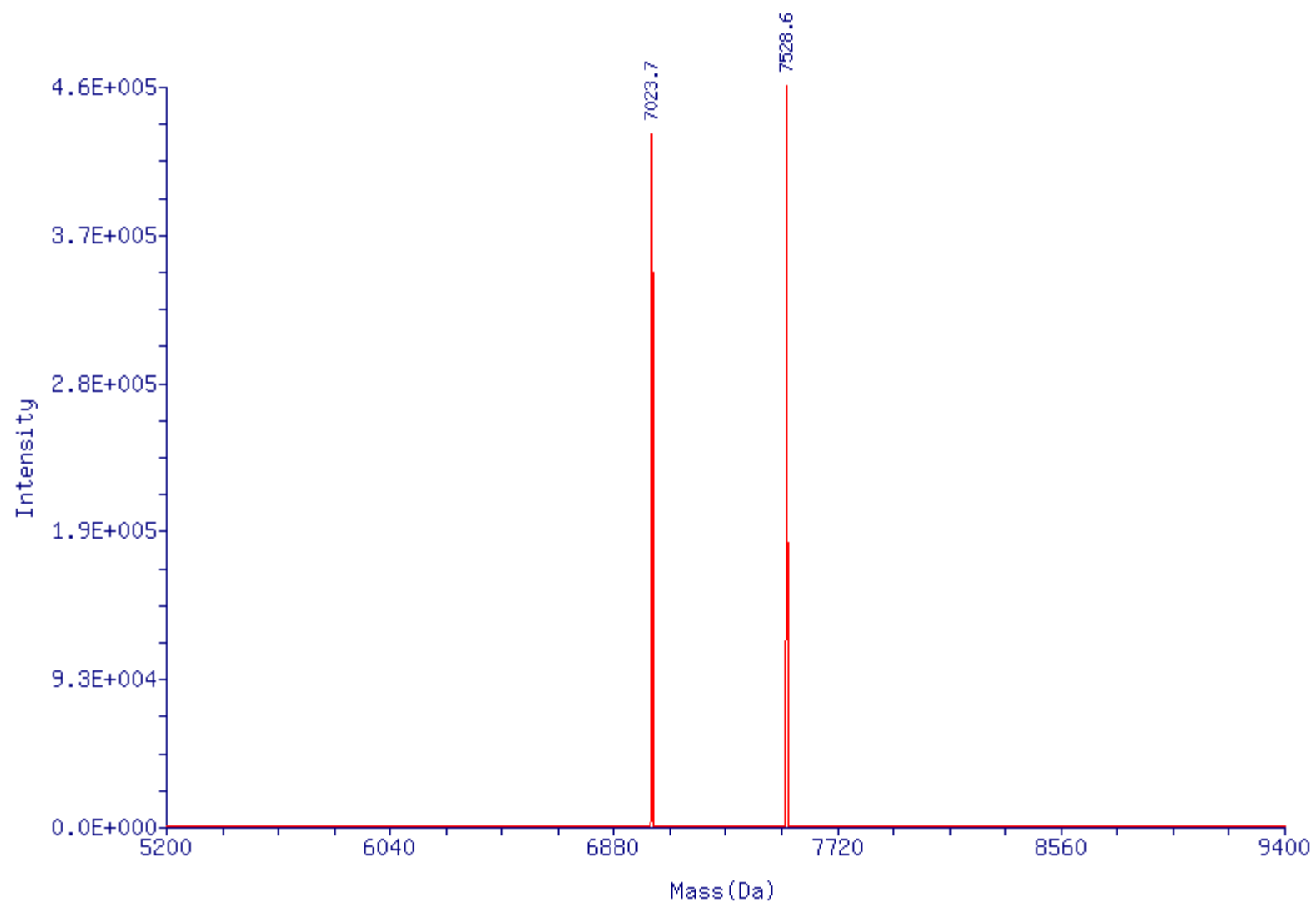

Figure S23. Mass spectrum of TOP2A-1.

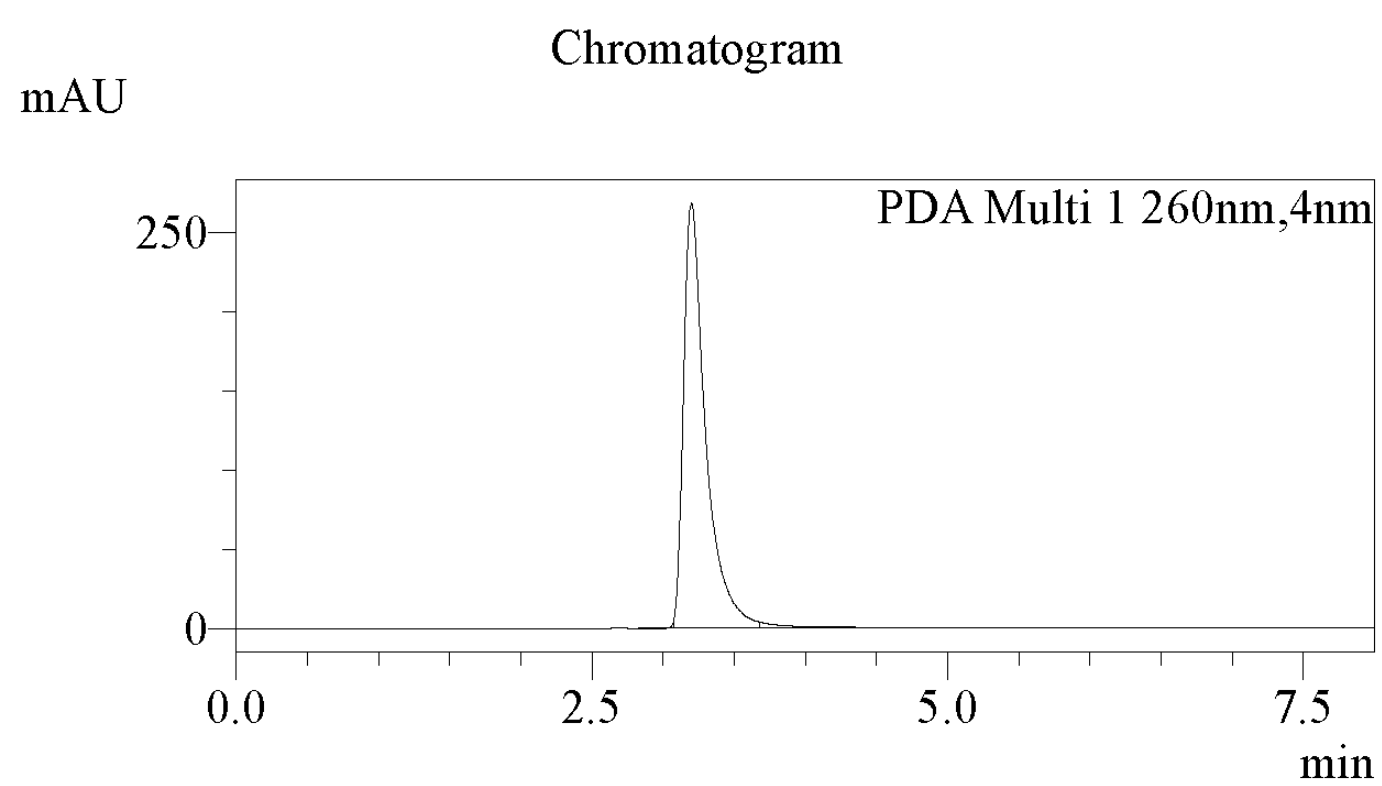

Peak Table

PDA Ch1 260nm

Peak#	Ret. Time	Area	Height	Area%
1	3.067	2118	1245	0.073
2	3.200	2877777	267910	98.781
3	3.696	33381	3142	1.146
Total		2913276	272298	100.000

Figure S24. HPLC chromatogram of TOP2A-1.

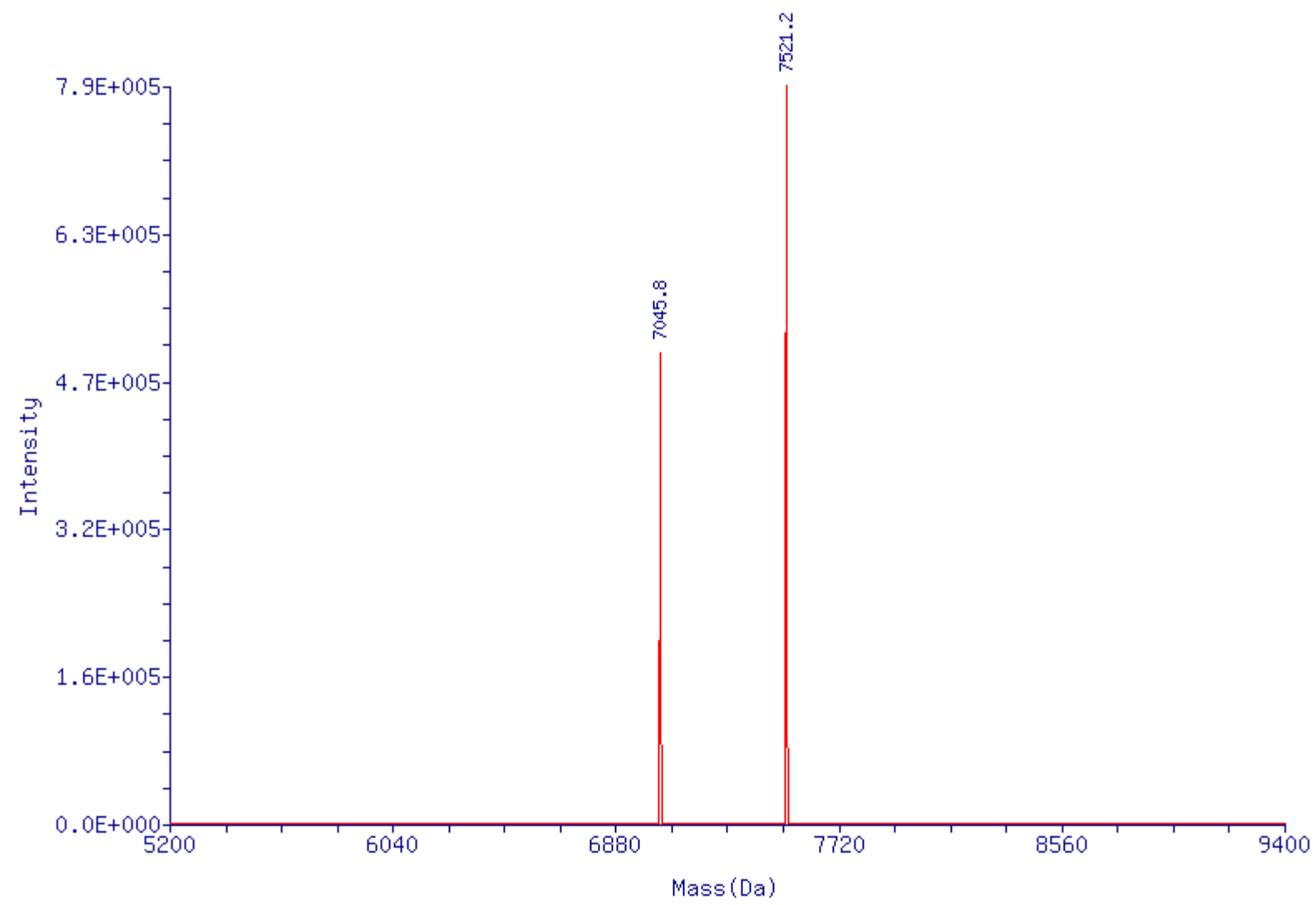

Figure S25. Mass spectrum of TOP2A-2.

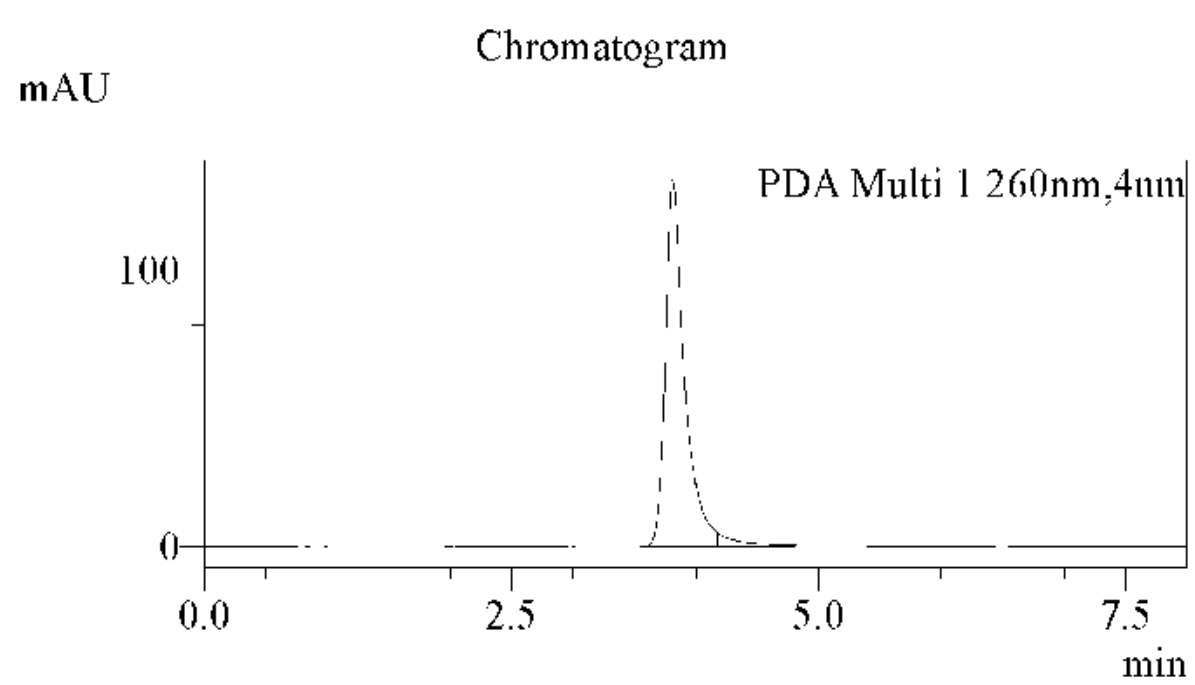

Peak Table

PDA Ch1 260nm

Peak#	Ret. Time	Area	Height	Area%
1	3.664	9331	3611	0.621
2	3.813	1429559	132572	95.196
3	4.187	62809	4643	4.183
Total		1501699	140826	100.000

Figure S26. HPLC chromatogram of TOP2A-2.

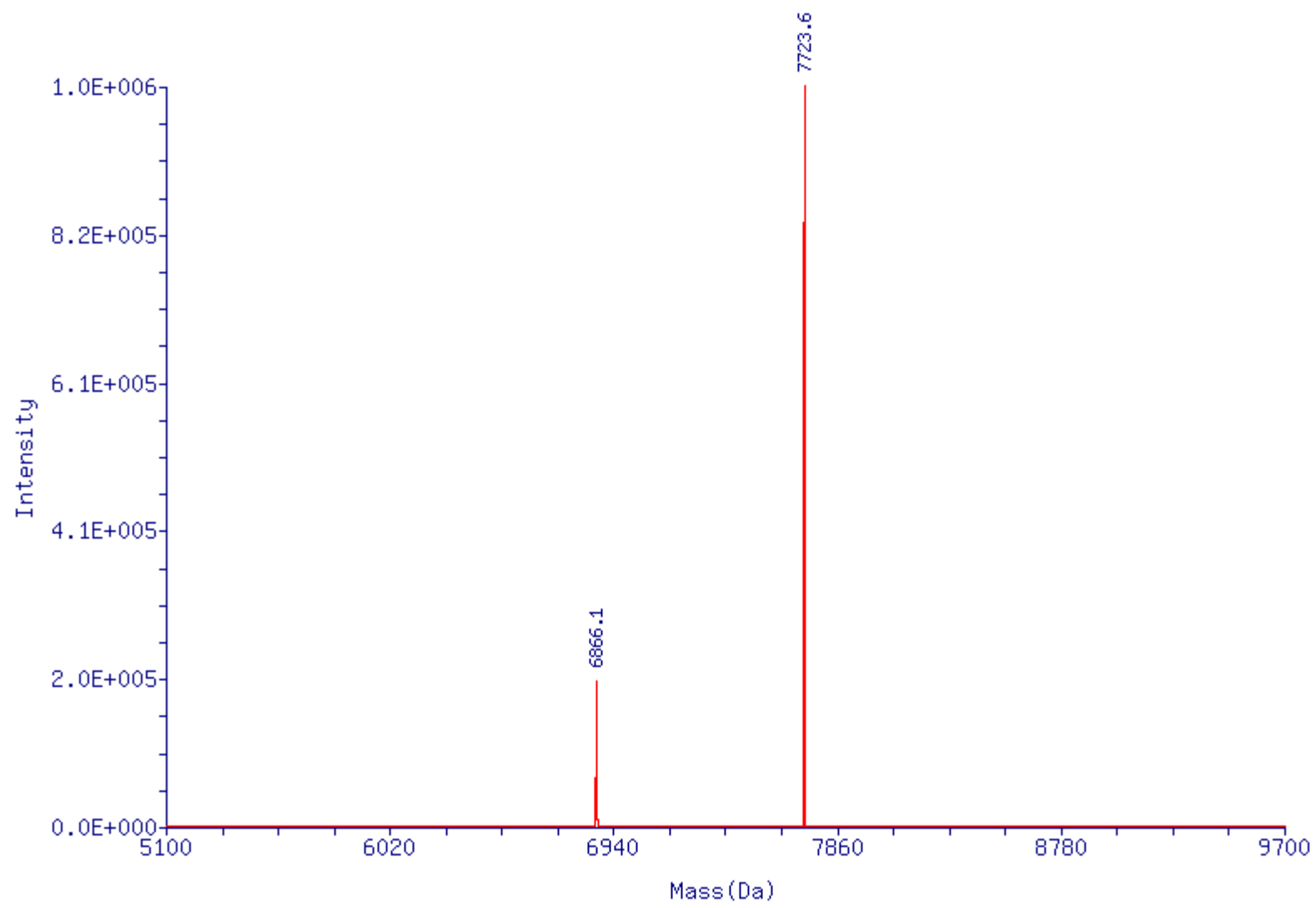

Figure S27. Mass spectrum of TOP2A-3.

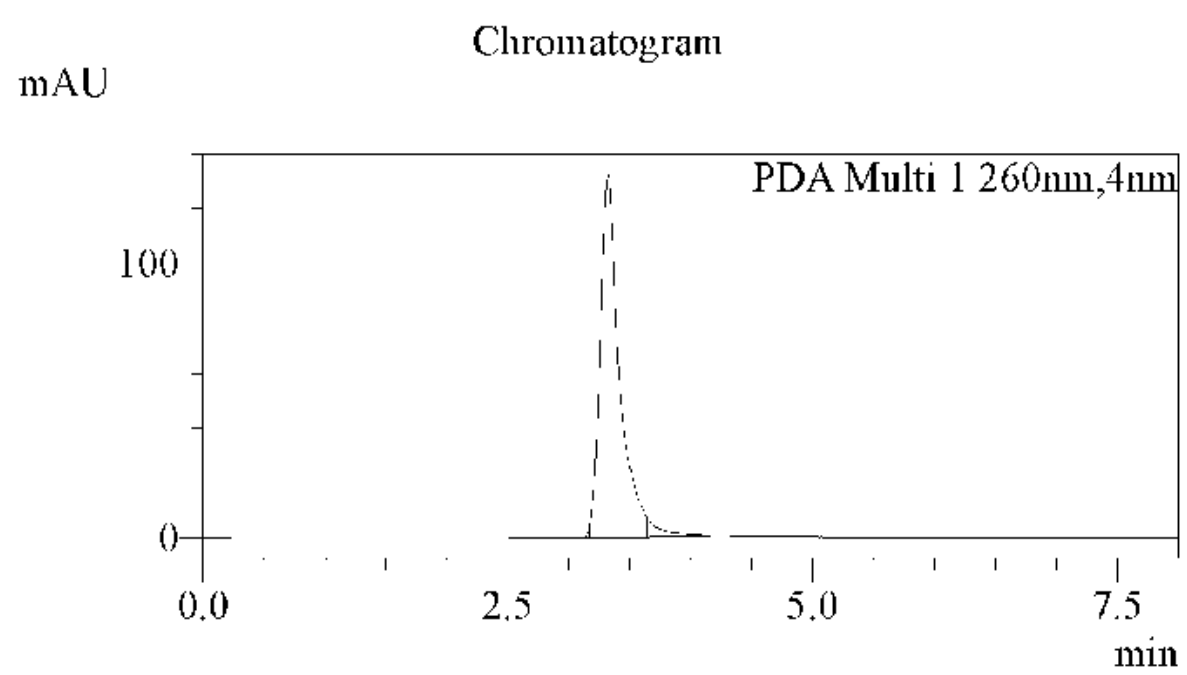

Peak Table

PDA Ch1 260nm

Peak#	Ret. Time	Area	Height	Area%
1	3.163	4615	1922	0.310
2	3.319	1428678	132493	95.929
3	3.653	56021	6439	3.762
Total		1489313	140854	100.000

Figure S28. HPLC chromatogram of TOP2A-3.

Supplemental Tables

Table S1. Leave-one-out cross-validation performance on the single-replicate test set with Inference Augmentation. Reported metrics are MSE, R^2, Pearson r, and Spearman ρ. *"Real" denotes augmentation with genuine Rosetta energy-calculation replicates; "Syn (σ)" denotes synthetic Gaussian-noise augmentation at the indicated noise level σ (as a fraction of the per-feature standard deviation).*

Table S2. Five-fold cross-validation performance on the single-replicate test set with Inference Augmentation. Reported metrics are MSE, R^2, Pearson r, and Spearman ρ. *"Real" denotes augmentation with genuine Rosetta energy-calculation replicates; "Syn (σ)" denotes synthetic Gaussian-noise augmentation at the indicated noise level σ (as a fraction of the per-feature standard deviation).*

Table S3. Hyperparameter search grids used for traditional machine learning models during 5-fold cross-validation*. Optimal hyperparameters were selected via grid search with cross-validated scoring on the training folds.*

Model	Hyperparameter	Search Values
XGBoost	*n_estimators*	50, 100, 200
	max_depth	3, 6, 9
	learning_rate	0.01, 0.1, 0.2
Ridge Regression	*alpha*	0.1, 1.0, 10.0, 100.0
Random Forest	*n_estimators*	50, 100
	max_depth	None, 10, 20
	min_samples_split	2, 5
PLS Regression	*n_components*	2, 3, 4, 5, 6
Lasso Regression	*alpha*	0.01, 0.1, 1.0, 10.0
Elastic Net	*alpha*	0.01, 0.1, 1.0
	l1_ratio	0.2, 0.5, 0.8
Linear SVR	*C*	0.1, 1.0, 10.0, 100.0
	loss	*epsilon_insensitive*, *squared_epsilon_insensitive*

Table S4. Reaction system for removal of genomic DNA (gDNA) from total RNA prior to reverse transcription.

Reagent	Volume
gDNA Clean Reaction Mix	2 μL
Total RNA	1 μg
RNase-free ddH_2O	to 10 μL
Thermal conditions: 42C, 2 min; then 4C, hold	

Table S5. Reverse transcription reaction system used with the Evo M-MLV RT Mix Kit with gDNA Clean for qPCR.

Reagent	Volume
Product from gDNA-removal step (Table S4)	10 μL
5× Evo M-MLV RT Reaction Mix	4 μL
RNase-free ddH_2O	6 μL
Total	20 μL
Thermal conditions: 37C, 15 min; 85C, 5 s; then 4C, hold	

Table S6. RT-qPCR reaction system (20 μL total volume).

Component	Volume	Final Concentration
2× SYBR Green Pro Taq HS Premix	10 μL	1×
Forward primer (10 μM)	0.4 μL	0.2 μM
Reverse primer (10 μM)	0.4 μL	0.2 μM
cDNA template	2 μL	—
RNase-free ddH_2O	to 20 μL	—

Table S7. Primer sequences used for RT-qPCR quantification of CDC20, TOP2A, and the reference gene GAPDH.

Gene	Forward Primer (5'→3')	Reverse Primer (5'→3')
CDC20	CGCTATATCCCCCATCGCAG	GATGTTCCTTCTTGGTGGGC
TOP2A	CAGGGTTCTTGAGCCCCTTC	ATGTAGGTGTCTGGGCGGA
GAPDH	ACGGATTTGGTCGTATTGGG	CGCTCCTGGAAGATGGTGAT

References

1. Murphy, A.H. (1988). Skill scores based on the mean square error and their relationships to the correlation coefficient. Monthly weather review *116*, 2417–2424.

2. Gupta, H.V., Kling, H., Yilmaz, K.K., and Martinez, G.F. (2009). Decomposition of the mean squared error and NSE performance criteria: Implications for improving hydrological modelling. Journal of Hydrology *377*, 80–91. https://doi.org/10.1016/j.jhydrol.2009.08.003.

3. Xu, S., Feng, Q., Qiao, L., Wu, H., Shen, T., Cheng, Y., Zheng, S., and Sun, S. (2025). Benchmarking all-atom biomolecular structure prediction with FoldBench. Nature Communications.

4. Kliuchnikov, E., Maksudov, F., Zuber, J., Hyde, S., Castoreno, A., Waldron, S., Schlegel, M.K., Marx, K.A., Maier, M.A., and Barsegov, V. (2025). Improving the potency prediction for chemically modified siRNAs through insights from molecular modeling of individual sequence positions. Molecular Therapy Nucleic Acids *36*. https://doi.org/10.1016/j.omtn.2024.102415.
