# Supplementary material for: Fast activity prediction of chemically modified siRNAs via structure-based energy calculations and inference-augmented tabular deep learning
Source: Mol Ther Nucleic Acids. 2026 Jul 21;37(3):103029. doi: 10.1016/j.omtn.2026.103029 (PMC13488036; doi:10.1016/j.omtn.2026.103029)
Supplement: Supplementary file 2 [file mmc3.pdf]

Molecular Therapy
Nucleic Acids
Original Article

Fast activity prediction of chemically modified siRNAs via structure-based energy calculations and inference-augmented tabular deep learning

Wenchong Tan,[1] Yiheng Dong,[1] Yuanfang Shi,[1] Nanwen Chen,[1] Shimin Ye,[1] Heng Zhang,[1] Ping Chen,[2] Yinglin Zuo,[2] and Hongli Du[1]

[1]School of Biology and Biological Engineering, South China University of Technology, Guangzhou, Guangdong Province 510000, China; [2]State Key Laboratory of Anti-Infective Drug Discovery and Development, Sunshine Lake Pharma Co., Ltd., Dongguan 523871, P.R. China

Chemical modification is essential for the clinical application of small interfering RNAs (siRNAs), as it improves their stability and specificity. However, predicting the activity of chemically modified siRNAs remains challenging owing to the scarcity of high-quality datasets and the computational expense of molecular dynamics (MD) simulations. In this study, we propose fast and robust activity prediction of chemically modified siRNAs via structure-based energy (FRAMEs), a novel framework that combines rapid structural prediction via deep learning with physics-based energy calculations for feature engineering of siRNA modifications. To address data scarcity, FRAMEs employs inference-augmented tabular deep learning to achieve robust activity prediction. The total energy score correlates strongly with experimental IC50 and melting temperature, achieving performance comparable to MD-based metrics. Under both leave-one-out and stratified 5-fold cross-validation, inference-augmented TabPFN consistently outperformed all classical machine learning baselines, with the two evaluation schemes yielding mutually reinforcing conclusions. Furthermore, by exploiting physically meaningful stochasticity, FRAMEs stabilizes predictions on small datasets and exhibits strong generalization to an independent real-world dataset, outperforming existing methods. Guided by FRAMEs, several fully modified siRNA candidates targeting oncogenes relevant to cancer therapy were designed and experimentally verified. Cell-based gene-silencing assays confirmed their potent knockdown activity, validating the practical utility of FRAMEs for the rational design of therapeutic modified siRNAs.

INTRODUCTION

Small interfering RNAs (siRNAs) hold considerable therapeutic promise for silencing a broad spectrum of disease-causing genes through a conserved post-transcriptional mechanism known as RNA interference (RNAi).[1] In the RNAi pathway, longer double-stranded RNA precursors are processed into short siRNA duplexes by the RNase III-like enzyme Dicer,[2] after which the antisense strand is loaded into the RNA-induced silencing complex (RISC), enabling sequence-specific cleavage of complementary target mRNAs.[3] Despite this potent mechanism, the clinical translation of siRNA therapeutics is hindered by challenges in targeted delivery, sustained *in vivo* efficacy, and safety profiles.[4,5] Numerous nucleic acid modifications have been developed to address these challenges, including phosphonate, ribose, and base modifications.[6] Among these, designs combining 2′-O-methyl (2′-OMe and 2′-F), 2′-fluoro (2′-F), and phosphorothioate (PS) modifications at appropriate positions and ratios have emerged as the predominant strategy in clinical applications.[7,8] Such modification patterns have proven effective in enhancing the stability and specificity of siRNAs while preserving biological safety.[9] However, although these modifications generally improve pharmacokinetic properties, they can also attenuate knockdown efficiency in many instances—indeed, some siRNAs lose their activity upon the application of mature chemical modification patterns, while others do not.[10] Consequently, pharmaceutical developers must still screen numerous sequence variants to identify the optimal design.[11] It is therefore critical to understand the structure-activity relationships of chemically modified siRNAs to guide their rational design.

Many computational methods have been developed to predict siRNA activity, but most are constrained by limited high-quality data, low-resolution modification feature representations, or a lack of interpretability and wet-lab validation. For example, Cm-siRPred employs chemical fingerprint features to predict siRNA activity without capturing the structural impact of modifications.[12] Although our earlier work ENsiRNA laid the groundwork for structure-based activity prediction,[13] it was built on unmodified siRNAs and relied on datasets that do not reflect current clinical modification patterns. More recently, Alnylam Pharmaceuticals published a study probing the structure-activity relationship of modified siRNAs using molecular dynamics (MD). Nevertheless,

Received 28 March 2026; accepted 20 July 2026;
https://doi.org/10.1016/j.omtn.2026.103029.

Correspondence: Hongli Du, School of Biology and Biological Engineering, South China University of Technology, Panyu District, Guangzhou 510006, China.
E-mail: hldu@scut.edu.cn

Published by Elsevier Inc. on behalf of The American Society of Gene and Cell Therapy.
This is an open access article under the CC BY license (http://creativecommons.org/licenses/by/4.0/).

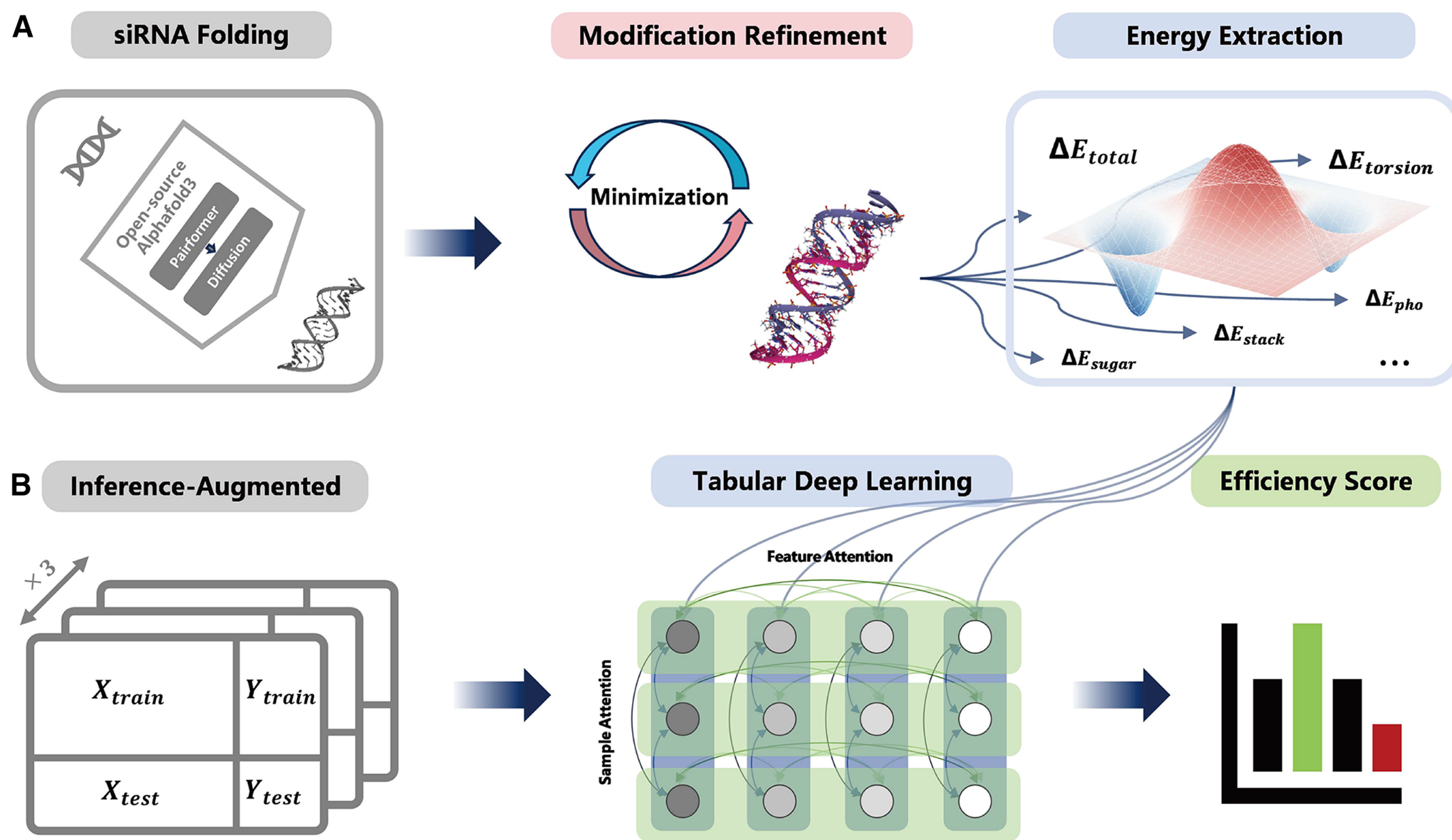

Figure 1. Overview of the workflow

(A) Two-step modified siRNA structure prediction and feature extraction (B) Activity prediction pipeline: energy calculations with inference augmentation.

MD simulations are computationally prohibitive for large-scale screening,[14] limiting their applicability in high-throughput discovery campaigns. Recent advances in deep-learning methods for structure prediction have enabled rapid and highly accurate prediction of RNA structures,[15–17] although current methods still lack the ability to predict the full range of noncanonical modifications required for therapeutics.[18]

To address these limitations, we present a novel computational pipeline (Figure 1) that integrates a deep-learning approach for rapid structure prediction with a modification-refinement protocol based on the Rosetta macromolecular modeling suite,[19] thereby circumventing the prohibitive cost of MD simulations. This pipeline constitutes the first non-MD-based method for predicting modified siRNA structures. Physicochemical energy terms derived from the predicted structures are then used as features for activity prediction via TabPFN,[20] a transformer-based model pre-trained on tabular data, coupled with our proposed inference augmentation strategy. The complete framework—termed FRAMEs (fast and robust activity prediction of chemically modified siRNAs via structure-based energy)—is publicly available at http://www.dulab.com.cn/FRAMEs. To demonstrate real-world utility, we applied FRAMEs to the rational design of therapeutic modified siRNAs targeting key oncogenes in hepatocellular carcinoma (HCC). Cell-based gene-silencing assays confirmed the potency of the selected candidates, validating the reliability of our computational framework. Overall, FRAMEs bridges the gap between rapid structure prediction and activity prediction, offering a practical and experimentally validated tool for therapeutic modified siRNA design. This framework is particularly advantageous for evaluating the non-linear efficacy impact of mature modification patterns on pre-screened candidate sequences.

RESULTS

Validation of modified siRNA structures and energies

One of our principal goals was to develop a fast and accurate method for predicting the structures of siRNA duplexes and their associated energy metrics, which constitutes the foundation for downstream activity prediction. To our knowledge, this is the first non-MD-based method proposed for predicting modified siRNA structures and energy metrics. Based on RNA folding benchmarks and several internal tests, we established our modified siRNA prediction protocol in two steps: an initial unmodified siRNA structure prediction step using a deep-learning-based method, followed by Rosetta-based refinement of the modified siRNA. Protenix, an open-source implementation of AlphaFold 3 that served as the initial structure prediction tool in this study, performed well across several RNA benchmarks.[16,17,21] However, previous benchmarks largely focused on RNA monomers. Since the fully modified siRNA duplex PDB: 2KWG was released in 2010,[22] relying on it as a sole benchmark might unfairly advantage deep learning models (due to potential data leakage) or disadvantage fragment-based methods like FARFAR2. To ensure a fair evaluation, we established a new duplex nucleic acid benchmark using PDB structures released after January 1, 2024. These new structures comprise duplex chains with lengths under 31, similar to siRNAs (Figure S1). Overall, as shown in Figure S2, among open-source methods,[23–25] Protenix exhibited the highest LDDT (Local Distance Difference Test) for small RNA duplex structure prediction. Furthermore, on 2KWG specifically, Protenix outperformed FARFAR2

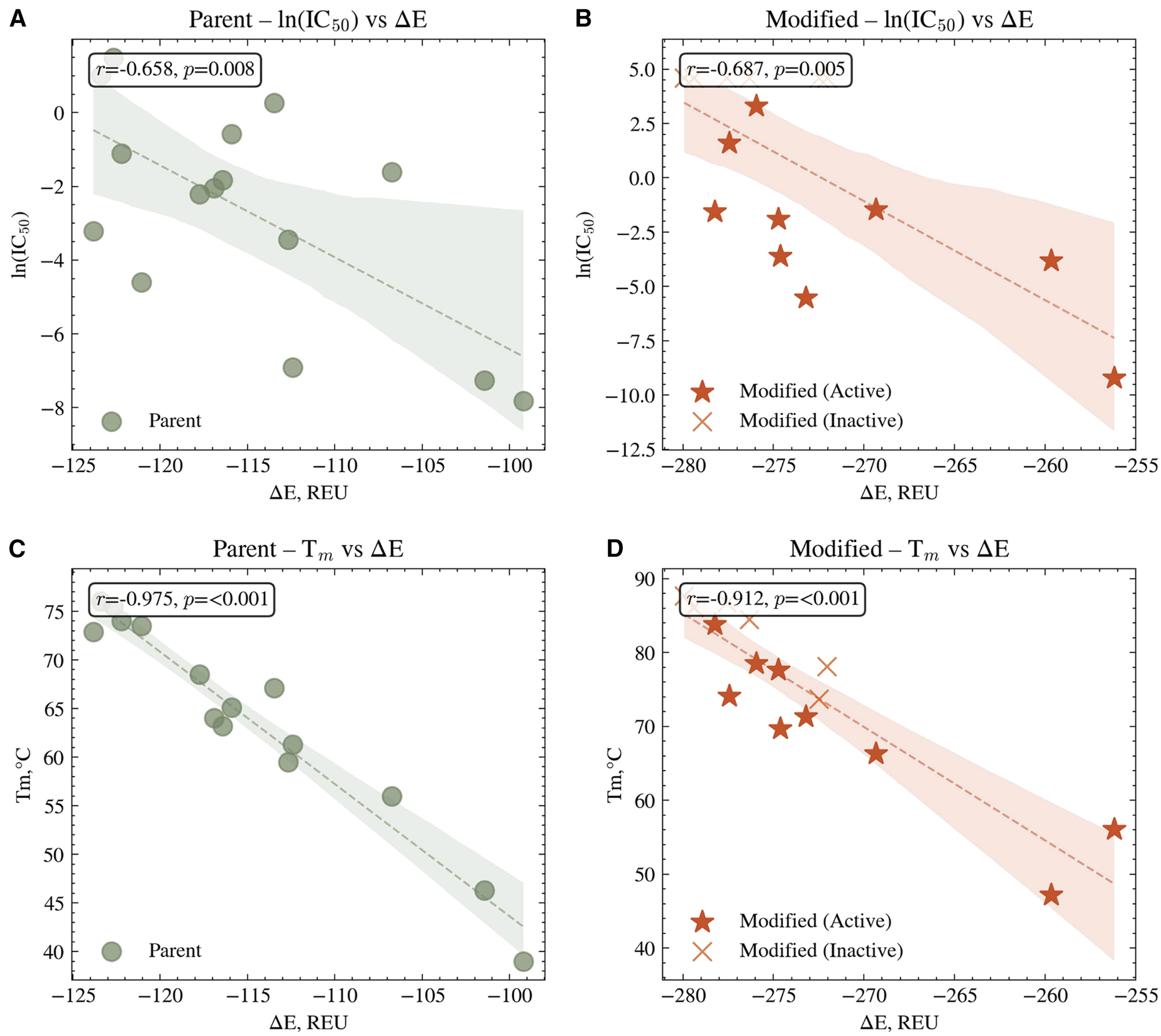

Figure 2. Correlations between Rosetta energy scores and experimental measurements

(A) Scatterplot of ΔE versus $\log_e(IC_{50})$ for parent (unmodified) siRNAs. (B) Scatterplot of ΔE versus $\log_e(IC_{50})$ for chemically modified siRNAs. (C) Scatterplot of ΔE versus T_m for parent siRNAs. (D) Scatterplot of ΔE versus T_m for chemically modified siRNAs. In (B) and (D), asterisks (∗) denote modified siRNAs that retain silencing activity after chemical modification, and crosses (×) denote those that lose silencing activity. Pearson correlation coefficients (r) and corresponding p values are also reported in each panel.

(used in ENsiRNA) as the unmodified siRNA prediction tool: the root-mean-square deviations (RMSDs) of the two-step method using Protenix were 3.101 Å and 3.025 Å, compared with 3.849 Å and 4.088 Å for FARFAR2 (Figures S3A and S3B), which demonstrated that the superior initial structure generated by the deep-learning method makes the most significant contribution.

We further analyzed the correlation between Rosetta-calculated energy terms and experimental properties in the MD dataset by Kliuchnikov et al.[10] As shown in Figures 2A and 2B, the total energy score exhibited a strong negative correlation with $\log_e(IC_{50})$ for both parent ($r = -0.658$, $p = 0.008$) and modified siRNAs ($r = -0.687$, $p = 0.005$), indicating that higher biological activity (lower IC 50) correlates with less negative duplex energy. The full Pearson correlation matrix across all energy terms is shown in Figure S4. This finding recapitulates one of the central conclusions of the MD study, where extensive all-atom MD simulations (approximately 85 μ s of total trajectory time for 15 siRNA pairs) yielded correlations of $r = -0.842$ and $r = -0.844$ for parent and modified siRNAs, respectively. The direction and significance of the correlation are fully consistent: both approaches show that inactive siRNAs cluster at more negative (higher magnitude) interaction energies and higher T_m values, whereas active siRNAs are associated with lower magnitude energies and greater structural flexibility. Notably, this trend—revealed through computationally expensive MD trajectories—is independently reproduced by our pipeline within minutes rather than thousands of CPU-hours, demonstrating that the essential physics captured by long-timescale MD is also accessible through static energy minimization.

As shown in Figures 2C and 2D, the correlation with melting temperature (T_m) was exceptionally strong for parent ($r = -0.975$, $p < 0.001$) and modified ($r = -0.912$, $p < 0.001$) sequences, suggesting that structure-based energy calculations accurately reflect duplex thermodynamic stability. The MD study reported T_m correlations of $r = -0.91$ (parent) and $r = -0.97$ (modified). Our Rosetta-based total energy therefore matches or exceeds the MD-derived energy–T_m correlation, and, crucially, preserves the same qualitative pattern observed in the MD work: chemical modifications generally increase T_m (by an average of approximately 10°C in the experimental data) and increase binding energy, with higher duplex stability tending to be accompanied by greater potency loss for modified siRNAs.[10] Our rapid energy calculations faithfully reproduce this thermodynamic landscape, confirming that the Rosetta energy function serves as a reliable proxy for the free-energy changes that govern siRNA duplex stability.

Furthermore, when benchmarked against the MD study's Partial Least Squares (PLS) model, our structure-based features achieve a higher Pearson correlation ($r = 0.89$, $p = 2.8 \times 10^{-11}$) than the MD-derived model ($r = 0.87$, $p = 5.8 \times 10^{-10}$), even when the latter incorporates experimental T_m values as features; this comparison is detailed in the supplemental materials and Figure S13.

These results are notable given that the traditional MD approach demands meticulous force-field parameterization for 2′-F and 2′-OMe modifications and weeks of wall-clock time on a dedicated compute cluster to accumulate ~1 μs of trajectory per siRNA duplex. Our pipeline reduces this turnaround to mere minutes on a local workstation—a speed-up that not only makes routine screening practical but also enables us to deploy the method as a publicly accessible online service, offering on-demand predictions without requiring users to maintain specialized hardware or software. The concordant—and in some aspects superior—predictive accuracy achieved at this fraction of the computational cost establishes our approach as a practical, cost-effective surrogate for MD in siRNA activity-prediction workflows.

Overall, the strong agreement between our total energy metric and melting temperature supports the physicochemical validity of the modeling approach and provides a solid foundation for downstream activity prediction.

Leave-one-out cross-validation performance comparison

To comprehensively evaluate both classical machine learning and tabular deep-learning approaches, as well as the efficacy of inference augmentation, we designed two complementary sets of experiments. First, we conducted leave-one-out cross-validation (LOOCV), which maximizes the use of the available data and provides an unbiased per-sample performance estimate under the constraint of a limited dataset.

As shown in Figure 3, without inference augmentation, TabPFN offered no advantage over classical machine learning models: PLS achieved the highest R^2 of 0.40 (Pearson $r = 0.64$, Spearman $\rho = 0.60$), and Lasso ranked second with $R^2 = 0.24$ (Pearson $r = 0.50$, Spearman $\rho = 0.40$). The best single TabPFN checkpoint (*real variants*) without inference augmentation attained only $R^2 = 0.13$ (Pearson $r = 0.38$, Spearman $\rho = 0.34$), and all six checkpoints produced comparable results (variation ≤ 0.07). Once inference augmentation was applied, TabPFN performance improved markedly: the *small-samples* checkpoint reached $R^2 = 0.54$ (Pearson $r = 0.74$, Spearman $\rho = 0.67$), the highest among all evaluated models. This checkpoint also showed the largest absolute gain across all checkpoints, rising from $R^2 = 0.09$ to 0.54 (a 45-percentage-point improvement). By comparison, Lasso—the best traditional model with augmentation—reached $R^2 = 0.42$, itself a meaningful improvement from 0.24 without augmentation. Steiger's Z-test confirmed that TabPFN with inference augmentation ($R^2 = 0.54$) significantly outperformed augmented Lasso ($R^2 = 0.42$, $p < 0.05$),[26] and the improvement of TabPFN from no augmentation to inference augmentation was also highly significant ($p < 0.005$). These results indicate that inference augmentation substantially improves model performance, and that TabPFN leverages in-context augmentation data far more effectively than traditional models. A detailed discussion on the origin and magnitude of the performance improvements brought by inference augmentation in TabPFN is provided in the supplemental material and Figures S15–S18.

To probe model robustness under realistic stochastic conditions, we also evaluated all models on a multi-replicate test set, where the three energy-calculation replicates of each held-out siRNA were treated as a single averaged sample. This setting mirrors real-world screening scenarios in which predictions must remain stable across independent energy calculations, placing greater emphasis on generalization ability. As shown in Figure S5, the ranking of models closely paralleled the single-replicate results: TabPFN with inference augmentation again achieved the top R^2 of 0.54, while most models with augmentation showed moderately lower R^2 values relative to Figure 3, with the notable exception of the best TabPFN checkpoint whose R^2 remained stable at 0.54. Specifically, under the multi-replicate test setting, TabPFN with inference augmentation ($R^2 = 0.54$) significantly outperformed augmented Lasso ($R^2 = 0.35$, $p < 0.001$), a larger margin than that observed with single-replicate test data. The augmentation-induced gain for TabPFN was also more pronounced (from $R^2 = 0.15$ to 0.54, $p < 0.001$), indicating that inference augmentation not only improves mean predictive accuracy but also substantially enhances robustness to stochastic variation in energy calculations. Taken together, both single- and multi-replicate LOOCV evaluations consistently demonstrate that inference augmentation enables TabPFN to exploit augmented data with an effectiveness that traditional machine learning cannot match, ultimately yielding the best predictive performance across all conditions.

To further validate the utility of real-world augmentation data, we compared TabPFN with real-world inference augmentation against

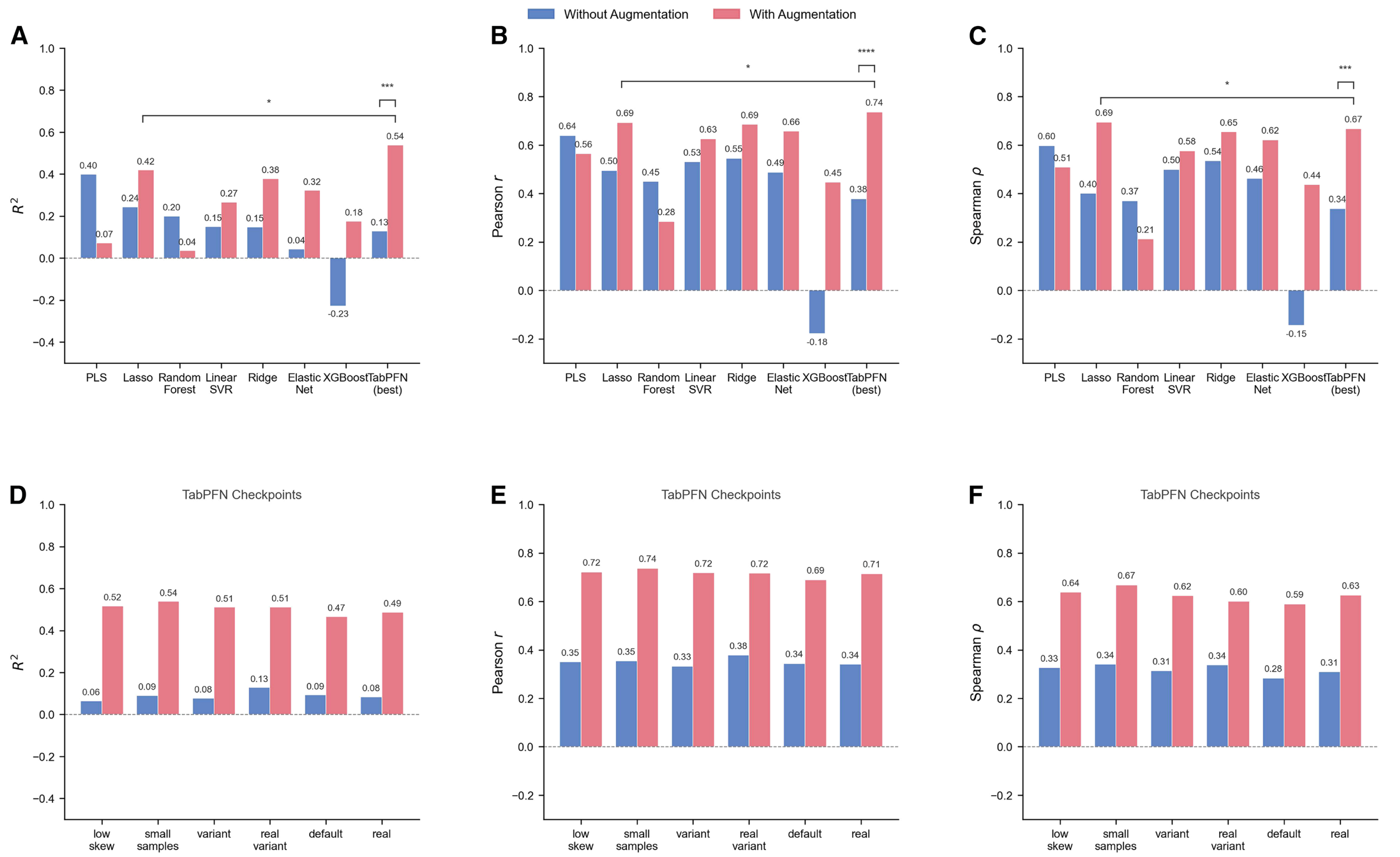

Figure 3. Leave-one-out cross-validation performance on the single-replicate test set

(A–C) Bar charts showing R^2, Pearson r, and Spearman ρ, respectively, for all evaluated models without augmentation (blue) and with augmentation (red). "TabPFN (best)" refers to the highest-performing checkpoint. (D–F) Bar charts showing R^2, Pearson r, and Spearman ρ, respectively, for individual TabPFN checkpoints without augmentation (blue) and with inference augmentation (red). Significance was assessed by Steiger's Z-test: ****$p < 0.001$; ***$p < 0.005$; **$p < 0.01$; *$p < 0.05$.

TabPFN augmented with synthetic Gaussian-noise data (Table S1). Real-world inference augmentation consistently outperformed its synthetic counterparts across all noise levels ($\sigma = 0.01, 0.05, 0.10, 0.50$) and both test-set configurations, demonstrating that genuine physical replicates provide qualitatively superior augmentation signals compared with simulated noise. Notably, this advantage was universal across all TabPFN checkpoints but was not observed for traditional machine learning models, suggesting that TabPFN exploits the physicochemical relationships encoded in replicate energy features in a manner that classical models cannot.

5-fold cross-validation performance comparison

Although LOOCV provides maximum data utilization and unbiased per-sample estimates, it inherently produces highly correlated training sets, which may inflate estimates of model generalization. To assess model stability under a more stringent evaluation scheme, we complemented the LOOCV analysis with stratified 5-fold cross-validation (5-fold CV), which better reflects the variance that would be observed when deploying the framework on genuinely unseen data partitions.

As shown in Figures 4A–4C, without augmentation, the model ranking in 5-fold CV mirrored that of LOOCV: PLS remained the strongest traditional model ($R^2 = 0.28$), while the best TabPFN checkpoint (*low-skew*) achieved only $R^2 = 0.12$. Applying real-replicate inference augmentation again reversed this ordering: the *small-samples* checkpoint rose to $R^2 = 0.54$ (an absolute improvement of 42 percentage points, from 0.12 to 0.54), surpassing all alternative models (Figure 4). Most traditional models showed a decline in R^2 after augmentation was applied, with XGBoost and Linear SVR remaining below zero, indicating that these models cannot benefit from—and may even be harmed by— the additional replicate samples. Because the metric computation differs between LOOCV and 5-fold CV (global pooling versus per-fold averaging), the 5-fold setting is intrinsically harder, and the advantage of TabPFN with inference augmentation over traditional models is consequently more pronounced; the paired t test significance is attenuated by the small number of folds ($n = 5$), but the directional consistency with LOOCV is clear.

Consistent with the LOOCV checkpoint analysis (Figures 3D–3F), Figures 4D–4F shows that the *small-samples* checkpoint paired with inference augmentation ranked highest among all TabPFN checkpoints in 5-fold CV as well, underscoring the importance of checkpoint selection for small-dataset applications.

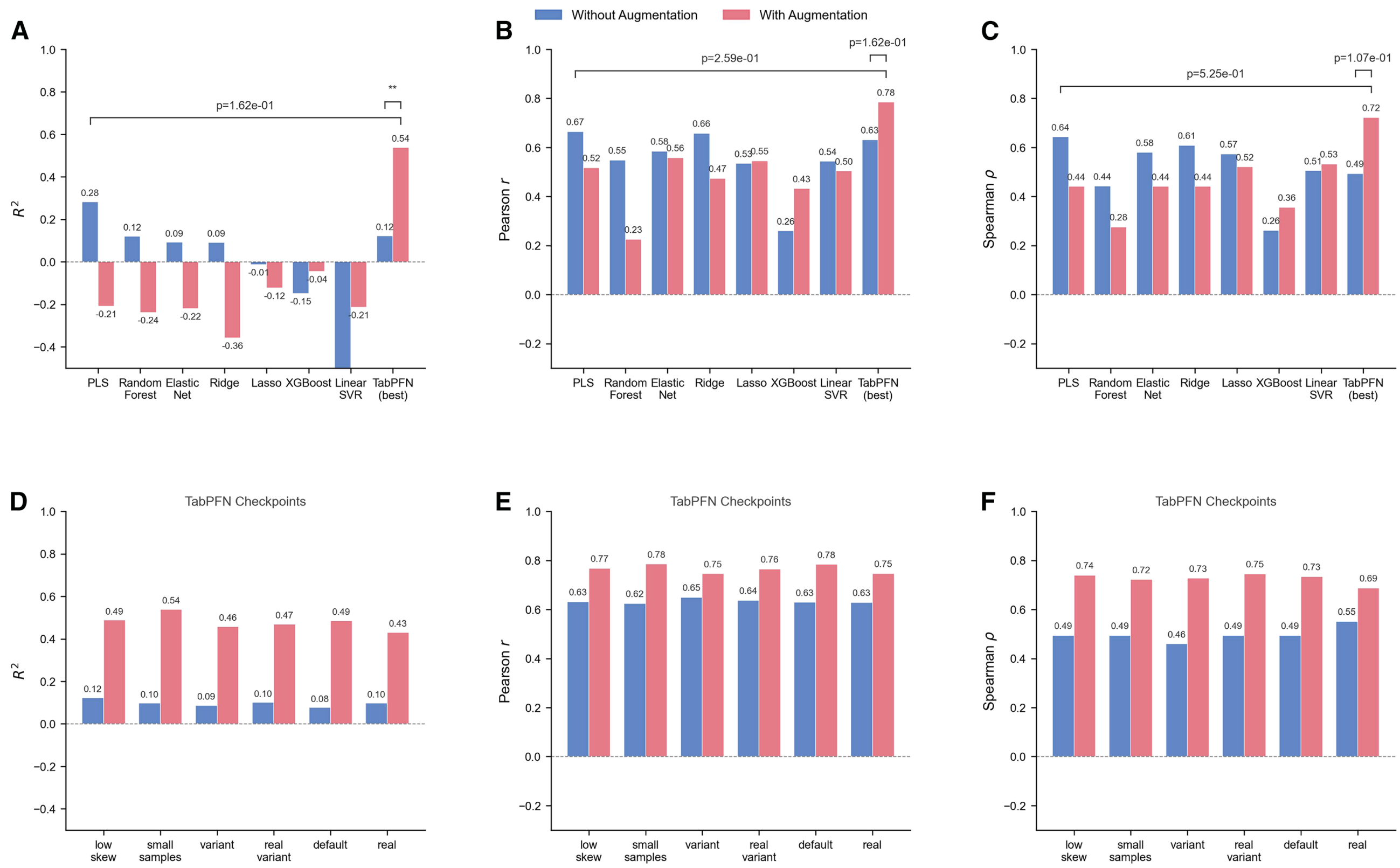

Figure 4. 5-fold cross-validation performance on the single-replicate test set

(A–C) Bar charts showing R^2, Pearson r, and Spearman ρ, respectively, for all evaluated models without augmentation (blue) and with augmentation (red). "TabPFN (best)" refers to the highest-performing checkpoint. (D–F) Bar charts showing R^2, Pearson r, and Spearman ρ, respectively, for individual TabPFN checkpoints without augmentation (blue) and with inference augmentation (red). Significance was assessed by paired t test: ****$p < 0.001$; ***$p < 0.005$; **$p < 0.01$; *$p < 0.05$.

With respect to multi-replicate test performance, Figure S6 confirms that the directional pattern described previously is preserved: TabPFN with inference augmentation continued to outperform all traditional models across the same evaluation conditions, with the best checkpoint achieving $R^2 = 0.54$ under the multi-replicate setting. The stability of the top-ranked checkpoint across both single- and multi-replicate test sets in 5-fold CV reinforces the conclusion drawn from LOOCV: inference augmentation confers robustness to stochastic variability that traditional machine learning models cannot achieve.

In the synthetic augmentation comparison (Table S2), real-world inference augmentation again yielded superior generalization across all TabPFN checkpoints at every noise level, consistent with the LOOCV results. This cross-method agreement provides further confidence that the observed advantage of real-world replicates reflects a genuine property of the augmentation signal rather than an artifact of any specific validation protocol.

Overall, both LOOCV and 5-fold CV consistently demonstrate that inference Aagmentation substantially enhances TabPFN performance on small, noisy biological datasets, with the two validation schemes producing mutually reinforcing evidence for the utility of genuine physical replicates as an augmentation source.

Performance comparison on an external test set

Having demonstrated the accuracy and robustness of our method through structural validation, energy metrics assessment, and cross-validation under diverse conditions, we next evaluated its generalization to out-of-distribution (OOD) data using an independent external test set, which is critical for real-world applicability. We selected the test set reported by ENsiRNA-mod,[13] which originates from the US patent US20230026968A1—a real-world siRNA drug design patent for Givosiran, an FDA-approved RNAi therapeutic. The dataset comprises fully modified siRNAs bearing different positional combinations of 2′-OMe and 2′-F, and PS modifications, all sharing the same Givosiran parent sequence. This external test set differs from the training set in two fundamental respects: first, the sequence identity, sequence lengths, and experimental conditions are distinct; second, and more importantly, the task itself is inverted—whereas the training set evaluates different siRNA sequences bearing the same modification pattern, the external test set evaluates the same sequence with varying modification patterns, representing a genuine OOD challenge. It should be noted that the

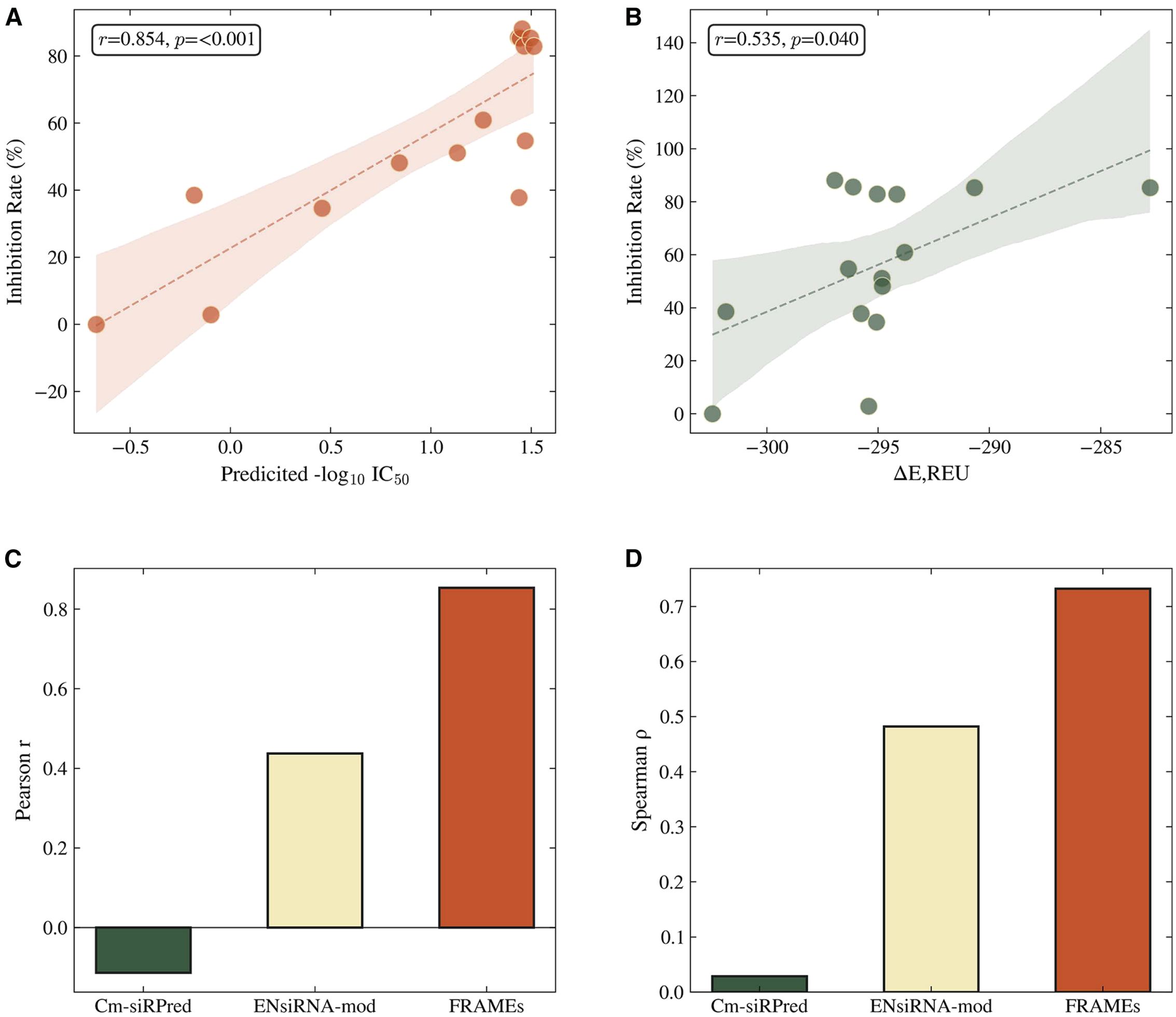

Figure 5. Performance of FRAMEs on the external test set

(A) Scatterplot of FRAMEs-predicted $-\log_{10}(\mathrm{IC}_{50})$ against experimental inhibition rate (%), with a linear regression line; Pearson correlation and *p* value are annotated. (B) Scatterplot of the Rosetta energy units (ΔE, REU) against experimental inhibition rate (%), with a linear regression line; Pearson correlation and *p* value are annotated. (C) Bar chart comparing the Pearson correlation coefficient (*r*) of Cm-siRPred, ENsiRNA-mod, and FRAMEs with experimental inhibition rate. (D) Bar chart comparing the Spearman rank correlation coefficient (ρ) of Cm-siRPred, ENsiRNA-mod, and FRAMEs with experimental inhibition rate. In (C) and (D), bars are colored in red, yellow, and green for Cm-siRPred, ENsiRNA-mod, and FRAMEs, respectively.

external test set reports experimental inhibition rates (%) at a fixed siRNA concentration rather than IC50 values. Under a fixed-dose, single-concentration assay and a sigmoidal dose-response model, the rank order of potency is preserved when IC50 values span a sufficiently wide range relative to the assay concentration. Accordingly, rank-based metrics (Spearman ρ) between predicted $-\log_{10}(\mathrm{IC}_{50})$ and observed inhibition rate are strictly valid; Pearson r is reported alongside them for completeness, with the caveat that the two quantities are not numerically interchangeable.

Here, we evaluated our framework—using the best inference-augmented tabular deep-learning checkpoint fitted on the full training dataset, referred to as FRAMEs—against ENsiRNA-mod and Cm-siRPred using the protocol described in the preceding sections. As shown in Figure 5A, the predicted $-\log_{10}(\mathrm{IC}_{50})$ values achieved Pearson $r = 0.854$ and Spearman $\rho = 0.732$ against experimental inhibition rates, representing improvements of 94.98% and 51.87% over ENsiRNA-mod (Pearson $r = 0.438$, Spearman $\rho = 0.482$), respectively. For comparison, the non-inference-augmented model achieved a Pearson r of 0.726 and a Spearman ρ of 0.579 on this external test set. A direct comparison of Pearson and Spearman correlations across FRAMEs, ENsiRNA-mod, and Cm-siRPred is presented in Figures 5C and 5D. Although ENsiRNA-mod improves upon Cm-siRPred by incorporating parent structure information, its accuracy is ultimately limited by the lack of a high-quality training dataset and, most importantly, by the absence of physics-based modified siRNA structural features. In contrast, despite the small size of its training set, FRAMEs leverages computed physical energy

Table 1. Experimental validation of computationally predicted chemically modified siRNA candidates targeting *CDC20* and *TOP2A*

siRNA ID	Target	Passenger sequence (5′–3′)	Guide sequence (5′–3′)	Predicted pIC 50	Predicted IC 50 (μ M)	Exp. inhibition rate
CDC20-1	*CDC20*	c*a*agaaGfGfAfacaucagaaa	u*Uf*uCfuGfaUfgUfuCfcUfuCfuUfg*g*u	1.023	0.0948	0.850 ± 0.0146
CDC20-2	*CDC20*	c*a*aggagaAfcCfAfGfccugaaaa	u*Uf*UfucaGfgcUfgguuCfuCfcuug*c*u	2.032	0.0093	0.940 ± 0.0068
TOP2A-1	*TOP2A*	g*g*agaagaUfuAfUfAfcauguauc	g*Af*UfacAfugUfauaaUfcUfucucc*a*u	1.098	0.0798	0.898 ± 0.0403
TOP2A-2	*TOP2A*	a*a*gaagugUfuCfAfGfcuguaaaa	u*Uf*UfacAfgcGfaacaCfuCfuucuu*a*g	1.075	0.0841	0.931 ± 0.0098
TOP2A-3	*TOP2A*	c*g*auuguuAfuUfUfCfCfaccaaaa	u*Uf*uugGfugGfaaauAfaCfaaucg*g*u	2.934	0.0012	0.948 ± 0.0105

Candidates were selected based on low predicted IC50 values from the inference-augmented TabPFN model and subsequently assessed by cell-based gene-silencing assays. Passenger and guide sequences are listed in 5′–3′ orientation. Predicted pIC 50 denotes $-\log_{10}(IC_{50}/M)$; predicted IC 50 is reported in μ M. Experimental inhibition rate is expressed as a fraction of the negative-control condition. 2′-OMe: lower case; and 2′-F: Nf ("N" being either A, C, G, or U); Phosphonate: *.

features through inference-augmented tabular deep learning, endowing the model with strong OOD generalization potential. Furthermore, the physics-based nature of the FRAMEs feature set inherently provides greater interpretability than purely data-driven deep-learning approaches such as ENsiRNA-mod and Cm-siRPred. As shown in Figure 5B, the total energy ΔE also exhibits a statistically significant correlation with inhibition rate (Pearson $r = 0.535$, $p = 0.040$), consistent with the trend observed in the training set and further confirming that less negative duplex energy is associated with greater siRNA potency.

Overall, these results demonstrate that FRAMEs generalizes effectively to OOD data, substantially outperforming existing methods on an independent real-world test set derived from an FDA-approved drug candidate, highlighting the advantage of integrating physics-based structural features with inference-augmented tabular deep learning for chemically modified siRNA activity prediction.

Experimental validation of predicted candidates

To assess the translational potential of our framework, we applied FRAMEs to guide the design of fully modified siRNA candidates targeting two oncogenes implicated in HCC.

1. Target: *CDC20*

Cell division cycle 20 (*CDC20*) is a critical regulator of the cell cycle and is frequently overexpressed in HCC,[27] where its elevated expression correlates with enhanced tumor cell proliferation, resistance to apoptosis, and poor clinical prognosis.[28] RNAi-mediated silencing of *CDC20* induces G2/M phase cell-cycle arrest and promotes apoptosis in HCC cells, establishing it as a compelling therapeutic target for liver cancer.[29]

2. Target: *TOP2A*

DNA topoisomerase II alpha (*TOP2A*) is an essential enzyme involved in DNA replication and chromosome segregation.[30] It is markedly upregulated in HCC,[31] where its overexpression drives tumor progression and metastatic dissemination.[32] Pharmacological or molecular inhibition of *TOP2A* has been proposed as a viable therapeutic strategy in this malignancy.[33]

Notably, the siRNAs employed in prior studies targeting these genes are unmodified, which substantially limits their *in vivo* stability and therapeutic applicability.[29,33] To address this gap, we applied FRAMEs to design chemically modified siRNA candidates against both targets. Briefly, candidate sequences were first generated through conventional unmodified siRNA design pipelines incorporating efficiency prediction, off-target assessment, and thermodynamic screening. These candidates, bearing a clinically representative modification pattern, were subsequently scored by FRAMEs; sequences with a predicted pIC 50 greater than 1 (corresponding to IC 50 < 0.1 μ M) were retained as high-confidence candidates. A representative subset was then selected for experimental validation, balancing screening throughput with predictive confidence. Gene-silencing activity was assessed in cell-based assays by quantifying mRNA inhibition rates at a siRNA concentration of 10 nM, a concentration widely adopted in *in vitro* siRNA efficacy screening.

As shown in Table 1, all selected candidates achieved mRNA inhibition rates exceeding 85%. This level of activity is consistent with the performance observed on the independent external test set, indicating that FRAMEs generalizes across distinct gene targets, sequence lengths, modification patterns, and cell lines. To our knowledge, this represents the first experimentally validated application of a deep-learning-based framework to the rational design of chemically modified siRNA candidates.

Rosetta-based energy analysis and feature interpretability

To further elucidate the relationship between siRNA activity and positional energy, we performed a positional energy analysis, followed by an ablation study and SHAP (SHapley Additive exPlanations) analysis to characterize feature importance and improve the interpretability of our model.[34]

As shown in Figure S7 in the supplemental materials, we first identified guide-strand positions whose per-residue energy values show a statistically significant correlation with $\log_{10}(IC_{50})$ in parent siRNAs: g18 ($r = -0.725$), g3 ($r = -0.718$), g2 ($r = -0.588$), and g14 ($r = -0.443$). Although no position reaches statistical significance for modified siRNAs, the correlation pattern mirrors that of parent siRNAs, with g2 ($r = -0.481$), g18 ($r = -0.467$),

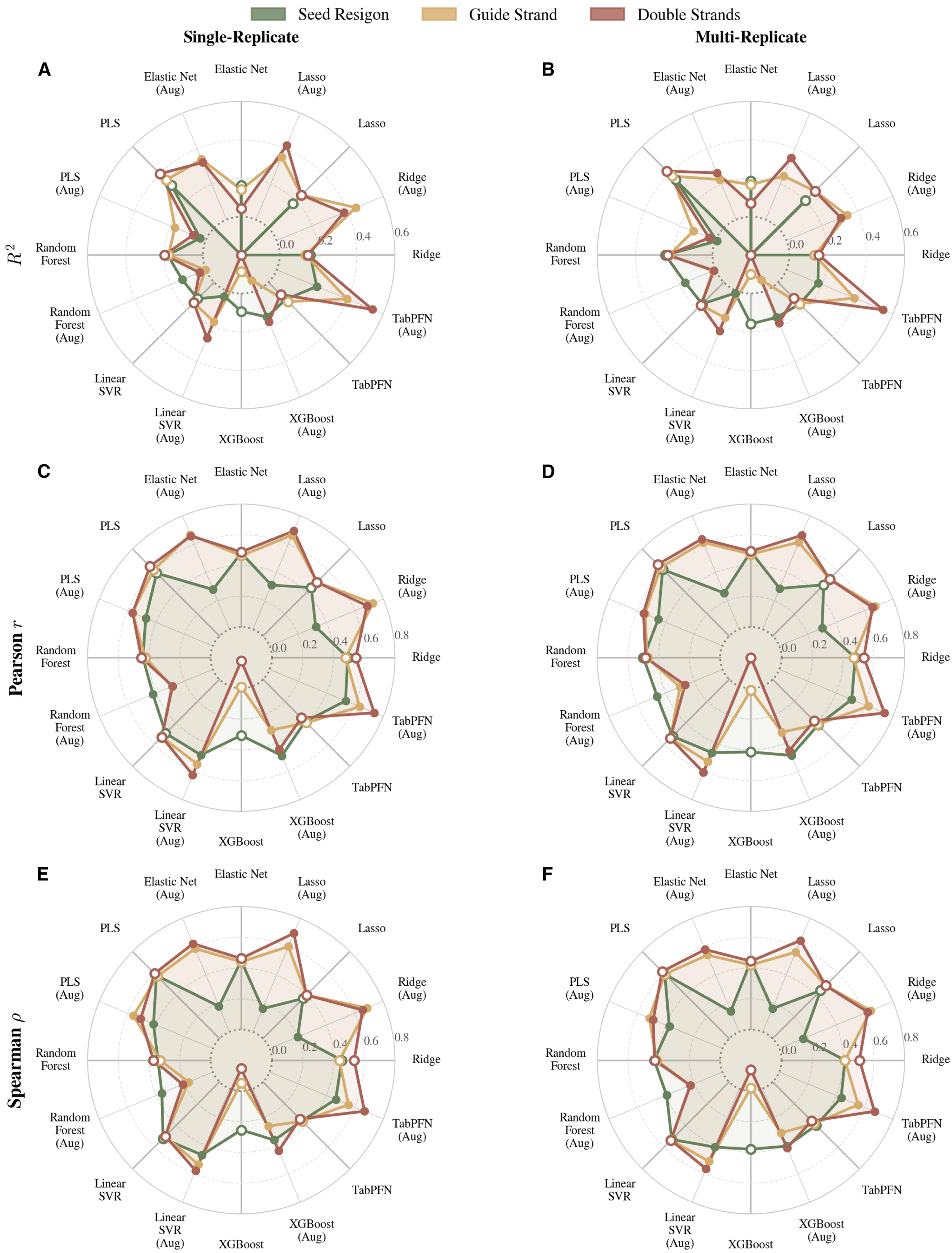

(legend on next page)

and g3 ($r = -0.413$) retaining the highest correlations. Consistent with the MD study, position g2 exhibits one of the strongest correlations with $\log_{10}(IC_{50})$ among parent siRNAs ($r = -0.481$, $p = 0.069$), independently corroborating the reliability of our method. Beyond the seed region emphasized in the MD study, position g18 at the 3′ end of the guide strand also shows a high correlation with $\log_{10}(IC_{50})$ in both parent and modified siRNAs, indicating that the 3′ terminus plays an important role in activity. Moreover, several passenger-strand positions exhibit notable correlations with $\log_{10}(IC_{50})$ in both parent and modified siRNAs: p15 ($r = -0.658$, $p = 0.008$ in parent; $r = -0.623$, $p = 0.013$ in modified), p14 ($r = -0.467$, $p = 0.079$ in parent; $r = -0.623$, $p = 0.013$ in modified), and p5 ($r = -0.561$, $p = 0.030$ in parent; $r = -0.500$, $p = 0.058$ in modified). Notably, p5 carries a 2′-F modification on the passenger strand, and p14 base-pairs with g6 on the guide strand, which is also 2′-F modified. This suggests that residues bearing 2′-F modifications tend to adopt higher-energy and more structurally flexible conformations.

Second, to evaluate the contribution of positional features beyond the seed region, we compared the global feature set against feature subsets restricted to the seed region or the guide strand alone. Under cross-validation, results from both LOOCV and 5-fold CV are presented in Figure 6 and Figure S8, respectively. For TabPFN with inference augmentation, the best-performing model in this study, incorporating the global feature set consistently yielded higher predictive accuracy than using guide-strand-only or seed-region-only features under both validation schemes. In the single-replicate setting, R^2 increased monotonically with the breadth of the feature set—from seed-region-only to guide-strand-only to global—reaching 0.18, 0.38, and 0.54 in LOOCV, and 0.05, 0.23, and 0.54 in 5-fold CV, a trend that held in the duplicate setting as well. Across all three metrics (R^2, Pearson r, and Spearman ρ), the global feature set outperformed the restricted alternatives in the majority of cases, particularly for models with stronger overall predictive capacity. This pattern suggests that models capable of capturing complex feature-target relationships benefit substantially from the broader positional context, providing additional evidence for the functional relevance of regions outside the seed.

On the other hand, as shown in Figure S9 in the supplemental materials, under full-dataset fitting, positional features from both strands consistently outperformed guide-strand-only features, which in turn outperformed seed-region-only features across all evaluated models. Although no substantial performance differences were observed among different TabPFN checkpoints with inference augmentation under full-dataset fitting, the full dual-strand feature set still achieved the highest overall predictive accuracy across all evaluated models. Taken together, both cross-validation and full-dataset fitting results consistently support the global feature set as the preferred choice for prediction and downstream analysis.

Finally, we conducted a SHAP analysis to investigate the contribution of each feature to model predictions (Figure S10). Positions p18, p15, g18, and p14 exhibit the four largest mean absolute SHAP values. Notably, p15, g18, and p14 also show significant correlations in the position-specific energy analysis, demonstrating strong consistency between the two independent analyses and further supporting the validity of our feature engineering strategy.

Overall, these results indicate that the seed region is strongly correlated with siRNA activity but is not the sole determinant. The 3′ end of the guide strand also contributes substantially, and the passenger-strand findings highlight the functional importance of 2′-F-modified positions. Furthermore, the ablation study not only confirms the value of full dual-strand positional features but also emphasizes that only a well-designed approach such as inference augmentation can fully exploit the information contained in complex feature sets within the tabular deep-learning framework.

DISCUSSION

In this study, we developed a structure-based computational framework for the rational design of chemically modified siRNAs. Our findings confirm that computational energy scores derived from static structural models effectively serve as proxies for thermodynamic stability and biological activity, providing a faster and more scalable alternative to MD simulations. The strong correlation between the total energy score and melting temperature validates the physical realism of our modeled structures, while the correlation with IC50 demonstrates the downstream utility of these features for activity prediction.

A further key contribution of this work is the proposed inference augmentation strategy, which substantially improves the performance of tabular deep learning models on biological data. Biological data frequently suffer from elevated noise-to-signal ratios and limited sample sizes.[35] Without augmentation, TabPFN offered no advantage over conventional models; however, once real replicate calculations were incorporated as augmentation data, its predictive accuracy improved markedly under both LOOCV and 5-fold CV. Crucially, the directional conclusions from both evaluation schemes are in strong agreement: the small-samples checkpoint consistently ranked first, and real-world augmentation outperformed synthetic Gaussian noise across all models and test-set configurations. This

Figure 6. Radar chart comparison of model performance across feature subsets

Evaluation of different positional energy feature subsets using replicate-aware leave-one-out cross-validation (all replicates of each siRNA held out together) The evaluation metrics are R^2 (A and B), Pearson r (C and D), and Spearman ρ (E and F). (A), (C), and (E) show performance on the single-replicate test set, while (B), (D), and (F) display performance on the multi-replicate test set. Three feature configurations are compared: full dual-strand features (guide- and passenger-strand features; red), guide-strand-only features (yellow), and seed-region-only features (green). Each axis corresponds to an evaluated machine learning or tabular deep learning model; a larger enclosed area indicates superior generalization across models. Solid markers represent models with inference augmentation; hollow markers represent models without augmentation.

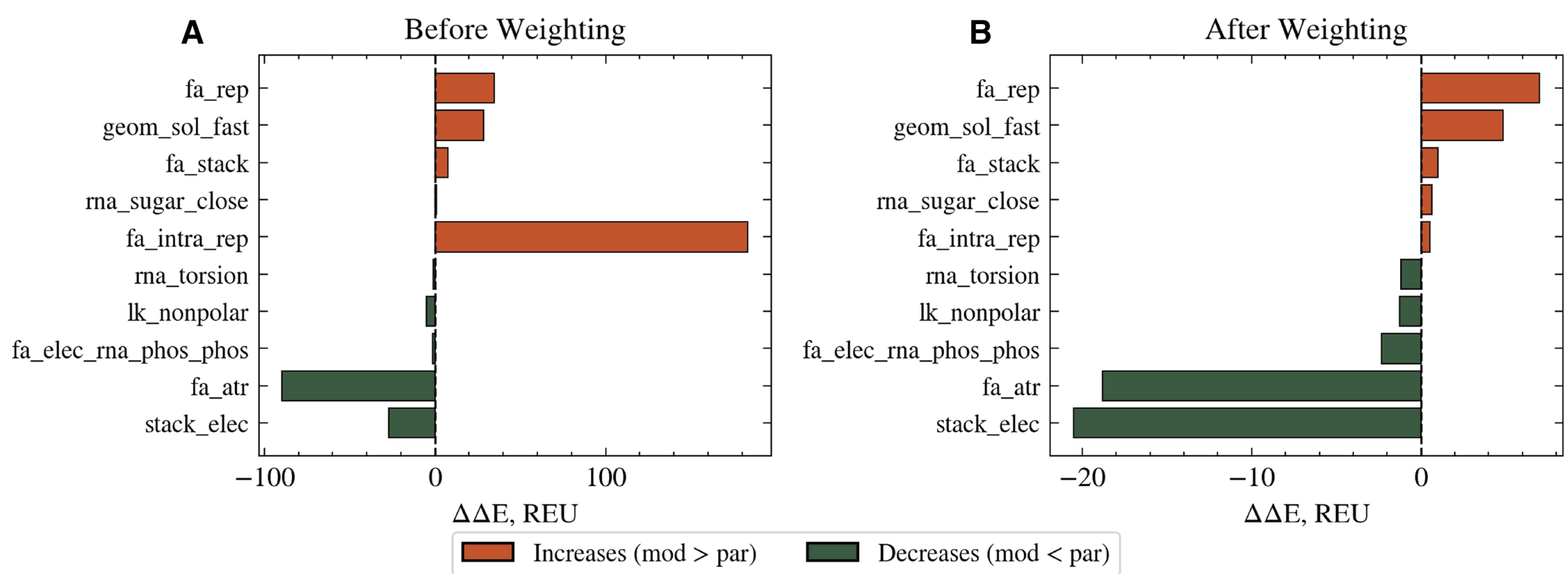

Figure 7. Breakdown of energy changes ($\Delta\Delta E = \Delta E_{mod} - \Delta E_{unmod}$) by score function terms
(A) and (B) show the contributions of individual energy terms before and after score function weighting, respectively. Red bars represent components where the modification increases the energy, while green bars represent components where the modification decreases the energy.

cross-method consistency provides robust evidence that the observed performance gains reflect a genuine property of the augmentation strategy rather than an artifact of the chosen validation protocol. The superior performance of real-world augmentation further supports this view: because TabPFN's in-context learning treats the entire augmented training context as a prior for Bayesian-style inference, it naturally models the output uncertainty arising from replicate variation in energy calculations. Classical models, by contrast, treat replicate samples as independent, identically distributed observations and therefore cannot exploit the structured stochasticity encoded in genuine physical replicates in the same way. Tabular deep learning is well suited to the data scales typical of biomedical research,[36–39] where small sample sizes and elevated noise-to-signal ratios are the norm; the inference augmentation strategy proposed here may therefore prove useful across a range of biomedical domains.

At the same time, although the Rosetta energy scoring function performs well in differentiating parent and modified siRNAs and reflects the relative energy changes upon modification, the computed energy gap between parent and modified siRNAs may deviate from the absolute experimental differences. This leads to a reduced cross-group correlation between Rosetta energy units (REU) and T_m (Pearson $r = -0.5159$; Spearman $\rho = -0.8167$). To further understand the contribution of individual energy terms to the modification-induced changes in Rosetta, we evaluated the specific energy components most affected by chemical modifications. By decomposing the detailed energy changes ($\Delta\Delta E = \Delta E_{mod} - \Delta E_{unmod}$), we can better understand the driving forces behind modification-induced energy changes. As shown in Figure 7, the electrostatic contribution to base stacking (stack_elec) and the long-range Lennard-Jones attractive forces (fa_atr) make the largest stabilizing contributions (indicated by negative ΔΔE values). Since fa_atr reflects van der Waals (vdW) interactions—the dominant stabilizing forces for modified siRNAs in MD studies—these findings are consistent with previously reported results. Specifically, while fa_stack captures base-stacking geometry, the electrostatic stacking term (stack_elec) better reflects the altered physicochemical landscape following extensive 2′ modifications. However, despite the score function weighting balancing the relative contributions of these terms, the absolute energy gap between parent and modified siRNAs remains unresolved and warrants further experimental validation and refinement of the scoring function. These observations not only shed light on the mechanism by which chemical modifications affect siRNA duplex stability but also provide guidance for the future development of an accurate scoring function for modified siRNAs.[40]

We also examined the relationship between modification-induced energy changes and silencing activity by analyzing $\Delta\Delta E = \Delta E_{mod} - \Delta E_{unmod}$. As shown in Figure S11, unexpectedly, many positions in the seed region exhibit a negative (inverse) correlation with ΔE, although none reach statistical significance. This trend was also present in the MD's energy, which is plotted in Figure S12, but was not explicitly reported in the original study. This observation suggests that positions with locally low energy may resist further stabilization upon chemical modification, potentially attenuating siRNA activity. Given the currently limited sample size, this trend does not yet reach formal statistical significance; therefore, we present this observation as an exploratory finding that warrants further investigation.

It should be noted that comprehensive validation of the two-step structure prediction protocol is currently constrained by the limited number of chemically modified siRNA duplex structures deposited in the Protein DataBank. Importantly, however, the predicted structures serve as intermediate quantities whose primary purpose is to yield physically meaningful energy descriptors, rather than to reproduce atomic coordinates with crystallographic fidelity. The exceptionally strong correlation between Rosetta-calculated total energy and experimental melting temperature ($r = -0.975$

for parent siRNAs and $r = -0.912$ for modified siRNAs) provides strong indirect evidence that the modeled structures capture the essential thermodynamic properties of the duplex, even if local atomic details deviate from the true structure. As more experimental structures of chemically modified siRNAs become available, a more rigorous structural benchmark will be feasible and is an important direction for future validation.

Beyond the need to further improve the energy function, several directions remain for future research. First, although our two-step modified siRNA prediction method combines the efficiency of deep learning with the flexibility of physics-based calculation, the modified backbone still depends on an initial unmodified siRNA structure, which may constrain conformational changes such as sugar pucker.[41] With advances in all-atom structure prediction enhanced by physical force fields,[42,43] it may become possible to predict modified siRNA structures directly. Nevertheless, our current method already provides a foundation for generating synthetic training data to alleviate the scarcity of labeled modified siRNA examples. Second, the lack of large-scale, high-quality modified siRNA efficacy datasets remains a challenge for the field.[13] We acknowledge that the current study relies on a relatively small dataset. However, our method is designed for specific, highly advantageous application scenarios. First, it is primarily intended to evaluate the impact of mature chemical modification patterns on siRNA activity. Second, the framework is best applied to candidate naked sequences that have already undergone preliminary target-specific screening (e.g., incorporating mRNA context), precisely mirroring our wet-lab validation pipeline. Third, the current protocol targets common, clinically relevant modifications (such as 2′-OMe, 2′-F, and PS) that are well supported by Rosetta's parameters; completely unsupported modifications, such as GNA (glycol nucleic acid),[44,45] would require foundational updates to the force field. Within these validated boundaries, our method effectively captures the complex non-linear effects of modifications, providing a robust tool for rational siRNA design. Although we have proposed inference augmentation to improve the generalization of pre-trained tabular deep learning models and have demonstrated the framework's utility on an independent drug design dataset and on two therapeutic targets, generalization performance warrants further evaluation on additional paired datasets and through broader wet-lab experiments. Finally, for practical reasons of throughput and cost, we selected a representative subset of top-ranked siRNA candidates for cell-based validation rather than testing all predicted sequences exhaustively. It must be noted that this focused selection strategy may introduce selection bias, which potentially limits the assessment of the model's ability to rank candidates across the full activity spectrum. In the future, a complete validation experiment incorporating full fixed-chemistry-template and unmodified sequence controls across these specific sequences, alongside the inclusion of lower- and medium-ranked candidates, will provide a more rigorous evaluation of the ranking accuracy of FRAMEs and the incremental benefit of the predictions. For the validated candidates, results were uniformly encouraging; nevertheless, broader experimental coverage—including unmodified parent siRNAs, additional modification chemistries, and *in vivo* studies—remains an important direction for future work, as does extending the framework to accommodate new modification types as relevant data accrue. In sum, these results show that the proposed framework offers a principled approach to the scarcity of high-quality paired parent-modified siRNA data. Particularly when applied to pre-screened candidate sequences facing mature chemical modifications, physics-based energy features combined with inference augmentation translate into reliable predictions in a real-world siRNA design setting.

In summary, we present a validated, structure-based computational framework for the rational design of chemically modified siRNAs. By integrating a deep-learning method for structure prediction, Rosetta for modification refinement and energy-based feature extraction, we introduce the first static, non-MD-based approach to modified siRNA structure prediction, made accessible through a user-friendly web interface. We further propose an inference-augmented tabular deep-learning strategy tailored to data-scarce and noisy biological settings. Our pipeline delivers fast and accurate IC50 estimates without the computational overhead of MD simulations. The inference augmentation strategy, which leverages physical energy-calculation replicates as in-context training data, is key to robust performance under small-data conditions. Two complementary cross-validation protocols—LOOCV and stratified 5-fold CV—consistently identified inference-augmented TabPFN as the top-performing model, with both schemes producing mutually reinforcing evidence for the benefit of real-world physical replicates as augmentation data. Crucially, evaluation on an independent external test set derived from the FDA-approved drug Givosiran demonstrated that FRAMEs generalizes effectively to out-of-distribution data, outperforming ENsiRNA-mod and underscoring the OOD potential of physics-based features combined with inference augmentation. Importantly, wet-lab experimental validation confirmed the reliability of our computational predictions, with the predicted high-activity candidates demonstrating strong gene-silencing effects in cell-based assays. This experimental validation underscores the practical utility of our framework for guiding rational siRNA modification design.

More broadly, the inference augmentation strategy addresses a pervasive challenge in biomedical machine learning—data scarcity and measurement noise—and may generalize to tabular regression tasks across biology and medicine. Furthermore, the findings of this study chart clear directions for future work, including the refinement of the all-atom Rosetta scoring function, and the next generation of deep learning models aimed at direct modified siRNA structure prediction. Together, these advances are expected to further close the gap between computational prediction and experimental practice, ultimately accelerating the rational design and clinical translation of siRNA therapeutics.

MATERIALS AND METHODS

Data source

As aforementioned, previous modified siRNA datasets such as siRNAmod lack paired parent and modified sequences with modification patterns representative of current clinical practice.[46] We therefore

utilized a recently published dataset of chemically modified siRNAs,[10] which provides experimentally measured IC50 values, melting temperatures (T_m), and MD-derived structural descriptors. The dataset includes both parent (unmodified) and modified sequences, specifically comprising 15 unmodified and 15 modified sequences, enabling direct comparison of modification effects; modification patterns are representative of current clinical applications. For the comparison with the MD study, IC50 values were log-transformed ($\log_e(IC_{50})$) to align with their methodology; for our predictive modeling, a base-10 logarithm ($\log_{10}(IC_{50})$) was applied to align with the medicinal chemistry standard (pIC50) and to normalize the inherently large dynamic range of experimental values, thereby improving numerical stability during model training. Because overhang nucleotides have been shown to contribute less to silencing activity and to hinder generalization to sequences of varying lengths,[11,47] overhang features were excluded from model training while being retained for structure prediction to maintain physical accuracy.

Structure prediction and energy calculation

Initial 3D structures of chemically modified siRNA duplexes were generated using Protenix,[16] a protein and nucleic-acid structure-prediction tool that serves as an open-source counterpart to AlphaFold3.[15] It employs a diffusion-based architecture to generate 3D atomic coordinates from structural representations processed by a Pairformer network. Protenix achieves performance comparable to AlphaFold3 while surpassing other deep-learning methods in accuracy on nucleic-acid structures. Previous benchmarks have shown that AlphaFold3 outperforms most existing solutions in RNA folding, especially compared with ab initio methods, while maintaining faster inference speeds.[17]

To ensure a fair and rigorous evaluation of duplex modeling, we built a new duplex nucleic acid benchmark comprising recently released structures, including PDB: 23JY, 8TQX, 8VFS, 9I8B, 9IF1, 9KAD, 9VSN, and 9VUN. All evaluated methods generated 5 structural samples using their default parameters without relying on multiple sequence alignments (MSA). The LDDT and TM-score metrics were calculated using OpenStructure,[48] consistent with the FoldBench evaluation protocol.[21] For candidate selection, deep learning models were assessed using their default ranking or confidence scores, whereas FARFAR2 relied on its output REU. As demonstrated by previous reports and our own duplex strand benchmark, Protenix serves as one of the best open-source tools for RNA structure prediction. Notably, it performs exceptionally well even without an MSA database—typically one of the most time-consuming steps in structure prediction—which is crucial for enabling fast, high-throughput screening.

The structures were subsequently energy-minimized using the Rosetta macromolecular modeling suite (RNA_Minimizer protocol using rna_res_level_energy4 with Round = 50 and iter = 10000).[19] Our method leverages Rosetta parameters located in rna_nonnatural. Since existing approaches—such as deep-learning methods or FARFAR2—cannot natively handle structural modeling for fully modified sequences, we propose a two-stage processing scheme: for modifications that do not disrupt the A-form duplex pairing (such as in siRNAs),[22,49] we first predict the unmodified duplex structure and subsequently substitute the modified nucleic acids via mutation.

As demonstrated in protein side-chain refinement, Rosetta is effective at refining local structures with higher accuracy and efficiency than MD simulations.[50,51] We extracted a panel of physicochemical energy terms—including van der Waals interactions, electrostatic interactions, hydrogen bonding, solvation effects, and RNA-specific torsional and geometric constraints—to serve as features for predictive modeling, as detailed in Figure S4. The total score is a weighted sum of physical and statistical potentials, computed using the Rosetta RNA scoring function rna_res_level_energy4.[40,52,53]

Machine learning models

The limited sample size of the dataset constrains the training of deep-learning methods, necessitating compact yet informative feature representations for small-sample learning. To address this challenge, we benchmarked several machine learning algorithms: Ridge Regression, Lasso Regression, Elastic Net, PLS, Random Forest, XGBoost,[54] Linear SVR, and the pre-trained tabular deep learning model TabPFN.[20] For all models except TabPFN, hyperparameters were selected by grid search and implemented using scikit-learn,[55] as detailed in Table S3 in supplemental materials.

TabPFN is a transformer-based foundation model pre-trained on synthetic tabular datasets via in-context learning, which enables accurate predictions on small datasets without task-specific fine-tuning.[20,56] Its architecture employs a bidirectional attention mechanism: each element first attends to all other features within the same sample (row-wise interaction), and then interacts with the corresponding feature across all samples (column-wise interaction). This dual-attention design guarantees permutation invariance with respect to both features and samples, enhances computational efficiency, and enables reliable generalization to tabular dimensions exceeding those encountered during pre-training. Six checkpoints are evaluated in this work: the *default* checkpoint trained solely on synthetic data; the low-skew variant optimized for low-skew targets but with limited average performance; the *real* checkpoint fine-tuned on real data and the best among real-data variants; the *real-variant*, an alternative real-data fine-tuned checkpoint; the small-samples variant with a marginal advantage on datasets smaller than 3,000 samples; and the *variant*, an additional synthetic-data checkpoint. All checkpoints are available on the Hugging Face model hub. The *real* checkpoint is used as the baseline for comparison in the following section. Models were evaluated using LOOCV and stratified 5-fold CV, with all replicates of each siRNA assigned to the same fold or the same leave-one-out group to preserve replicate integrity, as described in the following text.

Inference augmentation

Given the stochastic nature of Rosetta energy calculations (arising from the rotamer trials protocol and so on, analogous to the

stochasticity inherent in MD simulations), we performed energy calculations in triplicate to evaluate model robustness and to align with biological replicates. During dataset splitting, all replicates of the same siRNA were assigned to the same cross-validation fold to prevent data leakage and overfitting.

Furthermore, to enhance the performance of pre-trained tabular deep learning models on small, noisy datasets, we propose an augmentation strategy termed inference augmentation, whose procedure is formalized in the following text.

For a given training set of N unique siRNA sequences, let y_i denote the experimental activity label for the i-th sequence. Instead of using a single static feature representation, we leverage R independent energy-calculation replicates to naturally capture physical variations. Let $x_i^{(r)} \in \mathbb{R}^p$ represent the feature vector extracted from the r-th replicate, where $r = 1, \ldots, R$. The inference-augmented training set is constructed by pairing each structural replicate with its corresponding experimental label (Equation 1):

$$\mathcal{D}_{\text{aug}} = \left\{ \left(\mathbf{x}_i^{(r)}, \boldsymbol{y}_i \right) \middle| \boldsymbol{i} = \mathbf{1}, \ldots, \boldsymbol{N};\ \boldsymbol{r} = \mathbf{1}, \ldots, \boldsymbol{R}, \right\} \qquad \text{(Equation 1)}$$

In our experiments, energy minimizations were performed in triplicate ($R = 3$), effectively tripling the dataset size and naturally incorporating physically meaningful structural stochasticity into the model.

To contextualize real-data inference augmentation, we considered two complementary baselines: one based on continued pre-training with real-world data,[57] and the other using synthetic data for augmentation.[58] TabPFN-real leverages broader, potentially noisier corpora such as CommonCrawl or GitTables to continue pre-training the foundation model, which was originally pre-trained solely on synthetic data. In addition to TabPFN-real, we compared real-world inference augmentation against augmentation with synthetic Gaussian noise ($\sigma = 0.5, 0.1, 0.05$, and 0.01) added to the original features. The synthetic augmentation procedure is defined as follows. Given a training set $\left\{ \left(x_i, y_i \right) \right\}_{i=1}^{N}$, let $\widehat{\sigma}_J = std\left(\left\{ x_{ij} \right\}_{i=1}^{N} \right)$ denote the empirical standard deviation of the j-th feature across all training samples. For each noise level $\alpha \in \{0.5, 0.1, 0.05, 0.01\}$, two synthetic copies are generated per original sample (Equation 2):

$$\mathbf{x}_i^{(k)} = \mathbf{x}_i + \boldsymbol{\varepsilon}_i^{(k)}, \quad \boldsymbol{\varepsilon}_i^{(k)} \sim \mathcal{N}\left(\mathbf{0}, \mathbf{diag}\left(\boldsymbol{\alpha}^2 \widehat{\boldsymbol{\sigma}}_1^2, \ldots, \boldsymbol{\alpha}^2 \widehat{\boldsymbol{\sigma}}_p^2 \right) \right), \quad \boldsymbol{k} = \mathbf{1}, \mathbf{2}, \qquad \text{(Equation 2)}$$

where p is the number of features and the corresponding labels $y_i^{(k)} = y_i$ remain unchanged. The augmented training set thus contains three repeats of each sample $\left\{ \left(x_i, y_i \right), \left(x_i^{(1)}, y_i \right), \left(x_i^{(2)}, y_i \right) \right\}$, mirroring the structure of the real-world augmentation.

Evaluation metrics

Model performance was assessed using the following metrics:

Equation 3: coefficient of determination (R^2):

$$\boldsymbol{R}^2 = \mathbf{1} - \frac{\sum_{i=1}^{n} \left(\boldsymbol{y}_i - \widehat{\boldsymbol{y}}_i \right)^2}{\sum_{i=1}^{n} \left(\boldsymbol{y}_i - \overline{\boldsymbol{y}} \right)^2} \qquad \text{(Equation 3)}$$

Equation 4: mean squared error (MSE):

$$\text{MSE} = \frac{\mathbf{1}}{\boldsymbol{n}} \sum_{i=1}^{n} \left(\boldsymbol{y}_i - \widehat{\boldsymbol{y}}_i \right)^2 \qquad \text{(Equation 4)}$$

Equation 5: Pearson correlation coefficient (r):

$$\boldsymbol{r} = \frac{\sum_{i=1}^{n} \left(\boldsymbol{y}_i - \overline{\boldsymbol{y}} \right) \left(\widehat{\boldsymbol{y}}_i - \overline{\widehat{\boldsymbol{y}}} \right)}{\sqrt{\sum_{i=1}^{n} \left(\boldsymbol{y}_i - \overline{\boldsymbol{y}} \right)^2} \cdot \sqrt{\sum_{i=1}^{n} \left(\widehat{\boldsymbol{y}}_i - \overline{\widehat{\boldsymbol{y}}} \right)^2}} \qquad \text{(Equation 5)}$$

Equation 6: Spearman rank correlation (ρ):

$$\boldsymbol{\rho} = \mathbf{1} - \frac{\mathbf{6} \sum_{i=1}^{n} \boldsymbol{d}_i^2}{\boldsymbol{n}\left(\boldsymbol{n}^2 - \mathbf{1} \right)} \qquad \text{(Equation 6)}$$

where d_i is the difference between the ranks of y_i and $\widehat{y}_i$. Here, y_i denotes the observed $-\log_{10}(\text{IC}_{50})$, $\widehat{y}_i$ the corresponding model prediction, and n the sample size. We selected R^2 as the primary metric to evaluate model performance and guide the selection of models for downstream applications, as it is considered more informative than other metrics for assessing regression models.[59,60]

Development of the web server

To broaden the accessibility of the proposed framework, we implemented a web server integrating the two core components of FRAMEs: the Rosetta-based modification refinement module and the inference-augmented TabPFN prediction pipeline (Figure S14). Users provide a Protenix-folded unmodified siRNA structure together with a chemical modification pattern—either entered manually or selected from a library of clinically representative presets—and the server returns the predicted IC50, pIC50 and total Rosetta energy score. All computations are executed server-side, requiring no local installation of Rosetta or any associated dependencies.

Wet-lab experimental validation

Modified siRNA design and synthesis

The target mRNA RefSeq accession numbers are GenBank: NM_001067.4 (*TOP2A*) and GenBank: NM_001255.3 (*CDC20*). Parent (unmodified) siRNA sequences were first screened by a traditional siRNA design pipeline encompassing efficiency prediction, off-target assessment, and thermodynamic screening.[13,61] Candidate sequences bearing a clinically representative modification pattern—comprising 2′-OMe and 2′-F, and PS modifications—were then scored by our computational framework. Sequences with low predicted IC50 values were selected for experimental verification; their

detailed sequence and modification information are provided in Table 1. The sequences were synthesized by GeneScript with > 95% purity; MASS and HPLC data are provided in supplemental materials (Figures S19–S28).

Cell line and culture

JHH-7 cells were obtained from Procell Life Science & Technology and maintained in DMEM/F12 medium supplemented with 10% fetal bovine serum (FBS) and 1% penicillin-streptomycin at 37°C in a humidified 5% CO_2 atmosphere. Cells were passaged at 80%–90% confluency by treatment with 0.25% trypsin-EDTA for 1–3 min, followed by neutralization with complete medium and centrifugation at 1,200 rpm for 5 min.

Transfection

Candidate siRNAs were transiently transfected into JHH-7 cells using Lipofectamine 3000 Transfection Reagent according to the manufacturer's protocol. Briefly, Lipofectamine 3000 was diluted in Opti-MEM medium; for plasmid DNA, P3000 reagent was additionally included. The siRNA was separately diluted in Opti-MEM, then combined with the diluted Lipofectamine and incubated at room temperature for 15–30 min to form transfection complexes. Complexes were added dropwise to cells in antibiotic-free medium. After 6 h, the medium was replaced with fresh complete medium, and cells were harvested 48 h post-transfection.

RNA extraction

Total RNA was extracted by the Trizol method. Briefly, cells were washed twice with phosphate-buffered saline (PBS) and lysed in 1 mL Trizol reagent. After addition of 200 μL chloroform and vigorous vortexing, the mixture was centrifuged at 12,000 rpm for 10 min at 4C. The aqueous RNA phase was transferred to a fresh tube, precipitated with an equal volume of isopropanol at room temperature for 10 min, and centrifuged at 12,000 rpm for 10 min at 4C. The RNA pellet was washed twice with absolute ethanol (12,000 rpm, 5 min, 4C), air dried for 10 min, dissolved in 20 μL RNase-free ddH_2O, and stored at − 80°C until use. RNA purity and concentration were determined by spectrophotometry.

Reverse transcription

Complementary DNA (cDNA) was synthesized using the Evo M-MLV RT Mix Kit with gDNA Clean for qPCR (Accurate Biology) in a two-step procedure. First, genomic DNA was removed in a 10 μL reaction containing 2 μL gDNA Clean Reaction Mix and 1 μg total RNA (Table S4 in supplemental materials), incubated at 42°C for 2 min. Second, reverse transcription was performed by adding 4 μL 5 × Evo M-MLV RT Reaction Mix and 6 μL RNase-free ddH_2O to the previous 10 μL reaction (Table S5), and incubated at 37C for 15 min followed by 85°C for 5 s. The resulting cDNA was diluted 10-fold before use and stored at − 20°C.

Real-time qPCR

Gene-silencing efficiency was quantified by quantitative real-time PCR (real-time qPCR) using a 20 μL reaction system containing 10 μL 2 × SYBR Green Pro Taq HS Premix, 0.4 μL of each primer (10 μM), 2 μL cDNA template, and RNase-free ddH_2O to 20 μL (Table S6). Primers for *CDC20*, *TOP2A*, and the internal reference gene *GAPDH* are listed in Table S7. Reactions were run on a QuantStudio 1 Real-Time PCR System (Thermo Fisher Scientific) with the following program: 95°C for 30 s (pre-denaturation); 40 cycles of 95°C for 5 s and 60°C for 30 s; followed by melt-curve acquisition. Relative gene expression was calculated by the $\mathbf{2}^{-\Delta\Delta C_t}$ method with GAPDH as the reference gene.

DATA AND CODE AVAILABILITY

All data are available from the corresponding authors upon reasonable request and included in the main text and supplemental material.

ACKNOWLEDGMENTS

This work was supported by the National Key R&D Program of China (2024YFF1206603 and 2018YFC0910201) and the Key R&D Program of Guangdong Province (2019B020226001).

AUTHOR CONTRIBUTIONS

W.T. developed the computational methods, designed and performed method validation, built the web platform, and wrote the manuscript. Y.D. contributed to method validation. Y.S., N.C., and S.Y. performed wet-lab validation. H.Z. contributed to web platform development. P.C. and Y.Z. contributed to manuscript revision. H.D. conceptualized the study, supervised the project, and contributed to manuscript revision.

DECLARATION OF INTERESTS

The authors declare that they have no known competing financial interests or personal relationships that could have appeared to influence the work reported in this paper.

SUPPLEMENTAL INFORMATION

Supplemental information can be found online at https://doi.org/10.1016/j.omtn.2026.103029.

REFERENCES

1. Almeida, R., and Allshire, R.C. (2005). RNA silencing and genome regulation. Trends Cell Biol. *15*, 251–258. https://doi.org/10.1016/j.tcb.2005.03.006.
2. Ketting, R.F., Fischer, S.E.J., Bernstein, E., Sijen, T., Hannon, G.J., and Plasterk, R.H.A. (2001). Dicer functions in RNA interference and in synthesis of small RNA involved in developmental timing in C. elegans. Genes Dev. *15*, 2654–2659. https://doi.org/10.1101/gad.927801.
3. Dana, H., Chalbatani, G.M., Mahmoodzadeh, H., Gharagouzlo, E., Karimloo, R., Rezaiean, O., Moradzadeh, A., Mazraeh, A., Marmari, V., Rashno, M.M., et al. (2017). Molecular mechanisms and biological functions of siRNA. Int. J. Biomed. Sci. *13*, 48–57. https://doi.org/10.59566/ijbs.2017.13048.
4. Guo, S., Zhang, M., and Huang, Y. (2024). Three 'E'challenges for siRNA drug development. Trends in molecular medicine *30*, 13–24. https://doi.org/10.1016/j.molmed.2023.10.005.
5. Xiao, B., Wang, S., Pan, Y., Zhi, W., Gu, C., Guo, T., Zhai, J., Li, C., Chen, Y.Q., and Wang, R. (2025). Development, opportunities, and challenges of siRNA nucleic acid drugs. Mol. Ther. Nucleic Acids *36*, 102437. https://doi.org/10.1016/j.omtn.2024.102437.
6. Hu, B., Zhong, L., Weng, Y., Peng, L., Huang, Y., Zhao, Y., and Liang, X.-J. (2020). Therapeutic siRNA: state of the art. Signal Transduct. Targeted Ther. *5*, 101. https://doi.org/10.1038/s41392-020-0207-x.
7. Allerson, C.R., Sioufi, N., Jarres, R., Prakash, T.P., Naik, N., Berdeja, A., Wanders, L., Griffey, R.H., Swayze, E.E., and Bhat, B. (2005). Fully 2'-Modified Oligonucleotide Duplexes with Improved in Vitro Potency and Stability Compared to Unmodified Small Interfering RNA. J. Med. Chem. *48*, 901–904. https://doi.org/10.1021/jm049167j.

8. Nair, J.K., Willoughby, J.L.S., Chan, A., Charisse, K., Alam, M.R., Wang, Q., Hoekstra, M., Kandasamy, P., Kel'in, A.V., Milstein, S., et al. (2014). Multivalent *N* -Acetylgalactosamine-Conjugated siRNA Localizes in Hepatocytes and Elicits Robust RNAi-Mediated Gene Silencing. J. Am. Chem. Soc. *136*, 16958–16961. https://doi.org/10.1021/ja505986a.

9. Foster, D.J., Brown, C.R., Shaikh, S., Trapp, C., Schlegel, M.K., Qian, K., Sehgal, A., Rajeev, K.G., Jadhav, V., Manoharan, M., et al. (2018). Advanced siRNA Designs Further Improve In Vivo Performance of GalNAc-siRNA Conjugates. Mol. Ther. *26*, 708–717. https://doi.org/10.1016/j.ymthe.2017.12.021.

10. Kliuchnikov, E., Maksudov, F., Zuber, J., Hyde, S., Castoreno, A., Waldron, S., Schlegel, M.K., Marx, K.A., Maier, M.A., and Barsegov, V. (2025). Improving the potency prediction for chemically modified siRNAs through insights from molecular modeling of individual sequence positions. Mol. Ther. Nucleic Acids *36*, 102415. https://doi.org/10.1016/j.omtn.2024.102415.

11. Davis, S.M., Hildebrand, S., MacMillan, H.J., Monopoli, K.R., Buchwald, J., Sousa, J., Cooper, D., Ly, S., Echeverria, D., McHugh, N., et al. (2025). Systematic analysis of siRNA and mRNA features impacting fully chemically modified siRNA efficacy. Nucleic Acids Res. *53*, gkaf479. https://doi.org/10.1093/nar/gkaf479.

12. Liu, T., Huang, J., Luo, D., Ren, L., Ning, L., Huang, J., Lin, H., and Zhang, Y. (2024). Cm-siRPred: Predicting chemically modified siRNA efficiency based on multi-view learning strategy. Int. J. Biol. Macromol. *264*, 130638. https://doi.org/10.1016/j.ijbiomac.2024.130638.

13. Tan, W., Dai, M., Ye, S., Tang, X., Jiang, D., Chen, D., and Du, H. (2025). ENsiRNA: a multimodality method for siRNA-mRNA and modified siRNA efficacy prediction based on geometric graph neural network. J. Mol. Biol. *437*, 169131. https://doi.org/10.1016/j.jmb.2025.169131.

14. Wei, H., and McCammon, J.A. (2024). Structure and dynamics in drug discovery. npj Drug Discov. *1*, 1. https://doi.org/10.1038/s44386-024-00001-2.

15. Abramson, J., Adler, J., Dunger, J., Evans, R., Green, T., Pritzel, A., Ronneberger, O., Willmore, L., Ballard, A.J., Bambrick, J., et al. (2024). Accurate structure prediction of biomolecular interactions with AlphaFold 3. Nature *630*, 493–500. https://doi.org/10.1038/s41586-024-07487-w.

16. Team, B.A.A., Chen, X., Zhang, Y., Lu, C., Ma, W., Guan, J., Gong, C., Yang, J., Zhang, H., and Zhang, K. (2025). Protenix-advancing structure prediction through a comprehensive AlphaFold3 reproduction. Preprint at bioRxiv. https://doi.org/10.1101/2025.01.08.631967.

17. Bernard, C., Postic, G., Ghannay, S., and Tahi, F. (2025). Has AlphaFold3 achieved success for RNA? Acta Crystallogr. D Struct. Biol. *81*, 49–62. https://doi.org/10.1107/S2059798325000592.

18. Cao, S., Zhang, C., Zhu, N., Li, C., Mao, Q., Cao, Z., Ge, Y., Wu, Y., Guo, J., Cao, Q., et al. (2026). HighRelax: Physics-Based Refinement of Deep Learning Protein Predictions with Noncanonical Amino Acids. J. Chem. Theor. Comput. *22*, 3093–3102. https://doi.org/10.1021/acs.jctc.5c01807.

19. Leman, J.K., Weitzner, B.D., Lewis, S.M., Adolf-Bryfogle, J., Alam, N., Alford, R.F., Aprahamian, M., Baker, D., Barlow, K.A., Barth, P., et al. (2020). Macromolecular modeling and design in Rosetta: recent methods and frameworks. Nat. Methods *17*, 665–680. https://doi.org/10.1038/s41592-020-0848-2.

20. Hollmann, N., Müller, S., Purucker, L., Krishnakumar, A., Körfer, M., Hoo, S.B., Schirrmeister, R.T., and Hutter, F. (2025). Accurate predictions on small data with a tabular foundation model. Nature *637*, 319–326. https://doi.org/10.1038/s41586-024-08328-6.

21. Xu, S., Feng, Q., Qiao, L., Wu, H., Shen, T., Cheng, Y., Zheng, S., and Sun, S. (2025). Benchmarking all-atom biomolecular structure prediction with FoldBench. Nat. Commun. *17*, 442. https://doi.org/10.1038/s41467-025-67127-3.

22. Podbevsek, P., Allerson, C.R., Bhat, B., and Plavec, J. (2010). Solution-state structure of a fully alternately 2′-F/2′-OMe modified 42-nt dimeric siRNA construct. Nucleic Acids Res. *38*, 7298–7307. https://doi.org/10.1093/nar/gkq621.

23. Passaro, S., Corso, G., Wohlwend, J., Reveiz, M., Thaler, S., Somnath, V.R., Getz, N., Portnoi, T., Roy, J., and Stark, H. (2025). Boltz-2: Towards accurate and efficient binding affinity prediction. Preprint at bioRxiv. https://doi.org/10.1101/2025.06.14.659707.

24. The OpenFold3 Team (2025). OpenFold3-preview Version 0.4.1. https://doi.org/10.5281/zenodo.19001000.

25. team, C.D., Boitreaud, J., Dent, J., McPartlon, M., Meier, J., Reis, V., Rogozhonikov, A., and Wu, K. (2024). Chai-1: Decoding the molecular interactions of life. Preprint at bioRxiv. https://doi.org/10.1101/2024.10.10.615955.

26. Steiger, J.H. (1980). Tests for comparing elements of a correlation matrix. Psychol. Bull. *87*, 245–251. https://doi.org/10.1037/0033-2909.87.2.245.

27. Li, J., Gao, J.-Z., Du, J.-L., Huang, Z.-X., and Wei, L.-X. (2014). Increased CDC20 expression is associated with development and progression of hepatocellular carcinoma. Int. J. Oncol. *45*, 1547–1555. https://doi.org/10.3892/ijo.2014.2559.

28. Yang, G., Wang, G., Xiong, Y., Sun, J., Li, W., Tang, T., and Li, J. (2021). CDC20 promotes the progression of hepatocellular carcinoma by regulating epithelial-mesenchymal transition. Mol. Med. Rep. *24*, 483. https://doi.org/10.3892/mmr.2021.12122.

29. Zhao, S., Zhang, Y., Lu, X., Ding, H., Han, B., Song, X., Miao, H., Cui, X., Wei, S., Liu, W., et al. (2021). CDC20 regulates the cell proliferation and radiosensitivity of P53 mutant HCC cells through the Bcl-2/Bax pathway. Int. J. Biol. Sci. *17*, 3608–3621. https://doi.org/10.7150/ijbs.64003.

30. Pommier, Y., Sun, Y., Huang, S.y.N., and Nitiss, J.L. (2016). Roles of eukaryotic topoisomerases in transcription, replication and genomic stability. Nat. Rev. Mol. Cell Biol. *17*, 703–721. https://doi.org/10.1038/nrm.2016.111.

31. Wang, T., Lu, J., Wang, R., Cao, W., and Xu, J. (2022). TOP2A promotes proliferation and metastasis of hepatocellular carcinoma regulated by miR-144-3p. J. Cancer *13*, 589–601. https://doi.org/10.7150/jca.64017.

32. Meng, J., Wei, Y., Deng, Q., Li, L., and Li, X. (2022). Study on the expression of TOP2A in hepatocellular carcinoma and its relationship with patient prognosis. Cancer Cell Int. *22*, 29. https://doi.org/10.1186/s12935-021-02439-0.

33. Wang, Z., Zhu, Q., Li, X., Ren, X., Li, J., Zhang, Y., Zeng, S., Xu, L., Dong, X., and Zhai, B. (2022). TOP2A inhibition reverses drug resistance of hepatocellular carcinoma to regorafenib. Am. J. Cancer Res. *12*, 4343.

34. Lundberg, S.M., and Lee, S.-I. (2017). A Unified Approach to Interpreting Model Predictions. In Advances in Neural Information Processing Systems, I. Guyon, U. von Luxburg, S. Bengio, H. Wallach, R. Fergus, S. Vishwanathan, and R. Garnett, eds. (Curran Associates, Inc).

35. Fan, J., Han, F., and Liu, H. (2014). Challenges of big data analysis. Natl. Sci. Rev. *1*, 293–314. https://doi.org/10.1093/nsr/nwt032.

36. Li, Y., Yang, J., Xiao, P., Liu, H., Zhou, Y., Yang, X., Chen, G., and Zuo, Z. (2025). MRI Delta-Radiomics and Morphological Feature-Driven TabPFN Model for Preoperative Prediction of Lymphovascular Invasion in Invasive Breast Cancer. Technol. Cancer Res. Treat. *24*, 15330338251362050. https://doi.org/10.1177/15330338251362050.

37. Saber, R., Carneiro, M., Montagnon, E., Tang, A., Turcotte, S., and Kadoury, S. (2026). Multi-omics fusion network for prediction of early recurrence in colorectal liver metastases. npj Precis. Onc. *10*, 61. https://doi.org/10.1038/s41698-025-01264-2.

38. Wu, D.-N., Jen, J., Fajiculay, E., Hsu, M.-F., Chang, M.-C., Yeh, J.-C., Sargsyan, K., Kupcinskas, J., Skieceviciene, J., Steponaitiene, R., et al. (2026). PanMETAI - a high performance tabular foundation model for accurate pancreatic cancer diagnosis via NMR metabolomics. Nat. Commun. *17*, 1595. https://doi.org/10.1038/s41467-026-69426-9.

39. Tsurumi, A., Cahill, C.M., Liu, A.J., Chatterjee, P., Das, S., and Kobayashi, A. (2025). Development of Plasma Protein Classification Models for Alzheimer's Disease Using Multiple Machine Learning Approaches. Int. J. Mol. Sci. *26*, 11673. https://doi.org/10.3390/ijms262311673.

40. Watkins, A.M., Geniesse, C., Kladwang, W., Zakrevsky, P., Jaeger, L., and Das, R. (2018). Blind prediction of noncanonical RNA structure at atomic accuracy. Sci. Adv. *4*, eaar5316. https://doi.org/10.1126/sciadv.aar5316.

41. Venkateswarlu, D., Lind, K.E., Mohan, V., Manoharan, M., and Ferguson, D.M. (1999). Structural properties of DNA:RNA duplexes containing 2'-O-methyl and 2'-S-methyl substitutions: a molecular dynamics investigation. Nucleic Acids Res. *27*, 2189–2195. https://doi.org/10.1093/nar/27.10.2189.

42. Passaro, S., Corso, G., Wohlwend, J., Reveiz, M., Thaler, S., Somnath, V.R., Getz, N., Portnoi, T., Roy, J., Stark, H., et al. (2025). Boltz-2: Towards Accurate and Efficient Binding Affinity Prediction. Preprint at bioRxiv *2025*, 659707. https://doi.org/10.1101/2025.06.14.659707.

43. Lewis, S., Hempel, T., Jiménez-Luna, J., Gastegger, M., Xie, Y., Foong, A.Y.K., Satorras, V.G., Abdin, O., Veeling, B.S., Zaporozhets, I., et al. (2025). Scalable emulation of protein equilibrium ensembles with generative deep learning. Science *389*, eadv9817. https://doi.org/10.1126/science.adv9817.

44. Schlegel, M.K., Matsuda, S., Brown, C.R., Harp, J.M., Barry, J.D., Berman, D., Castoreno, A., Schofield, S., Szeto, J., Manoharan, M., et al. (2021). Overcoming GNA/RNA base-pairing limitations using isonucleotides improves the pharmacodynamic activity of ESC+ GalNAc-siRNAs. Nucleic Acids Res. *49*, 10851–10867. https://doi.org/10.1093/nar/gkab916.

45. Egli, M., Schlegel, M.K., and Manoharan, M. (2023). Acyclic (S)-glycol nucleic acid (S-GNA) modification of siRNAs improves the safety of RNAi therapeutics while maintaining potency. Rna *29*, 402–414. https://doi.org/10.1261/rna.079526.122.

46. Dar, S.A., Thakur, A., Qureshi, A., and Kumar, M. (2016). siRNAmod: a database of experimentally validated chemically modified siRNAs. Sci. Rep. *6*, 20031. https://doi.org/10.1038/srep20031.

47. Alshaer, W., Zureigat, H., Al Karaki, A., Al-Kadash, A., Gharaibeh, L., Ma'mon, M.H., Aljabali, A.A.A., and Awidi, A. (2021). siRNA: Mechanism of action, challenges, and therapeutic approaches. Eur. J. Pharmacol. *905*, 174178. https://doi.org/10.1016/j.ejphar.2021.174178.

48. Biasini, M., Schmidt, T., Bienert, S., Mariani, V., Studer, G., Haas, J., Johner, N., Schenk, A.D., Philippsen, A., and Schwede, T. (2013). OpenStructure: an integrated software framework for computational structural biology. Biological crystallography *69*, 701–709. https://doi.org/10.1107/s0907444913007051.

49. Manoharan, M., Akinc, A., Pandey, R.K., Qin, J., Hadwiger, P., John, M., Mills, K., Charisse, K., Maier, M.A., Nechev, L., et al. (2011). Unique Gene-Silencing and Structural Properties of 2′-Fluoro-Modified siRNAs. Angew. Chem. *123*, 2332–2336. https://doi.org/10.1002/ange.201006519.

50. Tyka, M.D., Keedy, D.A., André, I., Dimaio, F., Song, Y., Richardson, D.C., Richardson, J.S., and Baker, D. (2011). Alternate states of proteins revealed by detailed energy landscape mapping. J. Mol. Biol. *405*, 607–618. https://doi.org/10.1016/j.jmb.2010.11.008.

51. Lindert, S., and McCammon, J.A. (2015). Improved cryoEM-Guided Iterative Molecular Dynamics–Rosetta Protein Structure Refinement Protocol for High Precision Protein Structure Prediction. J. Chem. Theor. Comput. *11*, 1337. https://doi.org/10.1021/ct500995d.

52. Watkins, A.M., Rangan, R., and Das, R. (2020). FARFAR2: improved de novo rosetta prediction of complex global RNA folds. Structure *28*, 963–976.e6. https://doi.org/10.1016/j.str.2020.05.011.

53. Watkins, A.M., Rangan, R., and Das, R. (2019). Using Rosetta for RNA homology modeling. Methods Enzymol. *623*, 177–207. https://doi.org/10.1016/bs.mie.2019.05.026.

54. Chen, T., and Guestrin, C. (2016). Xgboost: A scalable tree boosting system. Preprint at arXiv. https://doi.org/10.48550/arXiv.1603.02754.

55. Pedregosa, F., Varoquaux, G., Gramfort, A., Michel, V., Thirion, B., Grisel, O., Blondel, M., Prettenhofer, P., Weiss, R., and Dubourg, V. (2011). Scikit-learn: Machine learning in Python. J. Mach. Learn. Res. *12*, 2825–2830.

56. Vaswani, A., Shazeer, N., Parmar, N., Uszkoreit, J., Jones, L., Gomez, A.N., Kaiser, Ł., and Polosukhin, I. (2017). Attention is all you need. Advances in neural information processing systems *30*.

57. Garg, A., Ali, M., Hollmann, N., Purucker, L., Müller, S., and Hutter, F. (2025). Real-TabPFN: Improving Tabular Foundation Models via Continued Pre-training With Real-World Data. Preprint at arXiv. https://doi.org/10.48550/arXiv.2507.03971.

58. Bühler, M., Purucker, L., and Hutter, F. (2026). Causal Data Augmentation for Robust Fine-Tuning of Tabular Foundation Models. Preprint at arXiv. https://doi.org/10.48550/arXiv.2601.04110.

59. Chicco, D., Warrens, M.J., and Jurman, G. (2021). The coefficient of determination R-squared is more informative than SMAPE, MAE, MAPE, MSE and RMSE in regression analysis evaluation. PeerJ Comput. Sci. *7*, e623. https://doi.org/10.7717/peerj-cs.623.

60. Willmott, C.J. (1981). On the Validation of Models. Phys. Geogr. *2*, 184–194. https://doi.org/10.1080/02723646.1981.10642213.

61. Mysara, M., Garibaldi, J.M., and ElHefnawi, M. (2011). MysiRNA-designer: a workflow for efficient siRNA design. PLoS One *6*, e25642. https://doi.org/10.1371/journal.pone.0025642.

OMTN, Volume *37*

Supplemental information

Fast activity prediction of chemically modified siRNAs via structure-based energy calculations and inference-augmented tabular deep learning

Wenchong Tan, Yiheng Dong, Yuanfang Shi, Nanwen Chen, Shimin Ye, Heng Zhang, Ping Chen, Yinglin Zuo, and Hongli Du

Supplemental Results

To pinpoint the origin of the performance gains achieved via Inference Augmentation under leave-one-out cross-validation (LOOCV), we algebraically decomposed the R^2 metric. This analysis reveals that the substantial increase in R^2 stems primarily from improved prediction calibration, rather than solely from enhanced ranking correlation over strong baselines.

Using the standard coefficient of determination, $R^2 = 1 - \mathrm{SS}_{\mathrm{res}}/\mathrm{SS}_{\mathrm{tot}}$, let y and $\hat{y}$ denote the vectors of experimental and predicted values, respectively. The mean squared error (MSE) expands as Equation S1:

$$\mathrm{MSE} = \frac{1}{N}\sum_{i=1}^{N}(y_i - \hat{y}_i)^2 = \sigma_{\hat{y}}^2 + \sigma_y^2 - 2\mathrm{Cov}(y,\hat{y}) + (\bar{\hat{y}} - \bar{y})^2$$

Dividing by the variance of the experimental values σ_y^2, we obtain Equation S2:

$$\frac{\mathrm{MSE}}{\sigma_y^2} = \left(\frac{\sigma_{\hat{y}}}{\sigma_y}\right)^2 + 1 - 2\left(\frac{\mathrm{Cov}(y,\hat{y})}{\sigma_y\sigma_{\hat{y}}}\right)\left(\frac{\sigma_{\hat{y}}}{\sigma_y}\right) + \left(\frac{\bar{\hat{y}} - \bar{y}}{\sigma_y}\right)^2$$

We define three interpretable components: (1) the Pearson correlation coefficient $r = \mathrm{Cov}(y,\hat{y})/(\sigma_y\sigma_{\hat{y}})$; (2) the variance matching factor (ratio of standard deviations) $k = \sigma_{\hat{y}}/\sigma_y$; and (3) the normalized mean bias $b = (\bar{\hat{y}} - \bar{y})/\sigma_y$. Substituting these into $R^2 = 1 - \mathrm{MSE}/\sigma_y^2$ yields Equation S3:

$$R^2 = 1 - (k^2 + 1 - 2rk + b^2) = 2rk - k^2 - b^2$$

While numerically identical to the standard definition, this formulation isolates the contributions of linear correlation (r), variance matching (k), and mean bias (b).[1,2] It clarifies why R^2 can improve far more than Pearson r when augmentation primarily corrects calibration. Specifically, Figure S15A details these components for TabPFN without augmentation ($R^2 \approx 0.09$, $r \approx 0.35$), PLS without augmentation ($R^2 \approx 0.40$, $r \approx 0.64$), and TabPFN with augmentation ($R^2 \approx 0.54$, $r \approx 0.74$). Without augmentation, TabPFN severely underestimates variance ($k \ll 1$), which penalizes its R^2 despite moderate correlation. Supplemental Figures S15D and S15F visually confirm that augmentation expands the dynamic range of predictions along the identity line, rather than artificially inflating correlation.

Relative to the PLS baseline (Figure S15E), the augmentation-enabled TabPFN (Figure S15F) improves by $\Delta R^2 \approx +0.14$ and $\Delta r \approx +0.10$. These incremental improvements are consistent with typical gains observed in other domains when measured on correlation or rank scales (e.g., Spearman ρ improving from 0.60 to 0.67, Figure S15C).

Importantly, these gains are not a byproduct of overfitting. Our LOOCV strategy strictly excludes all replicates of the held-out siRNA from the training set. Furthermore, Figure S15B demonstrates heterogeneous, per-siRNA error reductions—a pattern inconsistent with uniform overfitting. Instead, improvements are concentrated in high-leverage siRNAs where independent energy minimization replicates offer complementary structural features. Thus, in $n \ll p$ regimes, inference augmentation effectively leverages physically meaningful stochasticity to improve both calibration and rank accuracy.

To verify that this performance is robust to random initialization, we conducted two sets of independent control experiments (three runs each). The first set randomized the Rosetta energy minimization seeds while fixing the model training seed, whereas the second set fixed the Rosetta seed and randomized the training seeds. As Supplemental Figures S16A-F and S17A-F illustrate, the performance boost is highly stable across both scenarios. On average, the augmented TabPFN model significantly outperformed the baseline under randomized Rosetta seeds (paired t-test: $p = 0.0167$ for R^2, $p = 0.0201$ for Pearson r, and $p = 0.0418$ for Spearman ρ)and under randomized training seeds ($p = 0.0018$ for R^2, $p = 0.0009$ for Pearson r, and $p = 0.0028$ for Spearman ρ).

Finally, we systematically evaluated how the number of independent energy minimization replicates ($n\_repeats$) impacts Inference Augmentation. As shown in Figure S18, scaling $n\_repeats$ reveals a clear saturation point in performance. Under LOOCV, increasing $n\_repeats$ from 1 to 3 dramatically raises R^2 from 0.0902 to 0.5385, Pearson r from 0.3540 to 0.7365, and Spearman ρ from 0.3401 to 0.6678. The external test set follows a similar trend, with Pearson r jumping from 0.7262 to 0.8536. Beyond $n\_repeats = 3$, performance plateaus with only minor fluctuations (e.g., at $n\_repeats = 9$, external test set Pearson $r = 0.8679$). Consequently, $n\_repeats = 3$ offers an optimal balance between computational efficiency and predictive accuracy.

Supplemental Figures

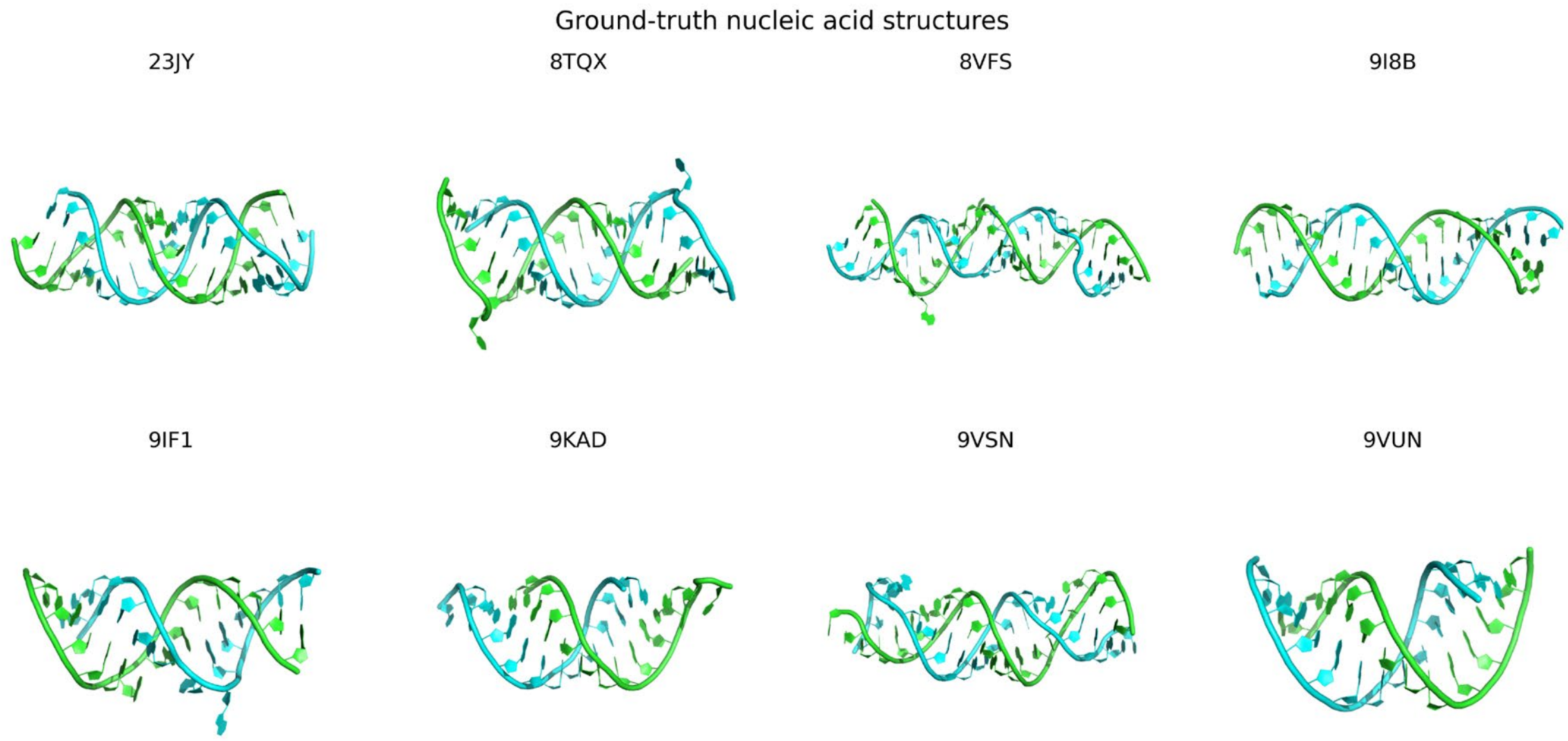

Figure S1. Visualization of structures in the double-stranded nucleic acids benchmark, based on PDB structures released after January 1, 2024.

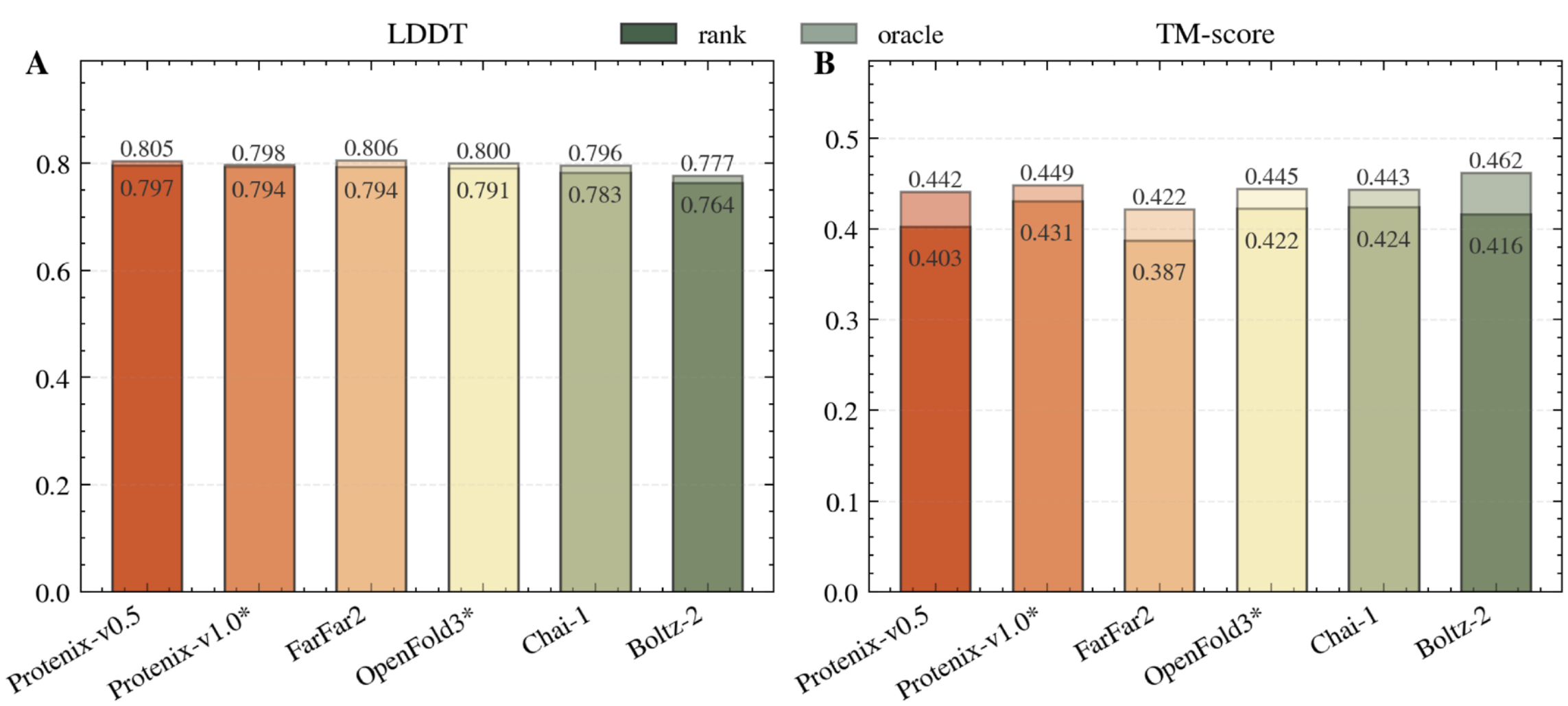

Figure S2. Performance of structure prediction on double-stranded nucleic acids. Bar plots show the average (A) LDDT and (B) TM-score of the predictions, calculated as FoldBench.[3] For each structure, 5 samples were generated; metrics are reported based on the top-ranked sample by the default ranking metric ('rank') and the best possible sample ('oracle'). Asterisks (*) denote the latest released methods included as a complement during the review process.

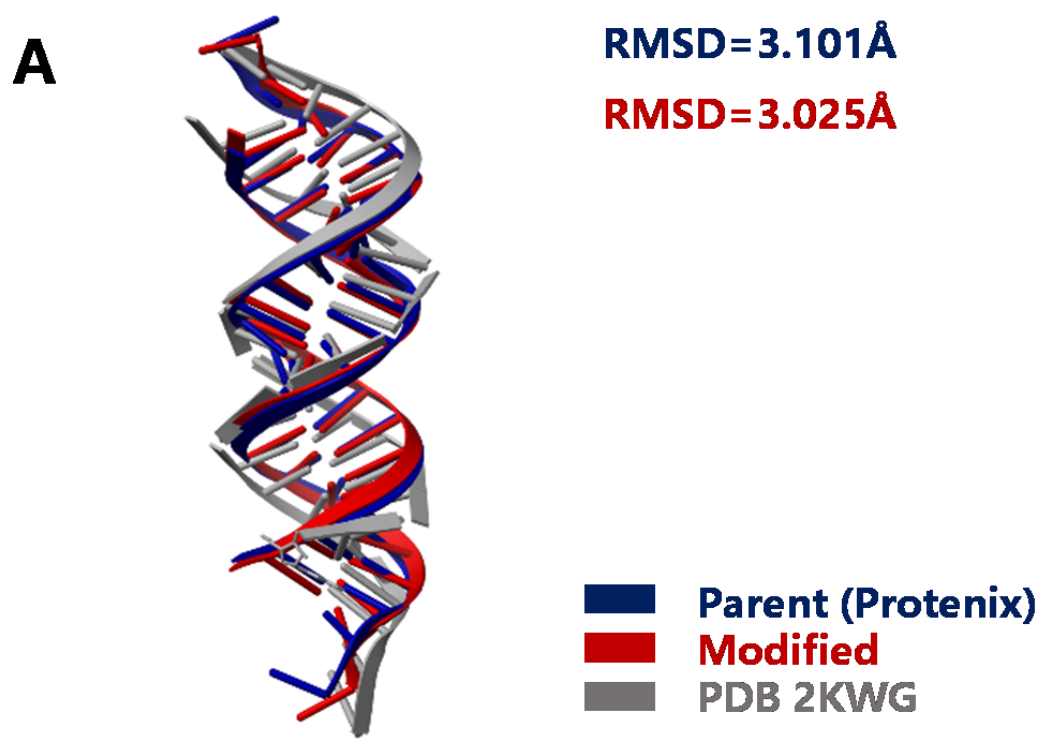

Figure S3. Comparison of the two-step modified siRNA structure prediction pipeline using Protenix versus FARFAR2 as the initial structure prediction tool, evaluated on PDB entry 2KWG (a fully chemically modified siRNA duplex). *(A) Ribbon superimposition of the PDB 2KWG reference structure (grey), the unmodified siRNA predicted by Protenix (blue), and the Rosetta-refined modified siRNA (red). Backbone RMSDs relative to 2KWG are 3.101 Å and 3.025 Å, respectively. (B) As in (A), but using FARFAR2 for initial structure prediction (blue), with backbone RMSDs of 3.849 Å and 4.088 Å, respectively. Lower RMSD values indicate better agreement with the experimental reference structure.*

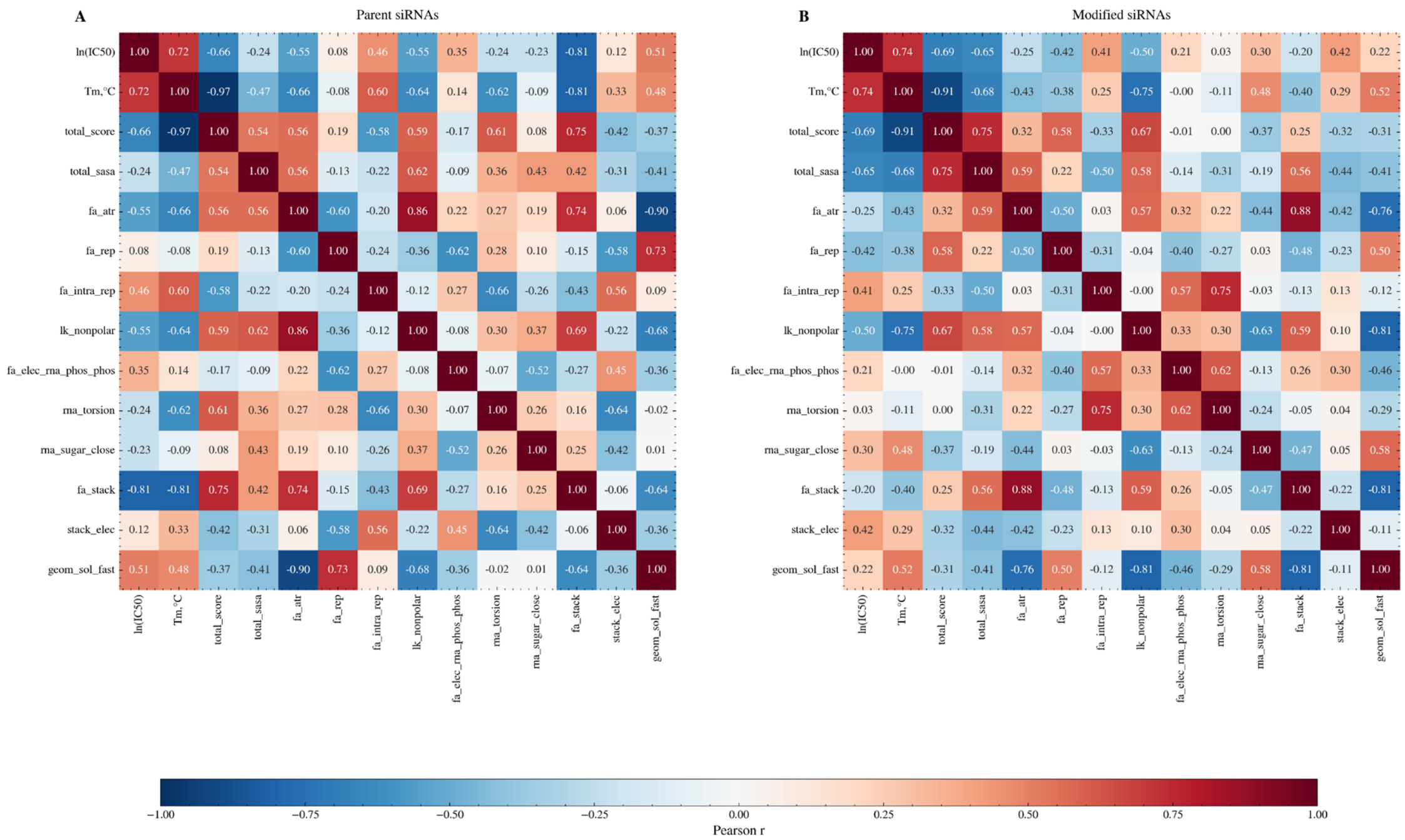

Figure S4. Pearson correlation heatmaps among Rosetta energy terms, $log_e(IC_{50})$, and melting temperature (T_m). *Colour intensity reflects the magnitude of r; red and blue denote positive and negative correlations, respectively. (A) Correlation matrix for parent (unmodified) siRNAs, highlighting the strong negative correlation between total energy score and both T_m and $log_e(IC_{50})$. (B) Correlation matrix for chemically modified siRNAs, showing analogous relationships between total energy score, T_m, and $log_e(IC_{50})$.*

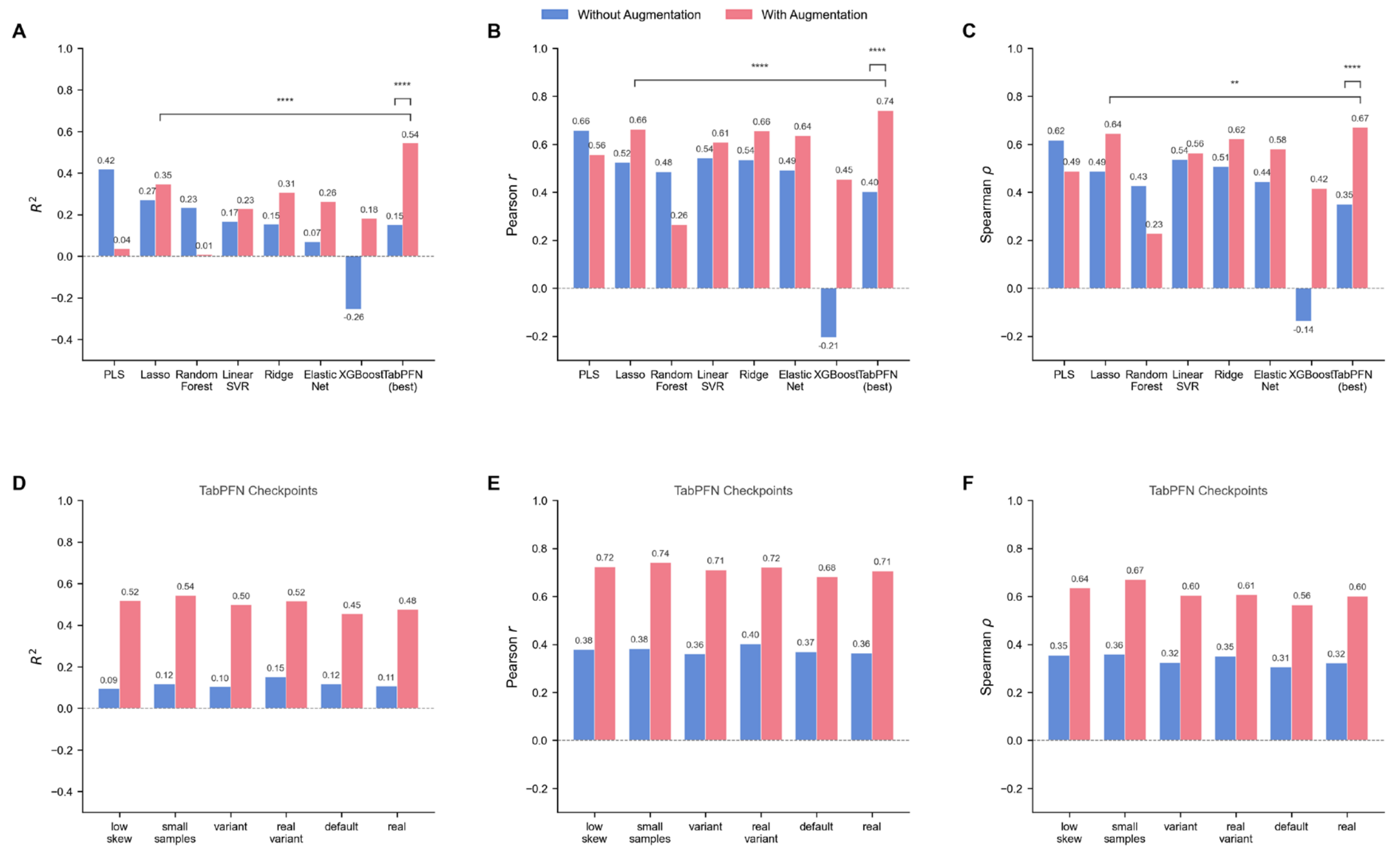

Figure S5. Leave-one-out cross-validation performance on the multi-replicate test set, in which the three energy-calculation replicates of each held-out siRNA are averaged into a single test sample, placing greater emphasis on prediction robustness. *(A–C) Bar charts showing* R^2*, Pearson* r*, and Spearman* ρ*, respectively, for all evaluated models without Inference Augmentation (blue) and with Inference Augmentation (red). "TabPFN (best)" refers to the highest-performing checkpoint. (D–F) Bar charts showing* R^2*, Pearson* r*, and Spearman* ρ*, respectively, for individual TabPFN checkpoints without augmentation (blue) and with Inference Augmentation (red). Significance was assessed by Steiger's Z-test: '****' indicates* $p < 0.001$*; '***'* $p < 0.005$*; '**'* $p < 0.01$*; '*'* $p < 0.05$*.*

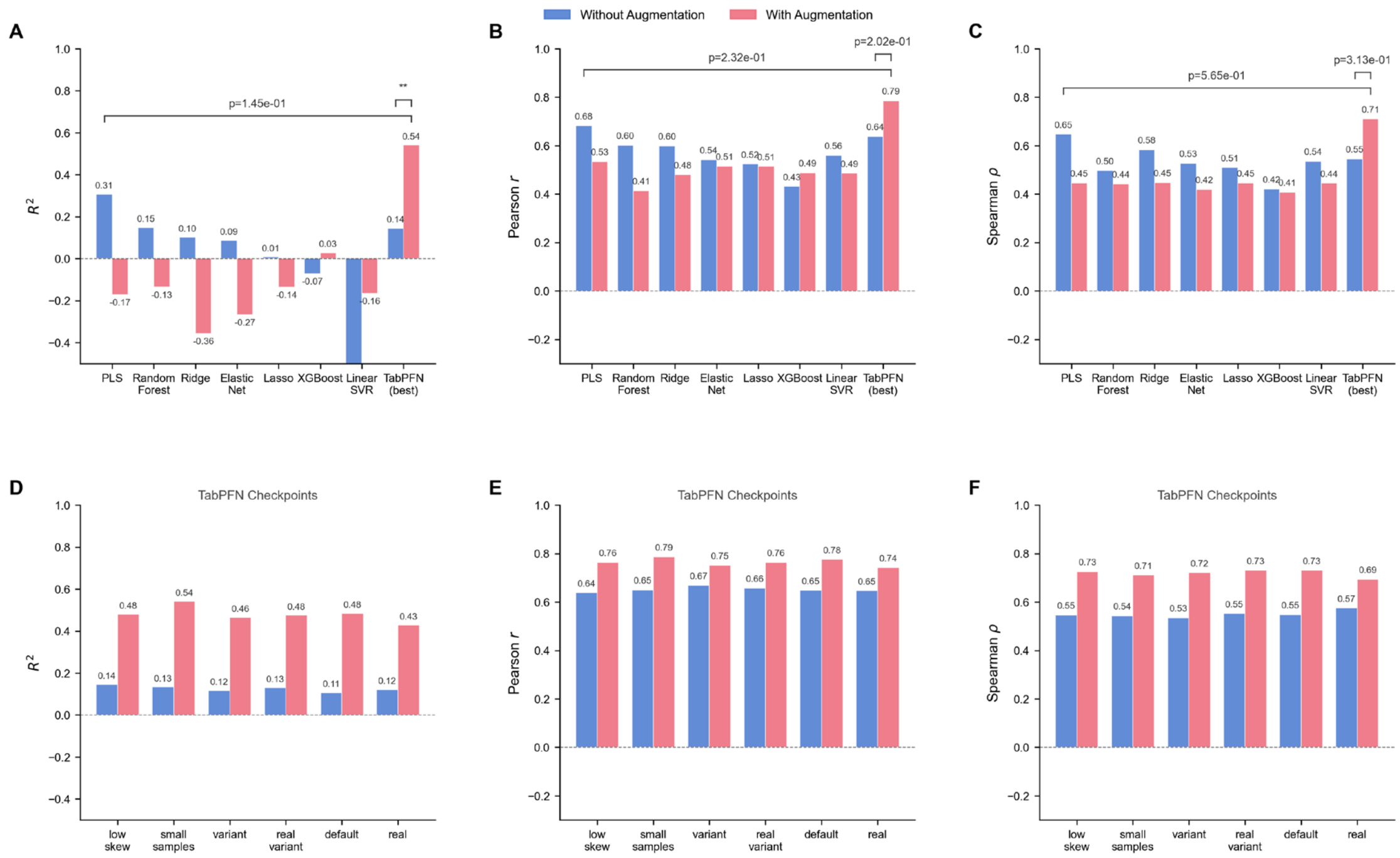

Figure S6. Five-fold cross-validation performance on the multi-replicate test set, in which multiple energy-calculation replicates of the same siRNA may appear within the test set, placing greater demands on model robustness. *(A–C) Bar charts showing* R^2*, Pearson* r*, and Spearman* ρ*, respectively, for all evaluated models without Inference Augmentation (blue) and with Inference Augmentation (red). "TabPFN (best)" refers to the highest-performing checkpoint. (D–F) Bar charts showing* R^2*, Pearson* r*, and Spearman* ρ*, respectively, for individual TabPFN checkpoints without augmentation (blue) and with Inference Augmentation (red). Significance was assessed by paired* t*-test: '****' indicates* $p < 0.001$*; '***'* $p < 0.005$*; '**'* $p < 0.01$*; '*'* $p < 0.05$*.*

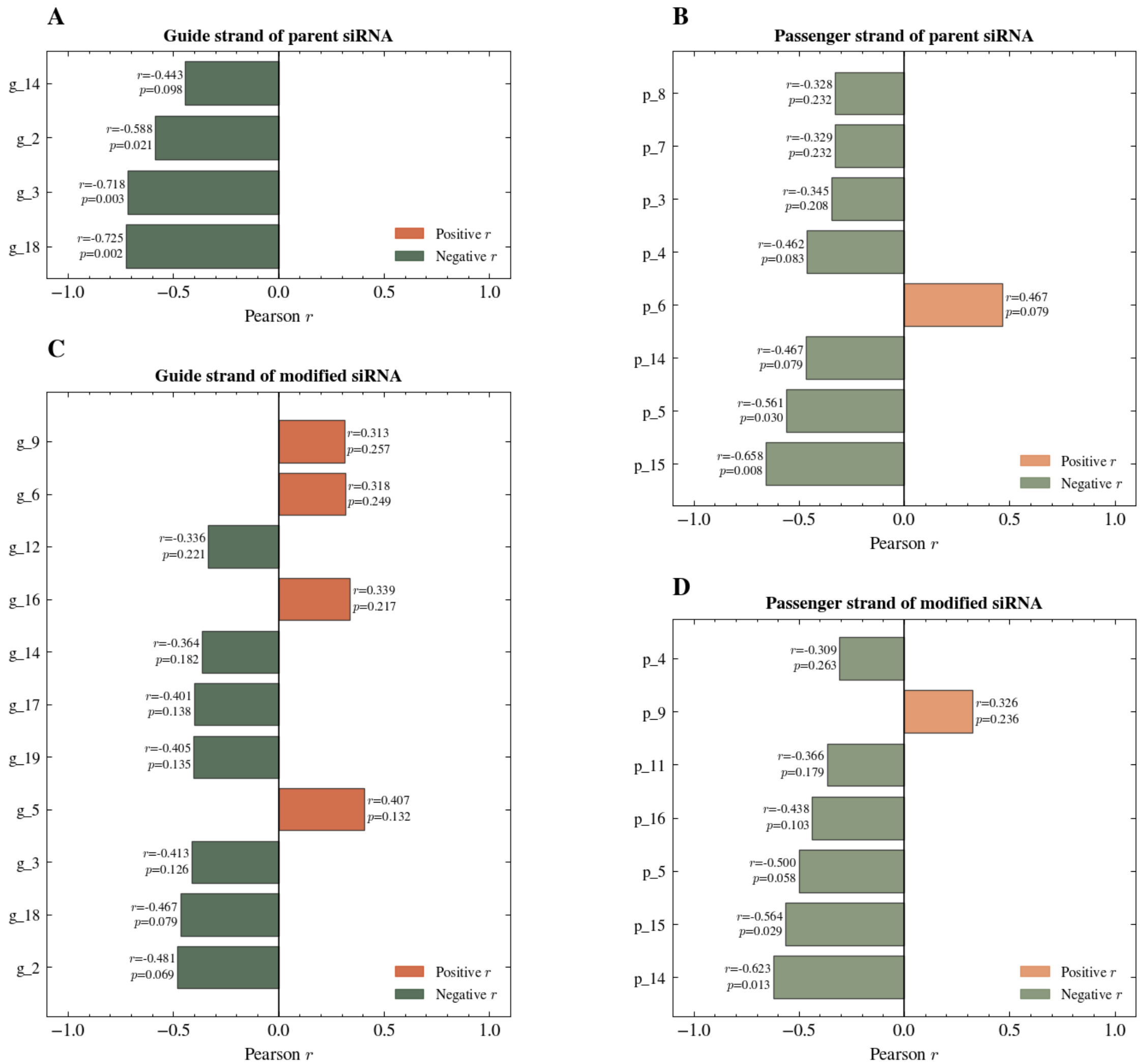

Figure S7. Position-specific Pearson correlation between the per-residue Rosetta energy (ΔE) and $log_{10}(\text{IC}_{50})$ for parent (unmodified) and chemically modified siRNAs, shown separately for the guide strand and the passenger strand.

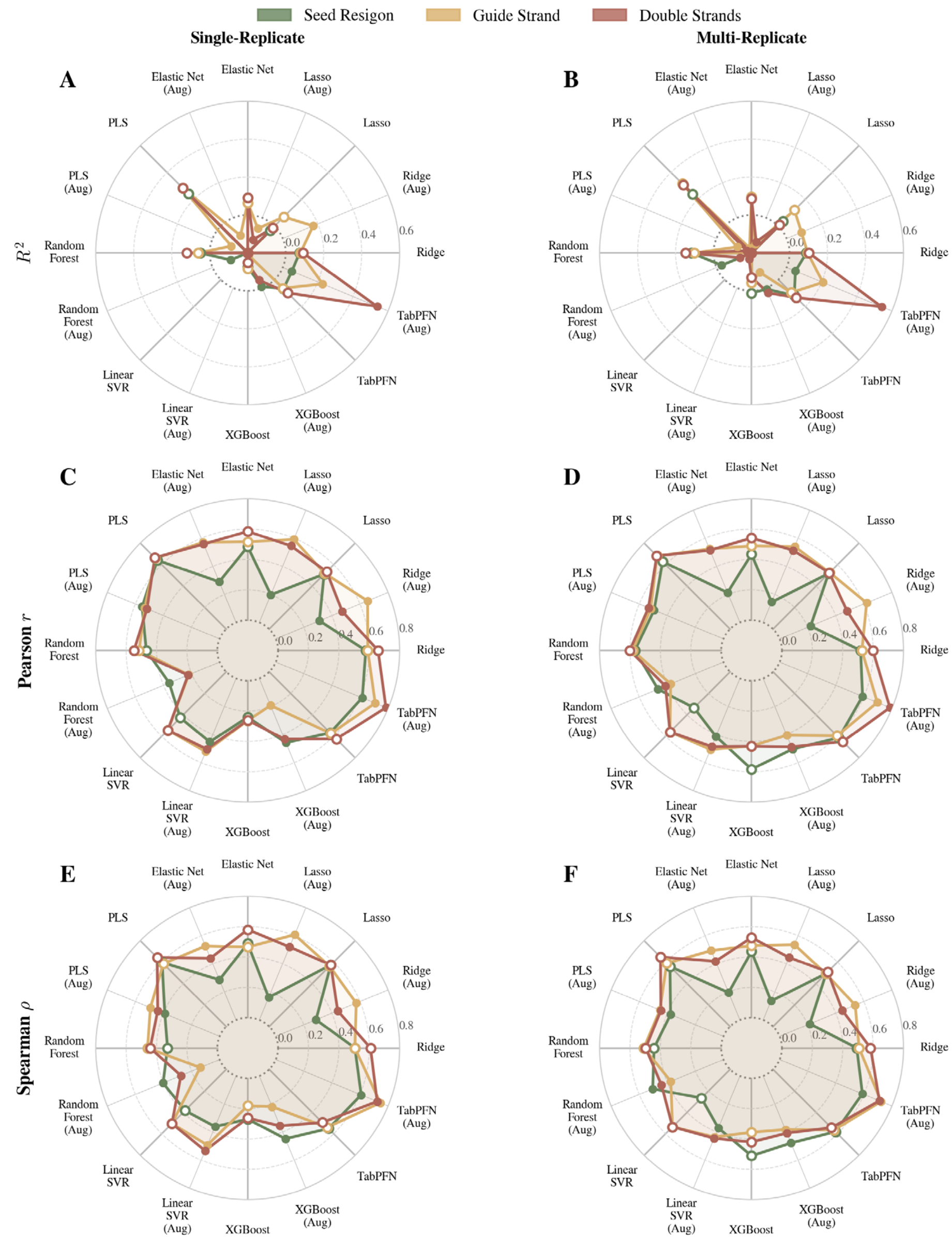

Figure S8. Radar chart comparison of predictive performance for different positional energy feature subsets in stratified 5-fold cross-validation, complementing the leave-one-out ablation analysis (Figure 6). *The evaluation metrics are* R^2 *(A, B), Pearson* r *(C, D), and Spearman* ρ *(E, F). Panels (A, C, E) show performance on the single-replicate test set, while panels (B, D, F) display performance on the multi-replicate test set. Three feature configurations are compared: full dual-strand features (guide- and passenger-strand features; red), guide-strand-only features (yellow), and seed-region-only features (green). Each axis corresponds to an evaluated machine learning or tabular deep learning model; a larger enclosed area indicates superior generalization across models. Solid markers represent models with Inference Augmentation; hollow markers represent models without augmentation.*

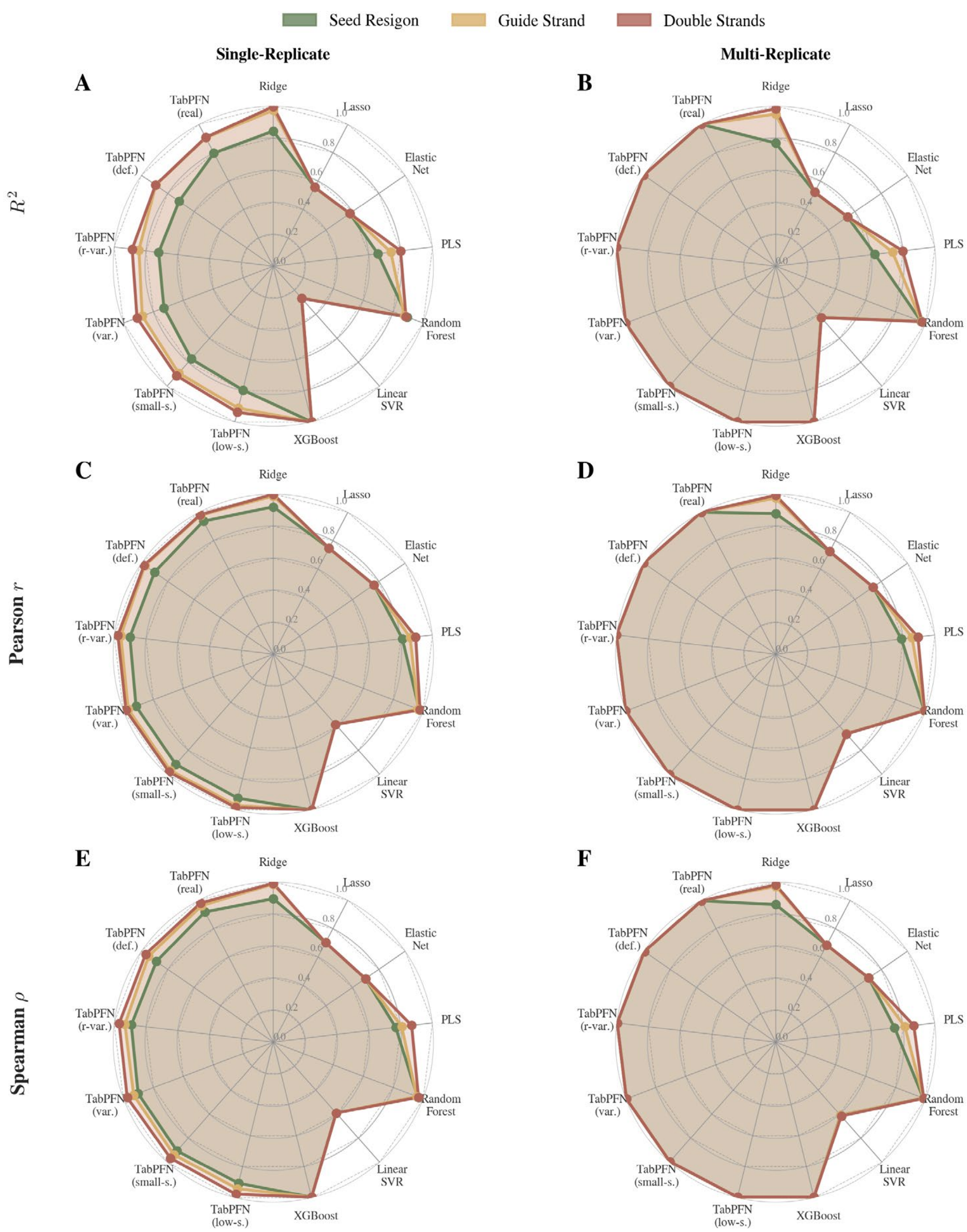

Figure S9. Radar chart comparison of predictive performance for different positional energy feature subsets under full-dataset fitting (training and evaluation on the complete dataset without a held-out test set). *The evaluation metrics are R^2 (A, B), Pearson r (C, D), and Spearman ρ (E, F). Panels (A, C, E) show performance on the single-replicate dataset, while panels (B, D, F) display performance on the multi-replicate dataset. Three feature configurations are compared: full dual-strand features (guide- and passenger-strand features; red), guide-strand-only features (yellow), and seed-region-only features (green). Each axis corresponds to an evaluated machine learning or tabular deep learning model; a larger enclosed area indicates superior predictive performance. Solid markers represent models trained with Inference Augmentation, whereas hollow markers designate models evaluated without Inference Augmentation.*

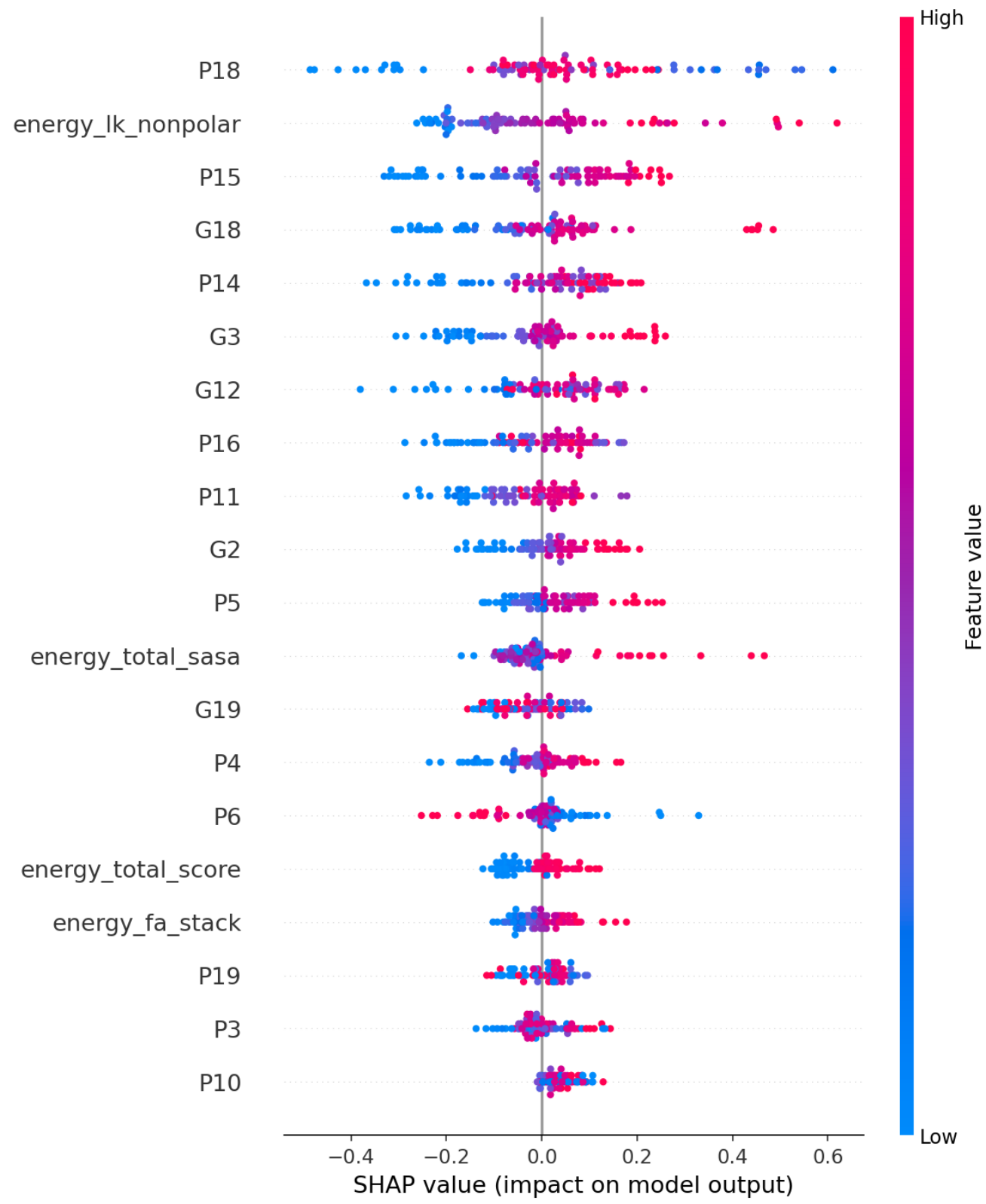

Figure S10. SHAP beeswarm summary plot showing the contribution of the top 20 Rosetta energy features to the Inference-Augmented TabPFN model's predictions of $-log_{10}(\mathrm{IC}_{50})$. *Features are ranked by mean absolute SHAP value (top to bottom); each point represents a single sample, coloured by relative feature value (red: high, blue: low).*

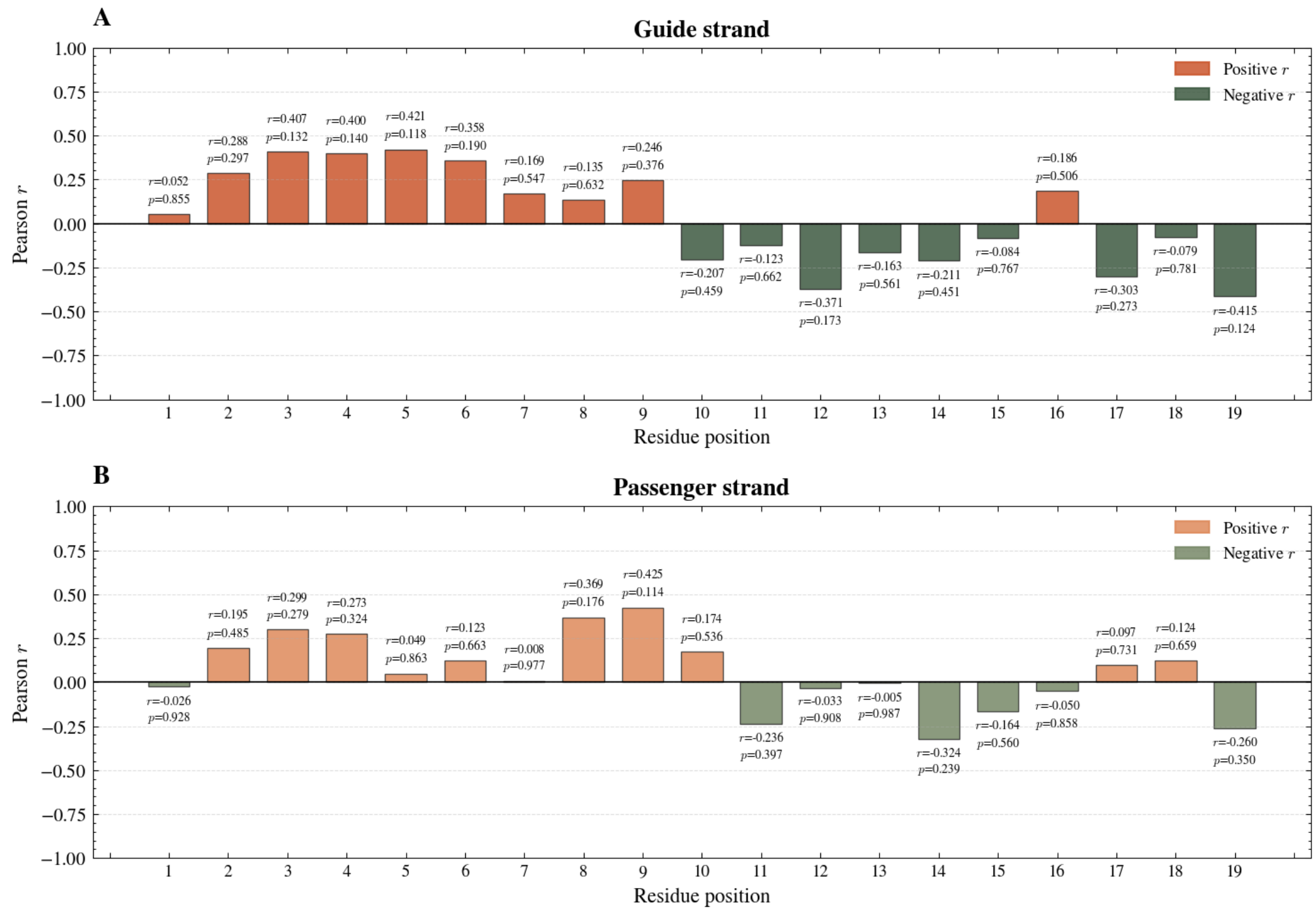

Figure S11. Position-specific Pearson correlation between the modification-induced Rosetta energy change ($\Delta\Delta E = \Delta E_{mod} - \Delta E_{unmod}$) and log_{10}(IC$_{50}$) across all nucleotide positions, shown separately for parent and modified siRNAs and for both the guide strand and the passenger strand.

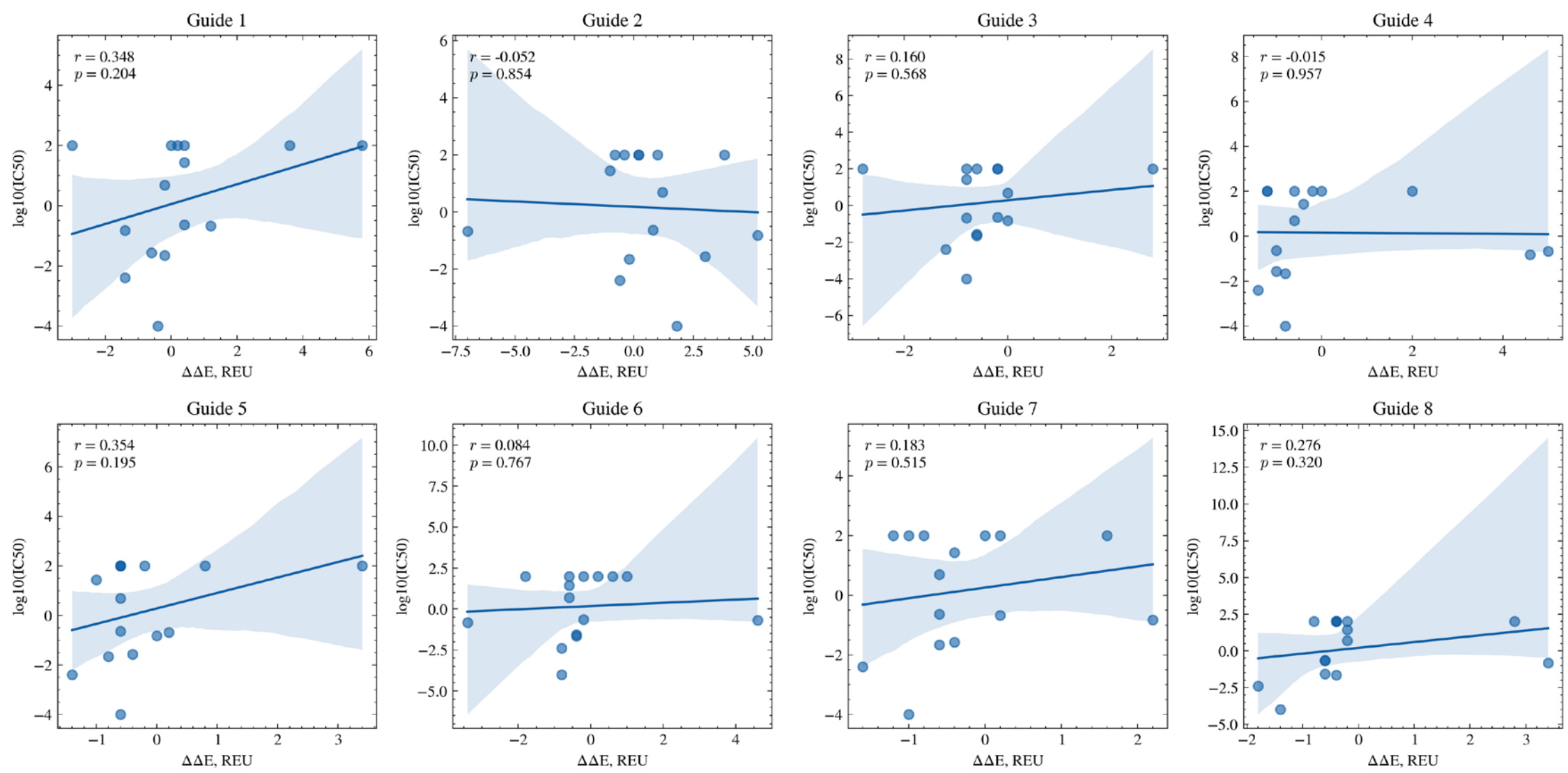

Figure S12 Pearson correlation between molecular dynamics (MD)-derived modification-induced energy changes ($\Delta\Delta E_{MD}$) and $log_{10}(\mathrm{IC}_{50})$ at seed-region positions (g2–g8 of the guide strand), reproduced from Kliuchnikov et al.[4]

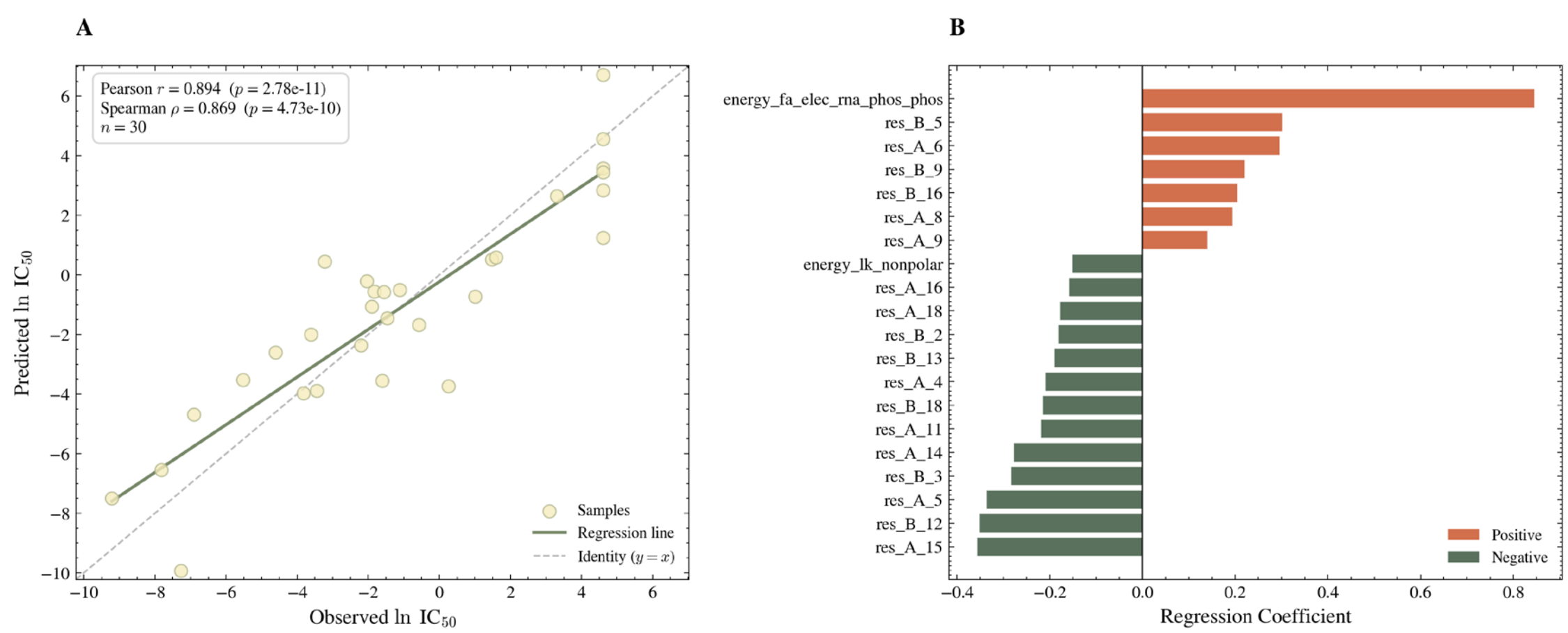

Figure S13. Performance and variable importance of the PLS model trained on Rosetta energy features using the full dataset (no held-out test set). (A) Scatter plot of predicted versus experimental $log_e(\mathrm{IC}_{50})$, with Pearson $r = 0.894$ ($p = 2.8 \times 10^{-11}$) and Spearman ρ indicated; the dashed line represents perfect agreement ($y = x$). This surpasses the MD-derived PLS baseline ($r = 0.87$) from Kliuchnikov et al.[4] (B) Variable Importance in Projection (VIP) scores for the 20 most influential features of the PLS model, indicating the dominant contribution of whole-duplex energy terms and selected per-residue positions.

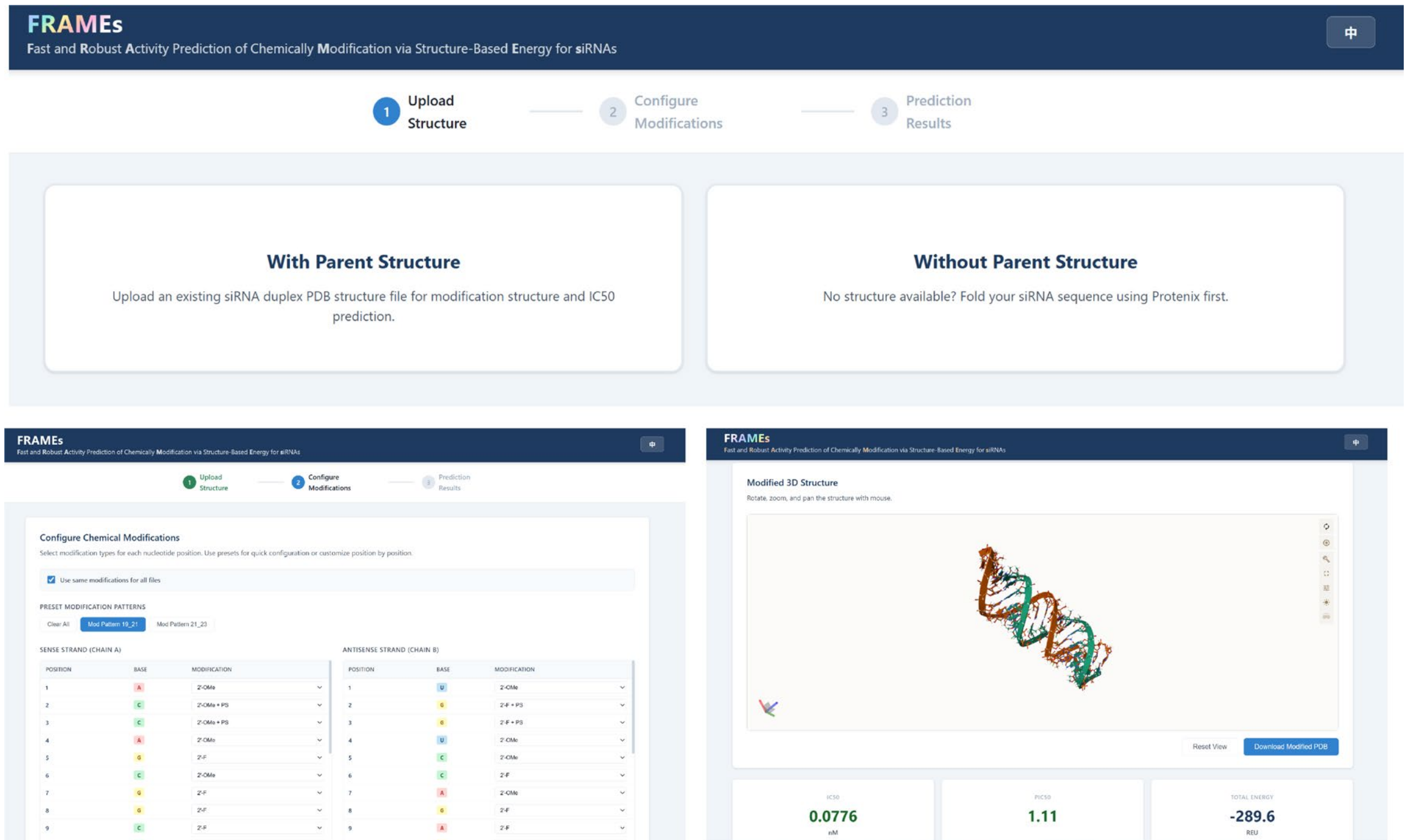

Figure S14. Screenshot of the FRAMEs web server. *The input panel (upper) accepts a PDB file of the unmodified siRNA structure and a strand-specific modification pattern (lower left). Upon submission, the results panel (lower right) displays the predicted pIC$_{50}$, the corresponding IC$_{50}$ estimate, and the Rosetta total energy of the refined duplex.*

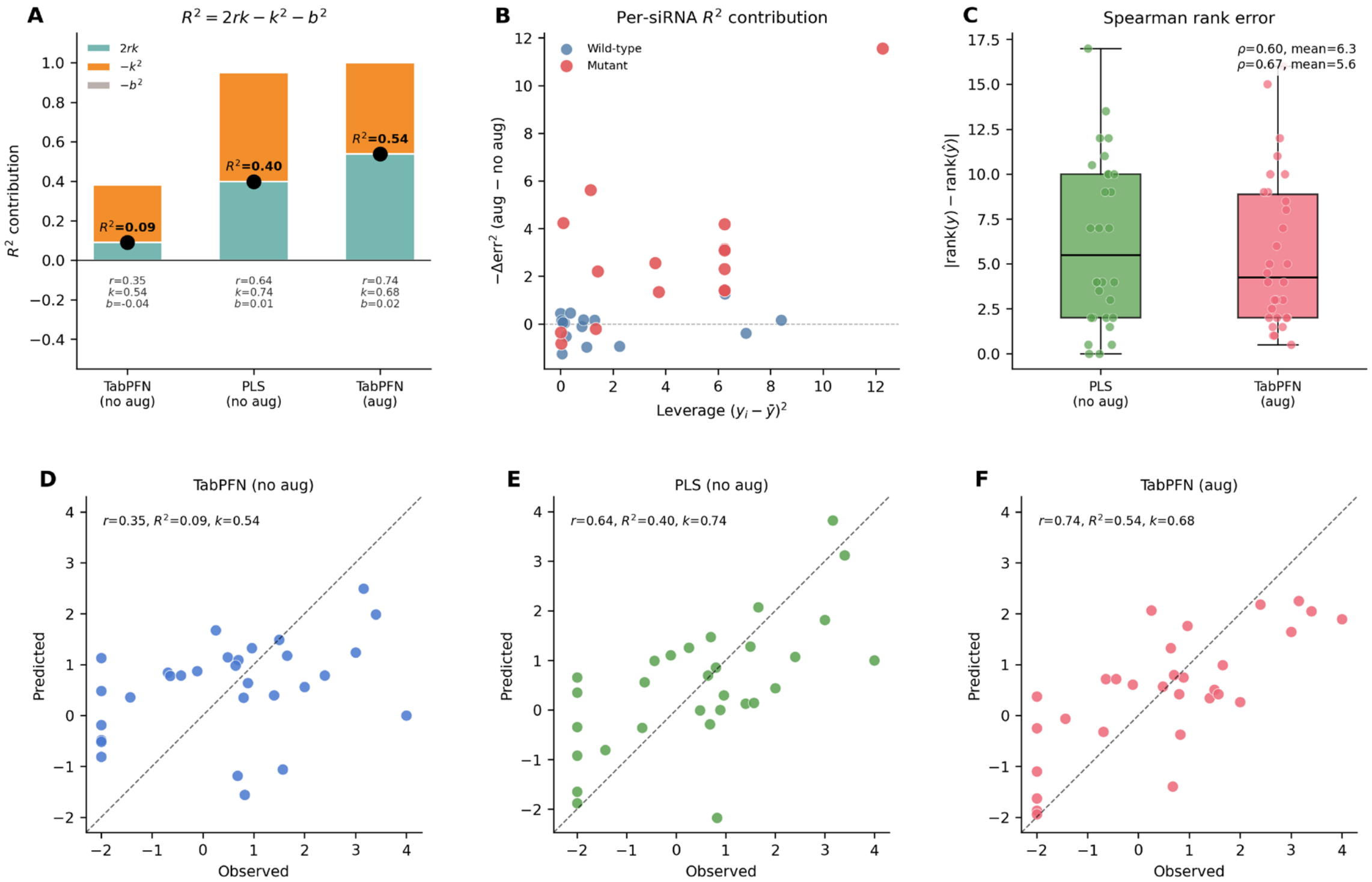

Figure S15. Attribution analysis of inference augmentation performance under leave-one-out cross-validation. (A) Decomposition of the coefficient of determination. (B) Per-siRNA contribution to R^2 improvement. (C) Spearman rank error distribution. (D) Predicted vs. observed (TabPFN, no augmentation). (D) Predicted vs. observed (PLS, no augmentation). (D) Predicted vs. observed (TabPFN, with augmentation).

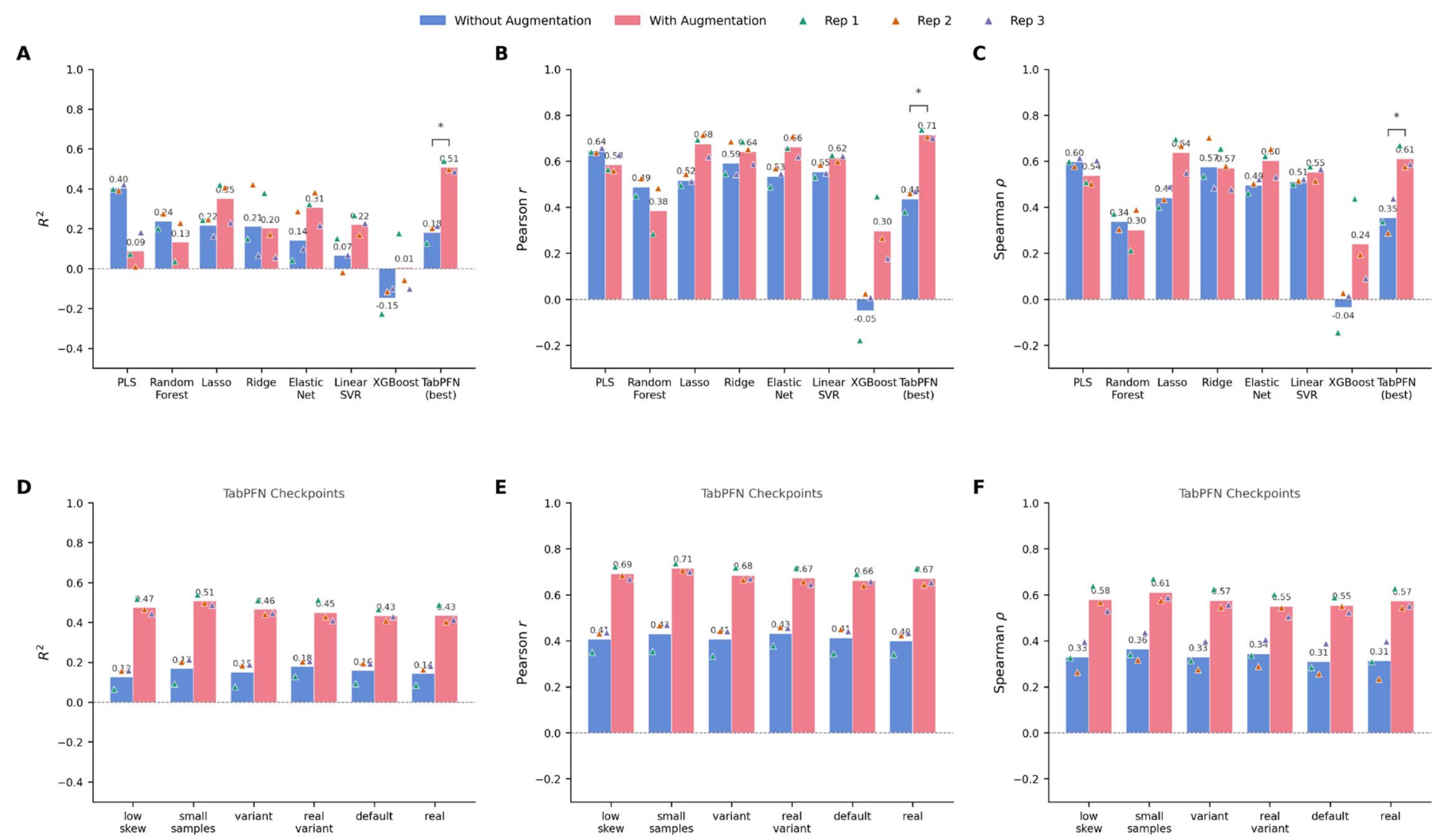

Figure S16. Leave-one-out cross-validation performance across three independent experimental repeats with randomized Rosetta energy minimization seeds. *(A–C) Bar charts showing R^2, Pearson r, and Spearman ρ, respectively, for all evaluated models without Inference Augmentation (blue) and with Inference Augmentation (red). "TabPFN (best)" refers to the highest-performing checkpoint. (D–F) Bar charts showing R^2, Pearson r, and Spearman ρ, respectively, for individual TabPFN checkpoints without augmentation (blue) and with Inference Augmentation (red). Significance was assessed by paired two-sided t-test: '*' $p < 0.05$.*

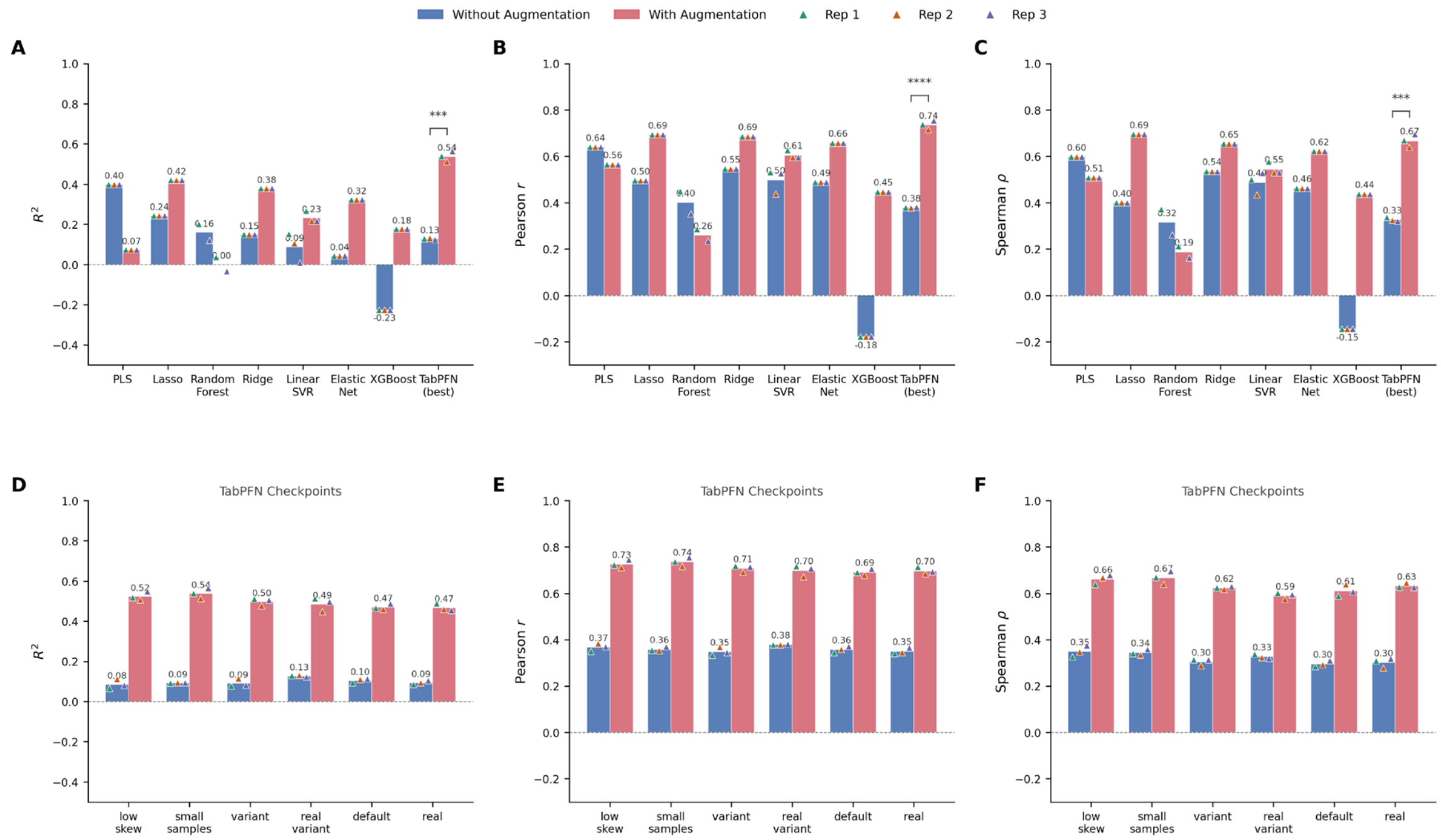

Figure S17. Leave-one-out cross-validation performance across three independent experimental repeats with randomized model training seeds. *(A–C) Bar charts showing R^2, Pearson r, and Spearman ρ, respectively, for all evaluated models without Inference Augmentation (blue) and with Inference Augmentation (red). "TabPFN (best)" refers to the highest-performing checkpoint. (D–F) Bar charts showing R^2, Pearson r, and Spearman ρ, respectively, for individual TabPFN checkpoints without augmentation (blue) and with Inference Augmentation (red). Significance was assessed by paired two-sided t-test: '****' indicates $p < 0.001$; '***' $p < 0.005$; '**' $p < 0.01$; '*' $p < 0.05$.*

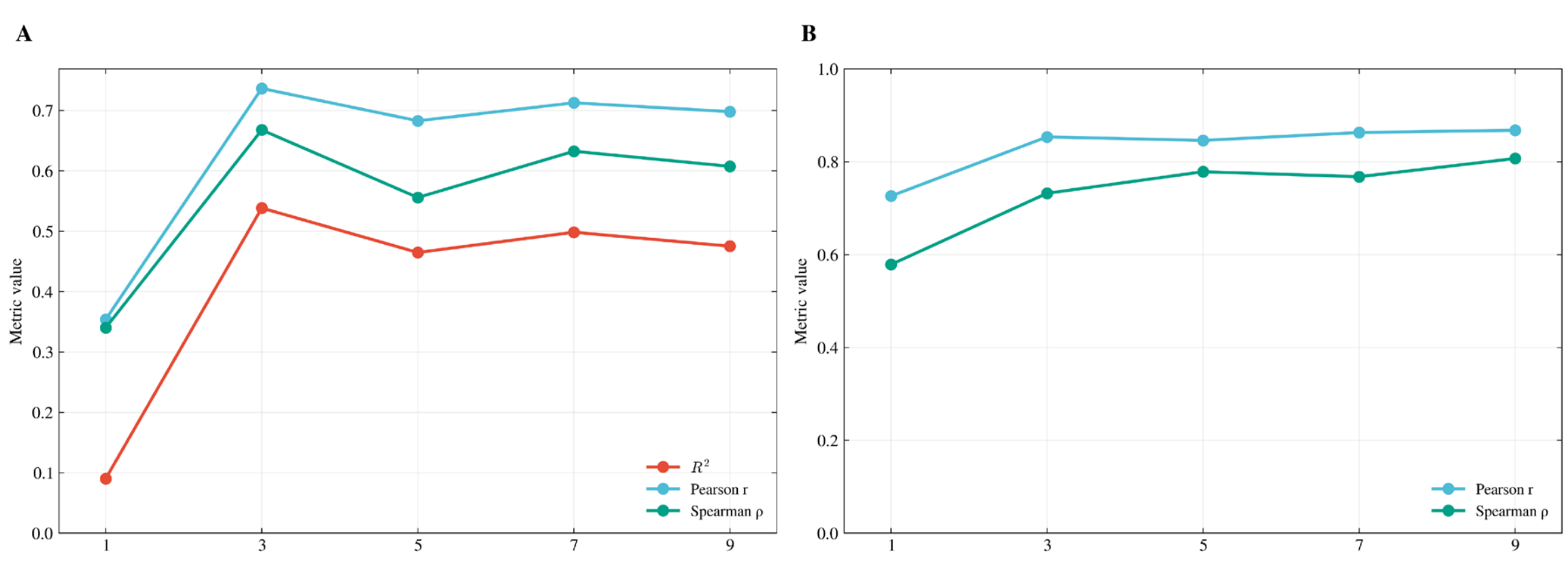

Figure S18. Effect of training repeat augmentation on siRNA activity prediction performance. *(A) Leave-one-out cross-validation performance. (B) External test set performance.*

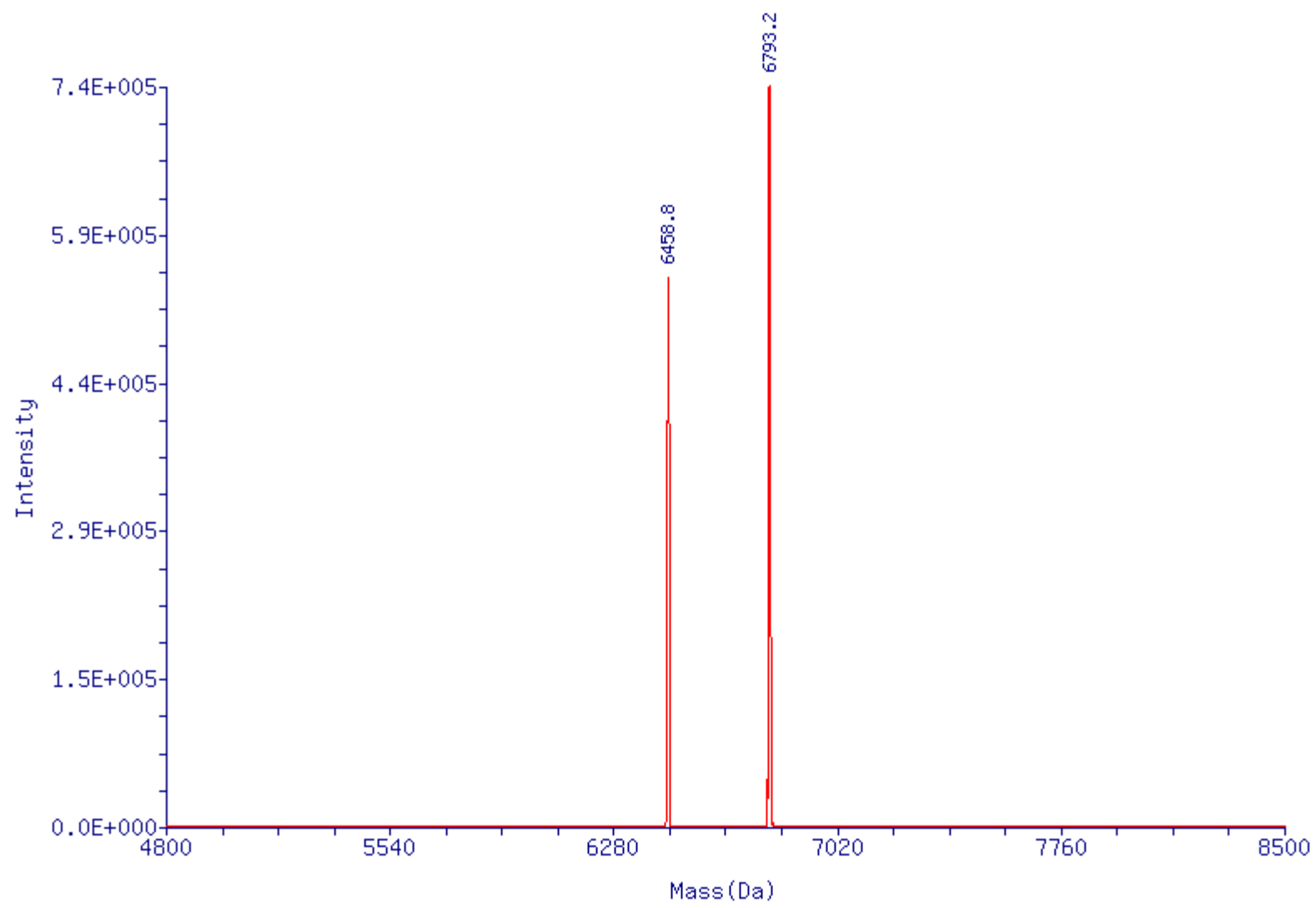

Figure S19. Mass spectrum of CDC20-1.

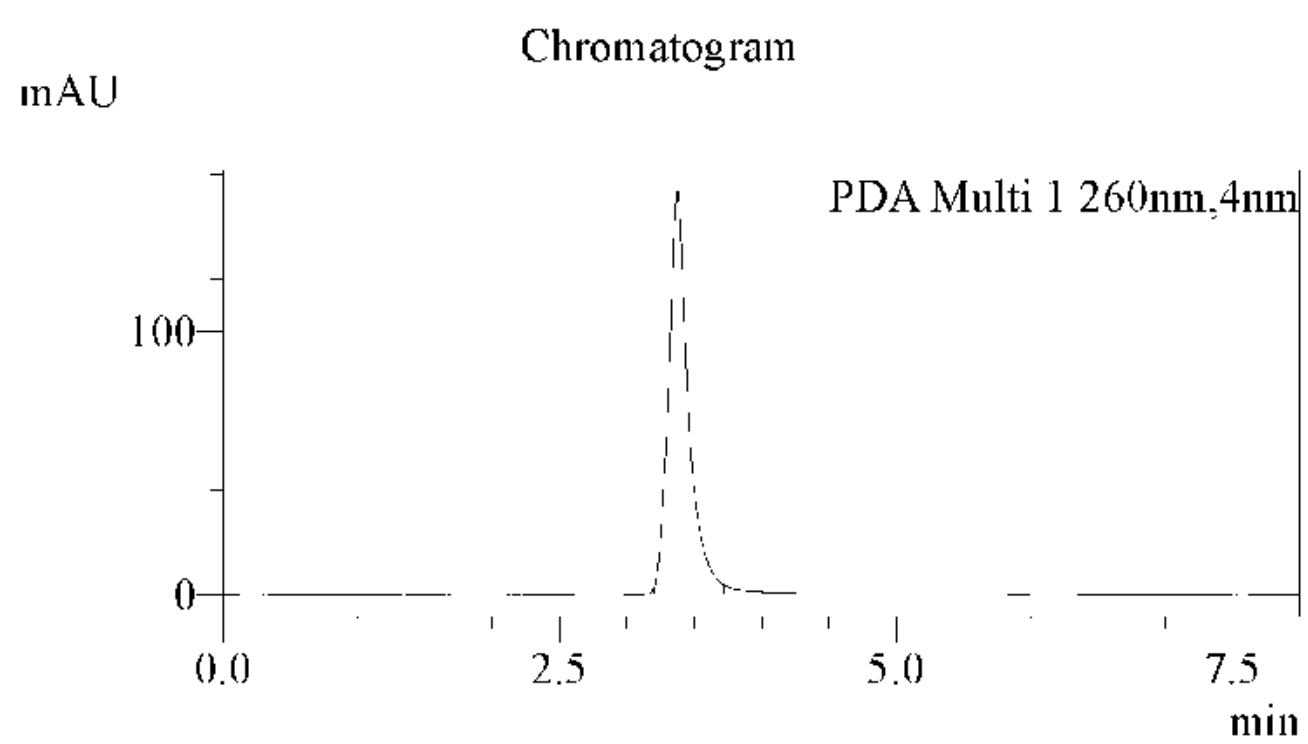

Peak Table

PDA Ch1 260nm

Peak#	Ret. Time	Area	Height	Area%
1	3.195	620	934	0.039
2	3.376	1563496	152864	98.209
3	3.739	27889	2970	1.752
Total		1592005	156768	100.000

Figure S20. HPLC chromatogram of CDC20-1.

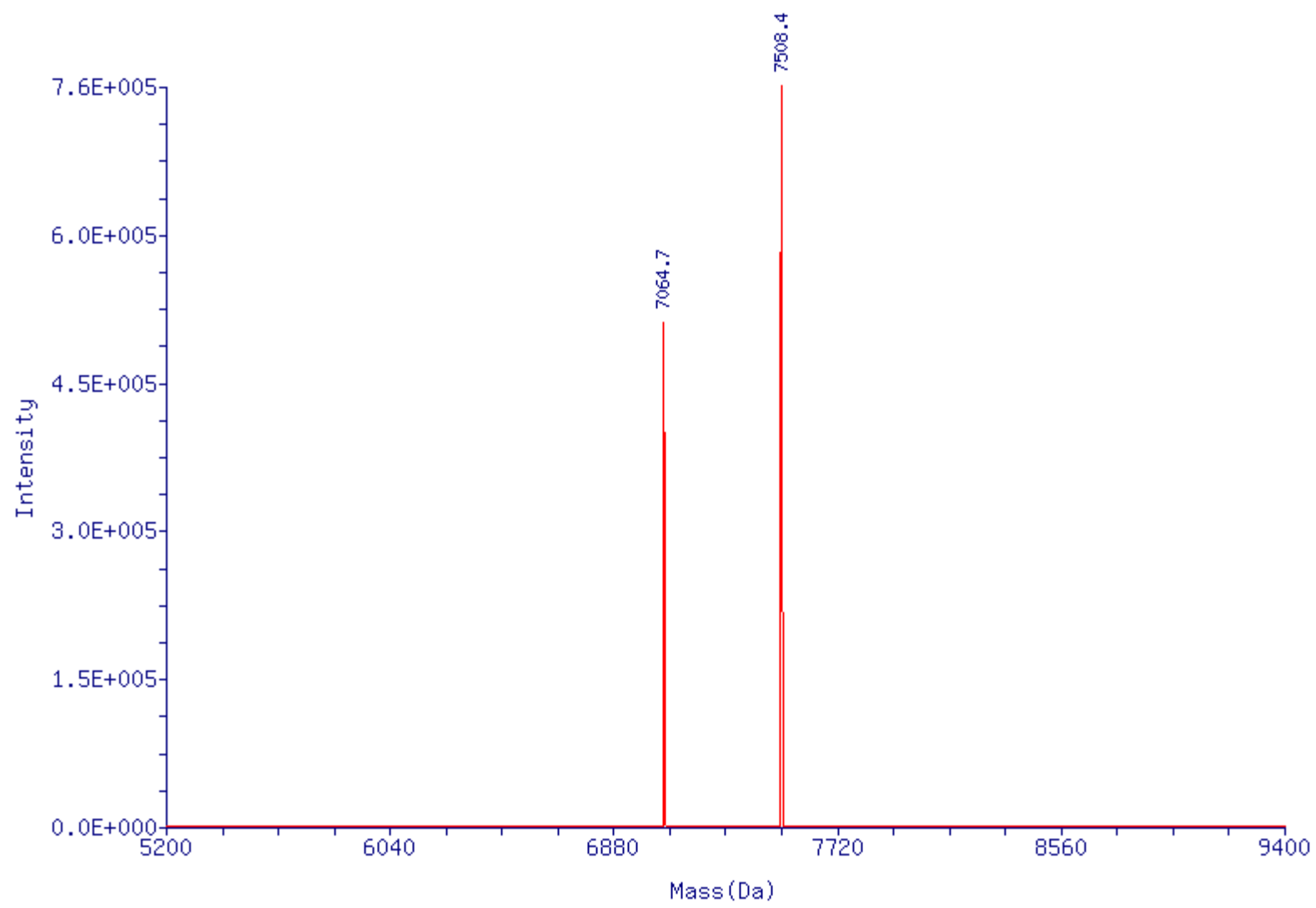

Figure S21. Mass spectrum of CDC20-2.

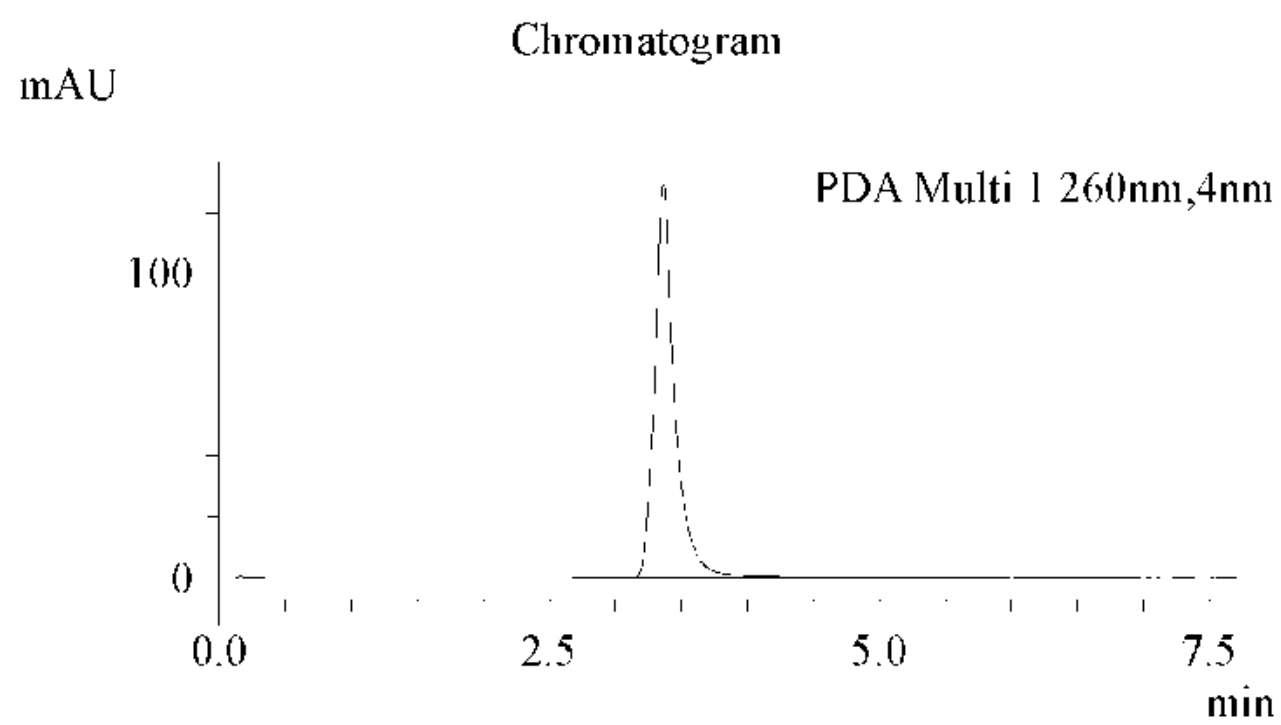

Peak Table

PDA Ch1 260nm

Peak#	Ret. Time	Area	Height	Area%
1	3.205	7189	3430	0.529
2	3.365	1320944	129435	97.116
3	3.696	32033	3338	2.355
Total		1360166	136204	100.000

Figure S22. HPLC chromatogram of CDC20-2.

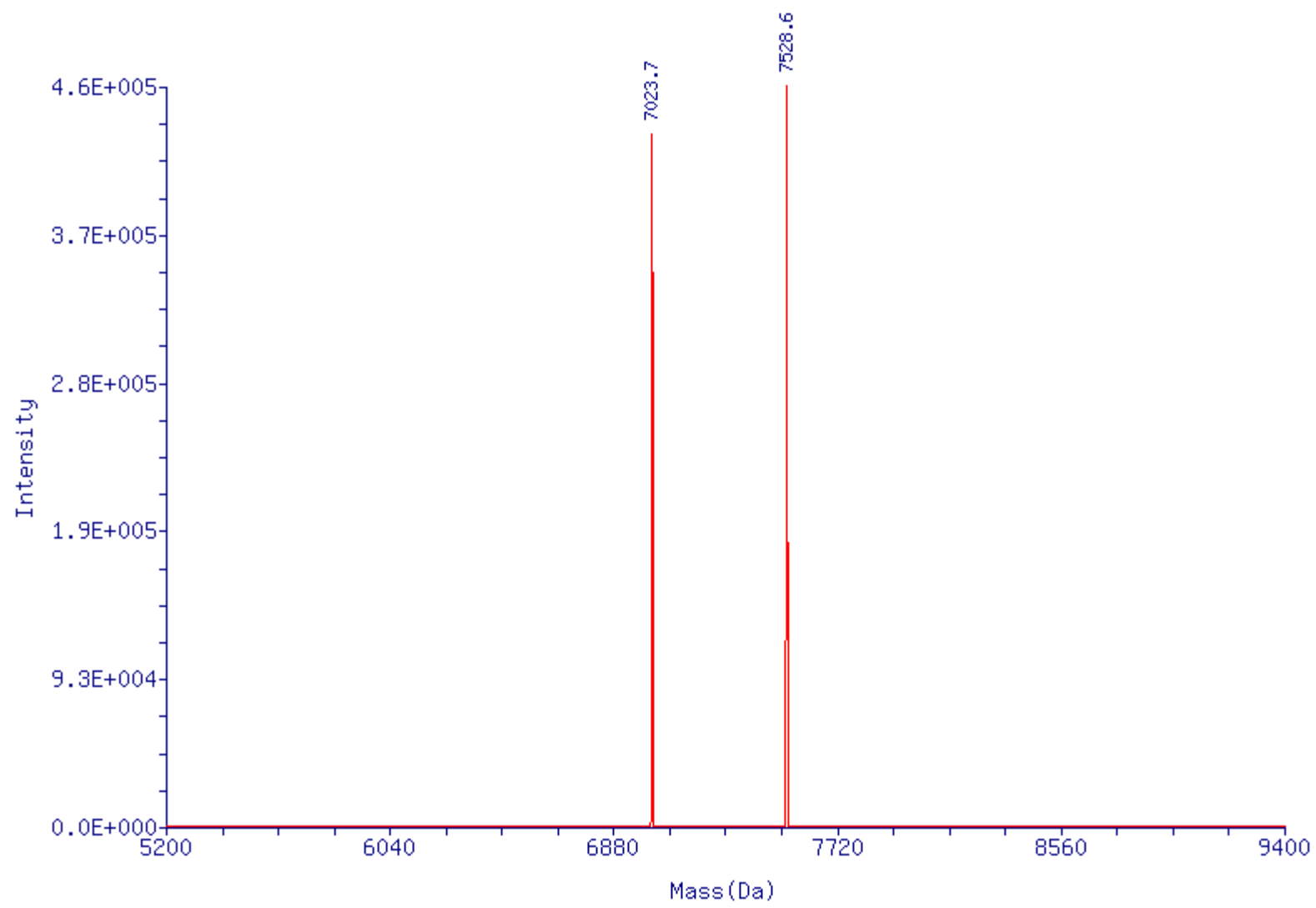

Figure S23. Mass spectrum of TOP2A-1.

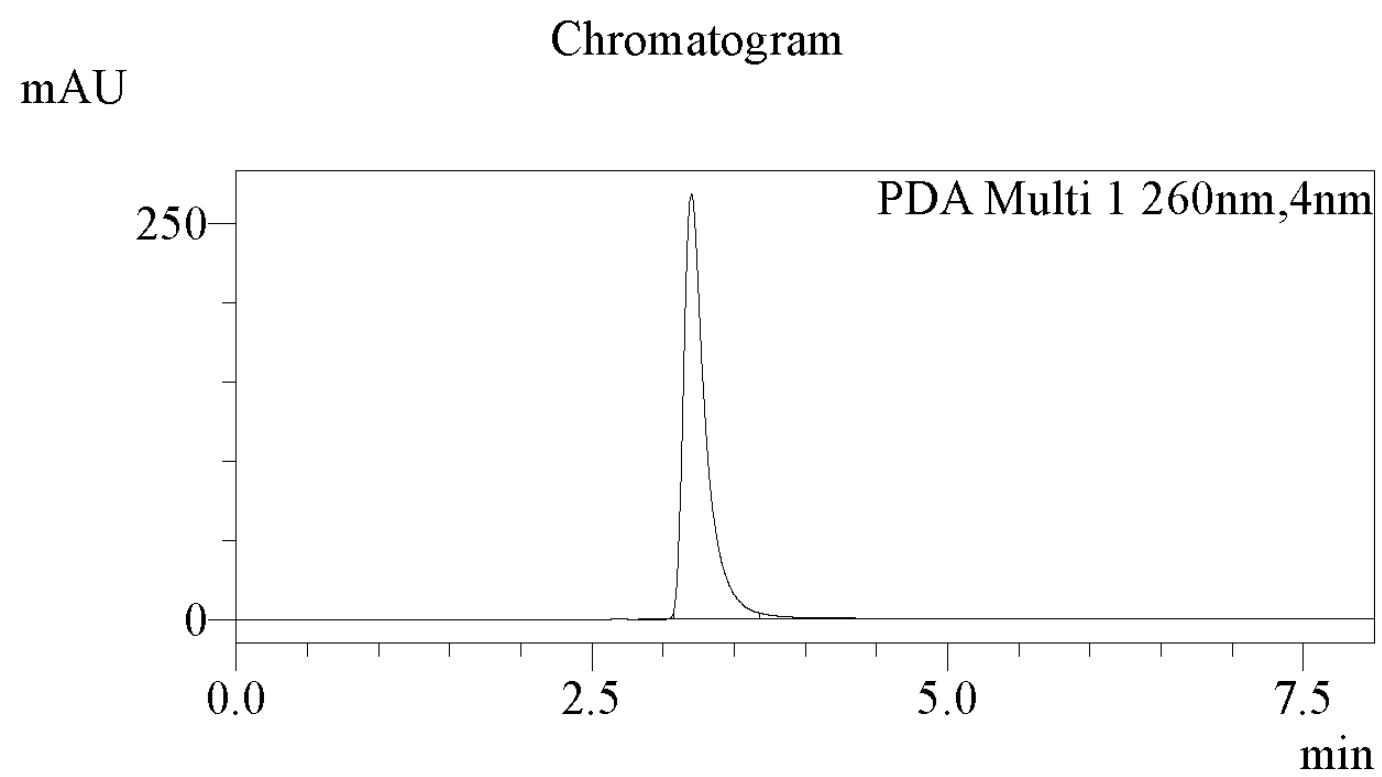

Peak Table

PDA Ch1 260nm

Peak#	Ret. Time	Area	Height	Area%
1	3.067	2118	1245	0.073
2	3.200	2877777	267910	98.781
3	3.696	33381	3142	1.146
Total		2913276	272298	100.000

Figure S24. HPLC chromatogram of TOP2A-1.

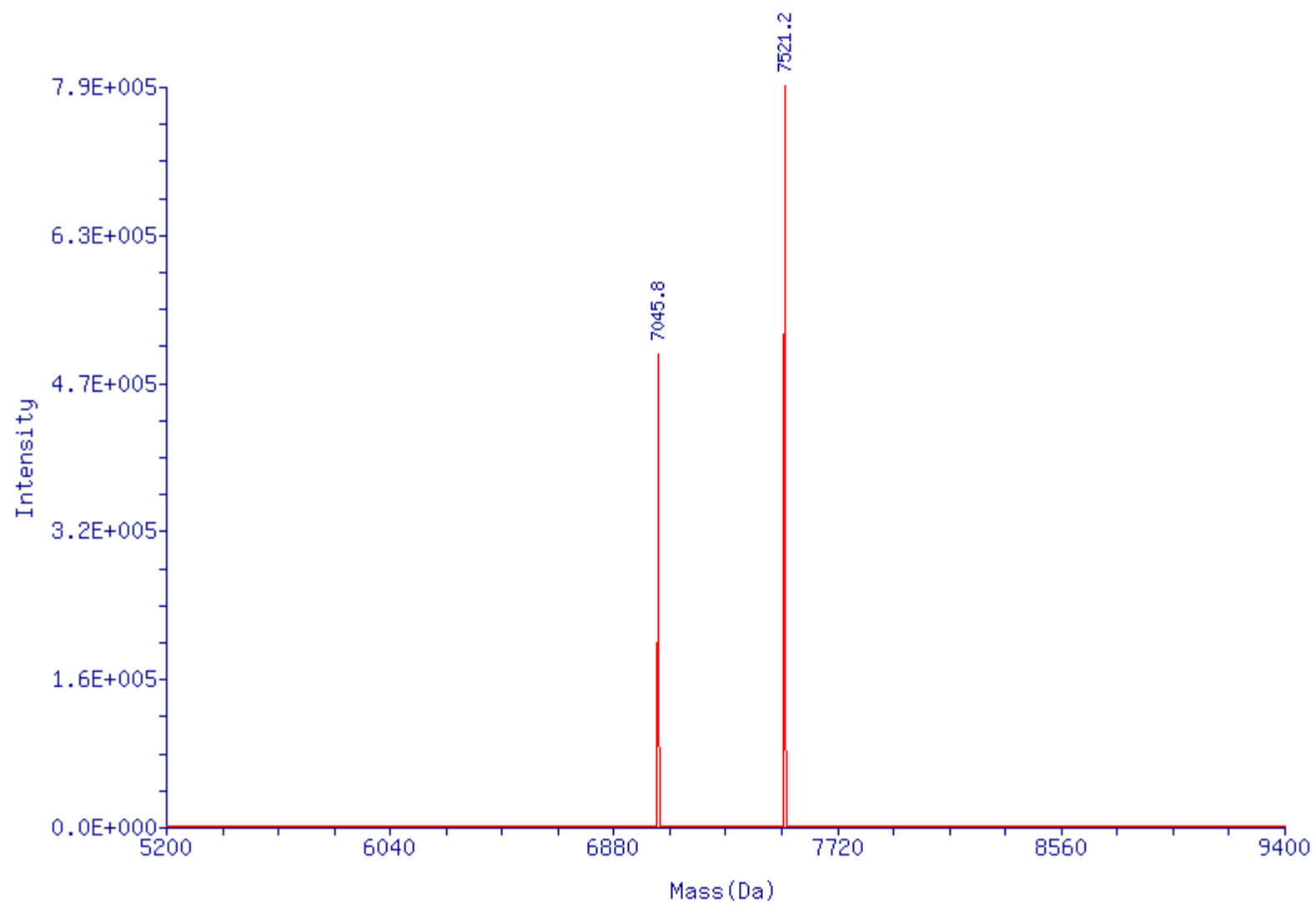

Figure S25. Mass spectrum of TOP2A-2.

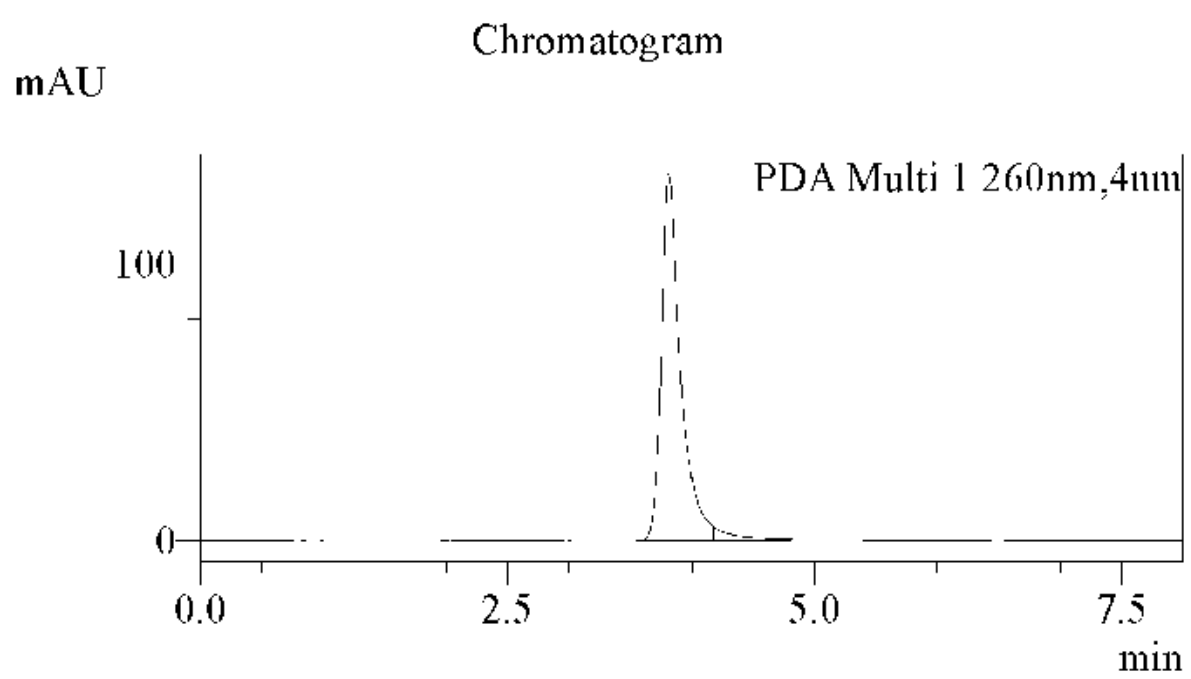

Peak Table

PDA Ch1 260nm

Peak#	Ret. Time	Area	Height	Area%
1	3.664	9331	3611	0.621
2	3.813	1429559	132572	95.196
3	4.187	62809	4643	4.183
Total		1501699	140826	100.000

Figure S26. HPLC chromatogram of TOP2A-2.

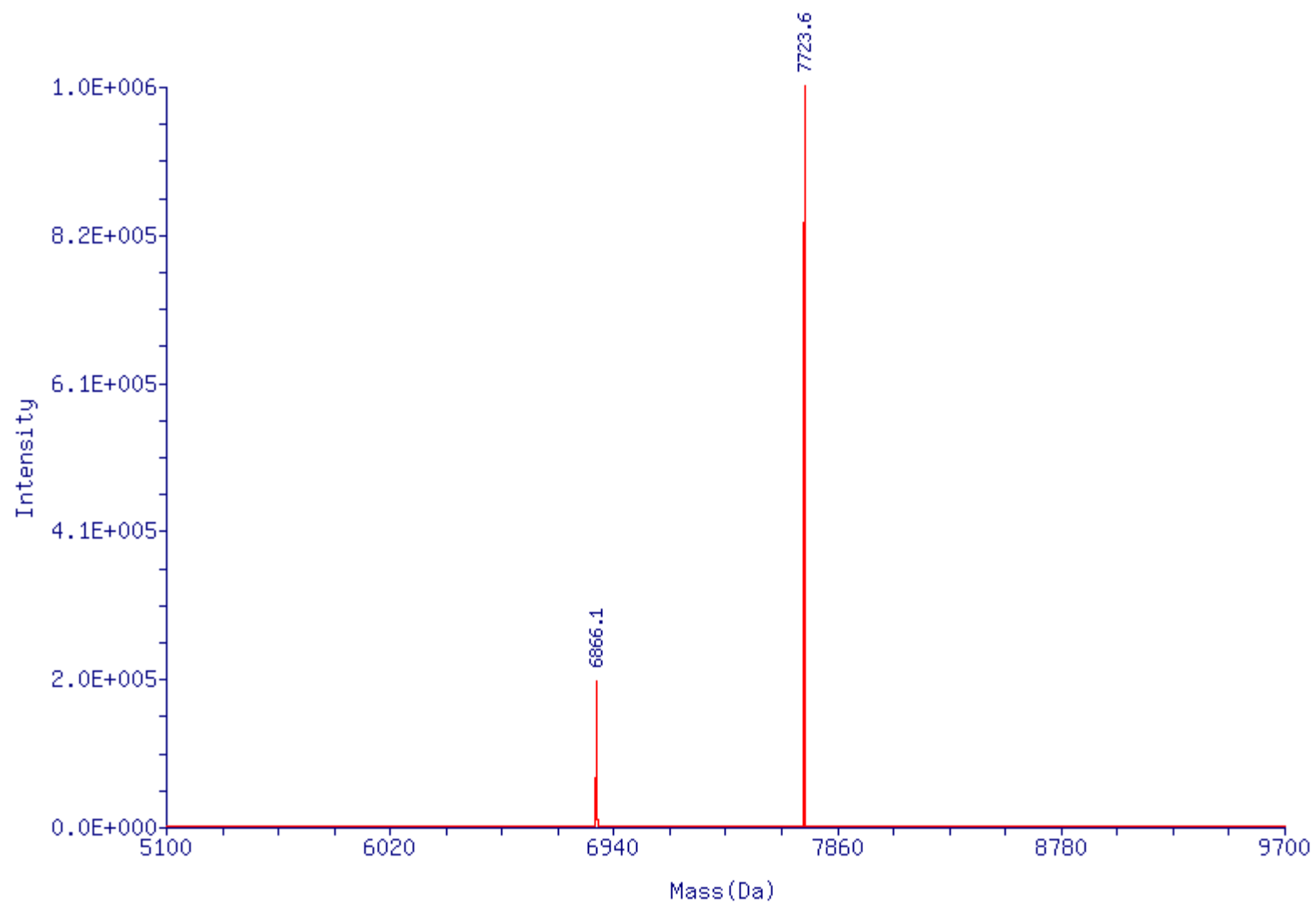

Figure S27. Mass spectrum of TOP2A-3.

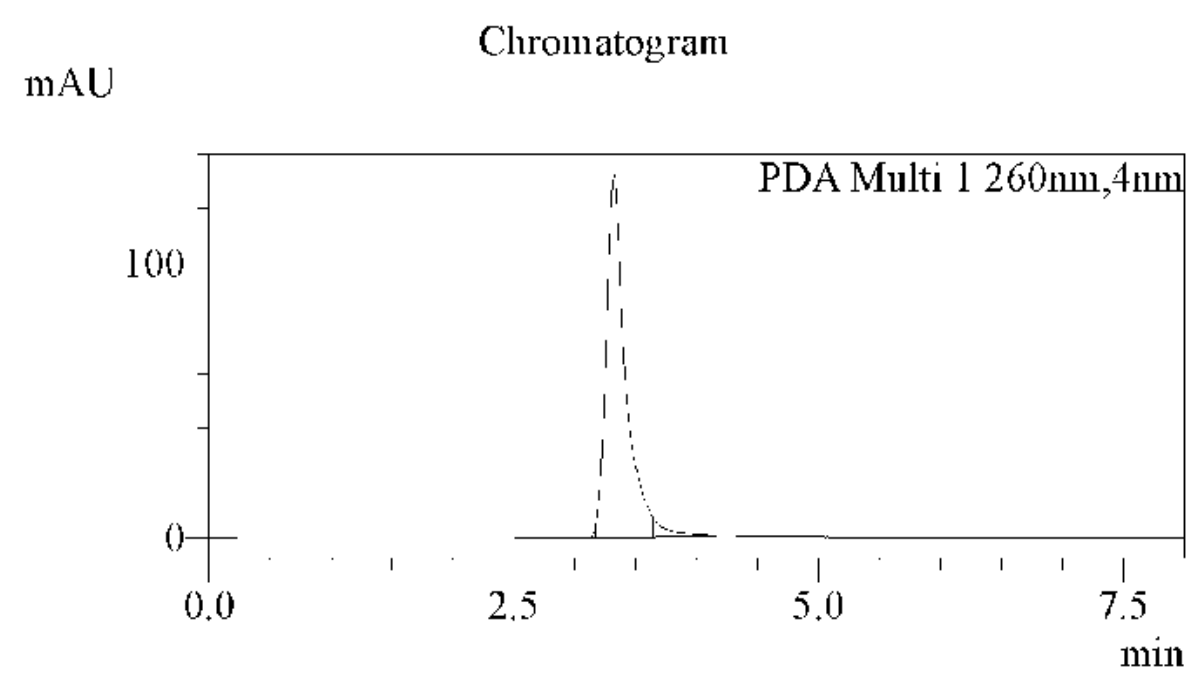

Peak Table

PDA Ch1 260nm

Peak#	Ret. Time	Area	Height	Area%
1	3.163	4615	1922	0.310
2	3.319	1428678	132493	95.929
3	3.653	56021	6439	3.762
Total		1489313	140854	100.000

Figure S28. HPLC chromatogram of TOP2A-3.

Supplemental Tables

Table S1. Leave-one-out cross-validation performance on the single-replicate test set with Inference Augmentation. Reported metrics are MSE, R^2, Pearson r, and Spearman ρ. *"Real" denotes augmentation with genuine Rosetta energy-calculation replicates; "Syn (σ)" denotes synthetic Gaussian-noise augmentation at the indicated noise level σ (as a fraction of the per-feature standard deviation).*

Table S2. Five-fold cross-validation performance on the single-replicate test set with Inference Augmentation. Reported metrics are MSE, R^2, Pearson r, and Spearman ρ. *"Real" denotes augmentation with genuine Rosetta energy-calculation replicates; "Syn (σ)" denotes synthetic Gaussian-noise augmentation at the indicated noise level σ (as a fraction of the per-feature standard deviation).*

Table S3. Hyperparameter search grids used for traditional machine learning models during 5-fold cross-validation*. Optimal hyperparameters were selected via grid search with cross-validated scoring on the training folds.*

Model	Hyperparameter	Search Values
XGBoost	*n_estimators*	50, 100, 200
	max_depth	3, 6, 9
	learning_rate	0.01, 0.1, 0.2
Ridge Regression	*alpha*	0.1, 1.0, 10.0, 100.0
Random Forest	*n_estimators*	50, 100
	max_depth	None, 10, 20
	min_samples_split	2, 5
PLS Regression	*n_components*	2, 3, 4, 5, 6
Lasso Regression	*alpha*	0.01, 0.1, 1.0, 10.0
Elastic Net	*alpha*	0.01, 0.1, 1.0
	l1_ratio	0.2, 0.5, 0.8
Linear SVR	*C*	0.1, 1.0, 10.0, 100.0
	loss	*epsilon_insensitive*, *squared_epsilon_insensitive*

Table S4. Reaction system for removal of genomic DNA (gDNA) from total RNA prior to reverse transcription.

Reagent	Volume
gDNA Clean Reaction Mix	2 μL
Total RNA	1 μg
RNase-free ddH_2O	to 10 μL
Thermal conditions: 42C, 2 min; then 4C, hold	

Table S5. Reverse transcription reaction system used with the Evo M-MLV RT Mix Kit with gDNA Clean for qPCR.

Reagent	Volume
Product from gDNA-removal step (Table S4)	10 μL
5× Evo M-MLV RT Reaction Mix	4 μL
RNase-free ddH_2O	6 μL
Total	20 μL
Thermal conditions: 37C, 15 min; 85C, 5 s; then 4C, hold	

Table S6. RT-qPCR reaction system (20 μL total volume).

Component	Volume	Final Concentration
2× SYBR Green Pro Taq HS Premix	10 μL	1×
Forward primer (10 μM)	0.4 μL	0.2 μM
Reverse primer (10 μM)	0.4 μL	0.2 μM
cDNA template	2 μL	—
RNase-free ddH_2O	to 20 μL	—

Table S7. Primer sequences used for RT-qPCR quantification of CDC20, TOP2A, and the reference gene GAPDH.

Gene	Forward Primer (5’→3’)	Reverse Primer (5’→3’)
CDC20	CGCTATATCCCCCATCGCAG	GATGTTCCTTCTTGGTGGGC
TOP2A	CAGGGTTCTTGAGCCCCTTC	ATGTAGGTGTCTGGGCGGA
GAPDH	ACGGATTTGGTCGTATTGGG	CGCTCCTGGAAGATGGTGAT

References

1. Murphy, A.H. (1988). Skill scores based on the mean square error and their relationships to the correlation coefficient. Monthly weather review *116*, 2417–2424.

2. Gupta, H.V., Kling, H., Yilmaz, K.K., and Martinez, G.F. (2009). Decomposition of the mean squared error and NSE performance criteria: Implications for improving hydrological modelling. Journal of Hydrology *377*, 80–91. https://doi.org/10.1016/j.jhydrol.2009.08.003.

3. Xu, S., Feng, Q., Qiao, L., Wu, H., Shen, T., Cheng, Y., Zheng, S., and Sun, S. (2025). Benchmarking all-atom biomolecular structure prediction with FoldBench. Nature Communications.

4. Kliuchnikov, E., Maksudov, F., Zuber, J., Hyde, S., Castoreno, A., Waldron, S., Schlegel, M.K., Marx, K.A., Maier, M.A., and Barsegov, V. (2025). Improving the potency prediction for chemically modified siRNAs through insights from molecular modeling of individual sequence positions. Molecular Therapy Nucleic Acids *36*. https://doi.org/10.1016/j.omtn.2024.102415.
